# Synthesis and Cytotoxicity Evaluation of DOTA-Conjugates of Ursolic Acid

**DOI:** 10.3390/molecules24122254

**Published:** 2019-06-17

**Authors:** Michael Kahnt, Sophie Hoenke, Lucie Fischer, Ahmed Al-Harrasi, René Csuk

**Affiliations:** 1 Organic Chemistry, Martin-Luther-University Halle-Wittenberg, Kurt-Mothes-Str. 2, D-06120 Halle (Saale), Germany; michael.kahnt@chemie.uni-halle.de (M.K.); sophie.hoenke@chemie.uni-halle.de (S.H.); lucie.fischer2018@gmx.de (L.F.); 2 Natural and Medical Sciences Research Center, University of Nizwa, PO Box 33, Birkat Al-Mauz, Nizwa 616, Oman; aharrasi@unizwa.edu.om

**Keywords:** ursolic acid, DOTA, triterpenoids, cytotoxicity

## Abstract

In this study, we report the synthesis of several amine-spacered conjugates of ursolic acid (UA) and 1,4,7,10-tetraazacyclododecane-1,4,7,10-tetraacetic acid (DOTA). Thus, a total of 11 UA-DOTA conjugates were prepared holding various oligo-methylene diamine spacers as well as different substituents at the acetate units of DOTA including *tert*-butyl, benzyl, and allyl esters. Furthermore, three synthetic approaches were compared for the ethylenediamine-spacered conjugate **29** regarding reaction steps, yields, and precursor availability. The prepared conjugates were investigated regarding cytotoxicity using SRB assays and a set of human tumor cell lines. The highest cytotoxicity was observed for piperazinyl spacered compound **22**. Thereby, EC_50_ values of 1.5 µM (for A375 melanoma) and 1.7 µM (for A2780 ovarian carcinoma) were determined. Conjugates **22** and **24** were selected for further cytotoxicity investigations including fluorescence microscopy, annexin V assays and cell cycle analysis.

## 1. Introduction

**Figure 1:**
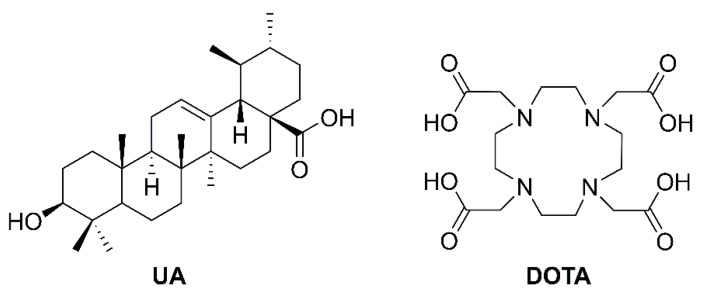
Structures of ursolic acid (UA) and 1,4,7,10-tetraazacyclododecane-1,4,7,10-tetraacetic acid (DOTA).

Despite all medical advances in tumor therapy, cancer is still one of the most prevalent diseases worldwide, with 9.6 million cancer-related deaths counted in 2018 [1]. The research of novel therapeutic approaches and potent chemotherapeutic agents are important contributions in the battle against cancer. However, diagnosis is a prerequisite for successful treatment since an early detection of cancer cells can often significantly reduce the pathogenicity of a tumor and increase the healing rate. One molecule, which made significant impact on the field of diagnostic imaging in the past decades, is the EDTA-related macrocyclic chelator 1,4,7,10-tetraazacyclododecane-1,4,7,10-tetraacetic acid (DOTA, Figure 1) [2,3].

DOTA-derivatives and complexes thereof are widely used for molecular imaging, especially for the medical diagnosis of cancer [2,3]. By variation of the coordinated metal ion or substituents, they are applicable for a number of imaging techniques, such as magnetic resonance imaging (MRI) [2,3,4], positron emission tomography (PET) [2,3,5,6] and single photon emission computed tomography (SPECT) [2,3,7]. Because of this versatility, a crossover application of DOTA-derivatives as multimodal contrast agents for combined imaging modalities such as PET/MRI or PET/CT is possible and has already been described in the literature [2,3].

Although DOTA and derivatives thereof have a wide range of uses in diagnostic imaging, there are virtually no references for applications in the therapy of cancer. Therefore, we decided to prepare possible cytotoxic DOTA-derivatives by linkage with an ursolic acid backbone. Ursolic acid (UA, Figure 1) is a natural occurring triterpenoic acid with promising pharmacological properties, being widely distributed in various plants and fruits, such as rosemary [8], sage [8], oleander [9], and apples [10,11]. A wide range of biological activities, including antidiabetic, anti-inflammatory, antibacterial, and anticancer effects have been credited to UA and structurally-related derivatives [12,13,14]. Many structural modifications have been described in the literature starting from ursolic acid with various impacts on cytotoxic properties [12,15,16,17,18]. Structure activity investigations concerning modifications at C-3 of UA revealed the presence of an acetyloxy group to be beneficial for obtaining high anti-tumor activity [18]. Furthermore, it has been shown that the modification of C-28 with a piperazine moiety had a positive influence on the cytotoxic properties of ursolic acid [19,20]. Previously, we also have shown oligo-methylene diamine derived carboxamides of ursolic acid to be of high cytotoxicity [21]. Keeping these structure activity relationships in mind, we considered 3-acetyloxy protected and C-28 modified UA derivatives a convenient starting point for the preparation of cytotoxic oligo-methylene diamine spacered DOTA conjugates.

## 2. Results and Discussion

**Scheme 1:**
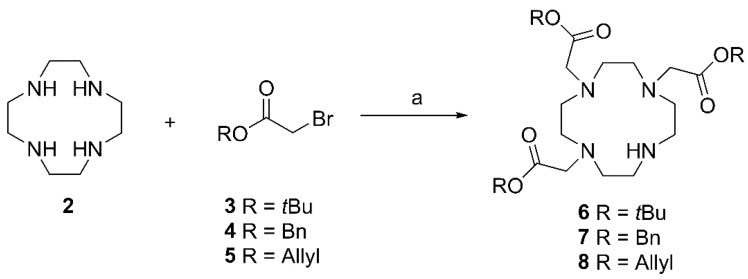
Synthesis of DOTA precursors **6**–**8**: (a) NaHCO_3_, MeCN, 25 °C, 48 h, yield: 50% (**6**), 68% (**7**), 63% (**8**).

The synthesis of UA-DOTA conjugates started with the structural modification of cyclen (**2**) as illustrated in Scheme 1. Treatment of **2** with 3 equiv. of sodium bicarbonate and 3 equiv. of the respective bromoacetic ester (**3**–**5**) in dry acetonitrile yielded triple substituted cyclen derivatives **6**–**8**, being ready to be coupled with ursolic acid. We decided to use *tert*-butyl, benzyl and allyl esters as protecting groups. Benzyl, as well as *tert*-butyl bromoacetate, were bought from commercial suppliers. Allyl bromoacetate was prepared from allylic alcohol and bromoacetyl bromide.

**Scheme 2:**
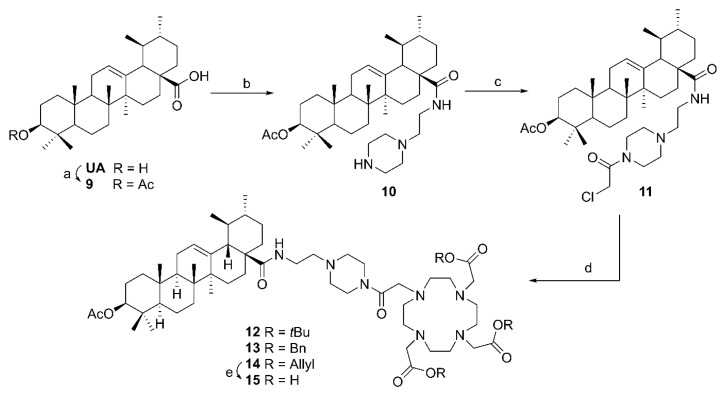
Synthesis of ursolic acid chelator conjugates **12**–**15**: (**a**) Ac_2_O, CH_2_Cl_2_, NEt_3_, 25 °C, 2 days, 82%; (**b**) oxalyl chloride, CH_2_Cl_2_, DMF, 0–25 °C, 1 h, then 1-(2-Aminoethyl)piperazine, CH_2_Cl_2_, 25 °C, 2 h, yield: 82%; (**c**) chloroacetyl chloride, CH_2_Cl_2_, NEt_3_, 25 °C, 30 min, yield: 91%; (**d**) K_2_CO_3_, KI, **6** (for **12**) or **7** (for **13**) or **8** (for **14**), MeCN, 25 °C, 48 h, yield: 54% (**12**), 82% (**13**), and 80% (**14**); (**e**) [(PPh_3_)_4_Pd], PPh_3_, pyrrolidine, MeCN, 25 °C, 3 days, yield: 96%.

Ursolic acid has also been modified before coupling with the DOTA precursors (Scheme 2). Derivatization started with the attachment of a spacer moiety using oxalyl chloride and 1-(2-aminoethyl)piperazine in dry dichloromethane affording compound **10**. The terminal amino moiety was further substituted with chloroacetyl chloride in dry dichloromethane to furnish the ursolic acid precursor **11** in excellent yield. Linkage of both precursors was performed in dry acetonitrile in the presence of potassium carbonate and potassium iodide yielding UA-DOTA conjugates **12**–**14**. Allylic esters of the acetate groups were removed by treating compound **14** with [(PPh_3_)_4_Pd], triphenylphosphane and pyrrolidine in acetonitrile at 25 °C for 3 days; this procedure gave **15** in almost quantitative yield. Purification of **15** was performed by reversed phase chromatography using MeOH/MeCN/TFA as eluent since the compound was difficult to eluate from normal silica phases. Furthermore, a synthetic approach for **15** starting from either **12** or **13** failed. Hydrogenation of **13** employing palladium catalysis retained the benzyl esters, and the deprotection of tert-butyl esters (as in compound **12**) using TFA/DCM resulted in a partial degradation of the triterpenoic backbone.

**Scheme 3:**
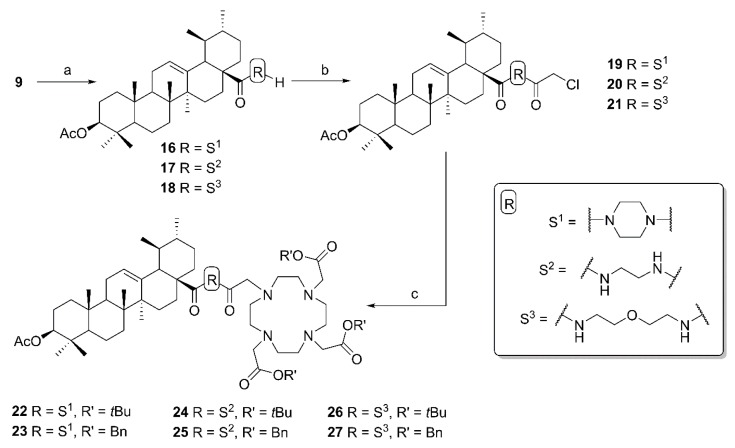
Synthesis of ursolic acid DOTA conjugates **22**–**27**: (**a**) oxalyl chloride, CH_2_Cl_2_, DMF, 0–25 °C, 1 h, then amine, CH_2_Cl_2_, 25 °C, 2 h, yield: 80% (**16** and **17**), and 78% (**18**); (**b**) chloroacetyl chloride, CH_2_Cl_2_, NEt_3_, 25 °C, 0.5–4 h, yield: 94% (**19**), and 91% (**20** and **21**); (**c**) K_2_CO_3_, KI, **6** (for **22**, **24** and **26**) or **7** (for **23**, **25** and **27**), MeCN, 25 °C, 5 days, yield: 88% (**22**), 72% (**23**), 73% (**24**), 75% (**25**), 74% (**26**), and 62% (**27**).

The synthetic approach summarized in Scheme 2 can also be applied to various other spacer units. Thus, we decided to alter the amino component. Therefore, ursolic acid was treated with piperazine, ethylene diamine and 2,2′-oxybis(ethylamine), to furnish ursolic carboxamides **16**–**18** (Scheme 3), respectively. Chloroacetyl derivatives **19**–**21** and UA-DOTA conjugates **22**–**27** were prepared analogous to Scheme 2.

**Scheme 4:**
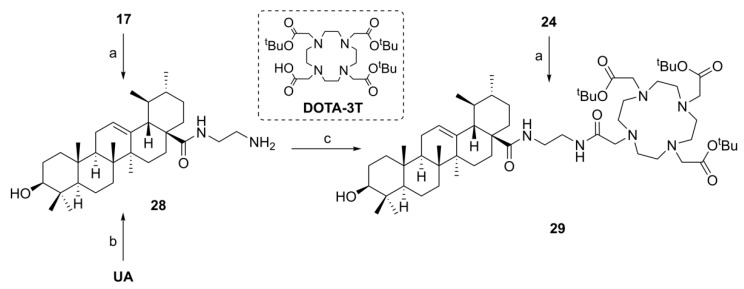
Synthesis of ursolic acid derivative **29**: (**a**) KOH, MeOH, 25 °C, 48 h (for **28**) or 24 h (for **29**), yield: 85% (**28**) or 86% (**29**); (**b**) ethylene diamine, HOBt·H_2_O, EDC·HCl, DMF, 25 °C, 24 h, yield: 46% (**c**) DOTA-3T, HOBt·H_2_O, EDC·HCl, DMF, 25 °C, 5 days, yield: 49%.

Additionally, an alternative synthetic approach was established for the preparation of the ethylene diamine-spacered UA-DOTA conjugate **29** (Scheme 4). Therefore carboxamide **28** was prepared either by deacetylation of **17** or directly from UA by amidation with ethylene diamine using EDC and HOBt in dry DMF. In the next step, DOTA-tris(*tert*-butyl ester) (DOTA-3T) was activated by preparing its HOBt ester. Adding **28** to this freshly prepared ester furnished UA-DOTA conjugate **29**. For comparison, compound **29** was also synthesized from **24** by removing the C-3 acetyloxy moiety. Due to the presence of an unprotected hydroxyl moiety at this position, compound **29** offers the possibility for a set of modifications and is therefore considered to be a good starting material for further modifications.

**Figure 2:**
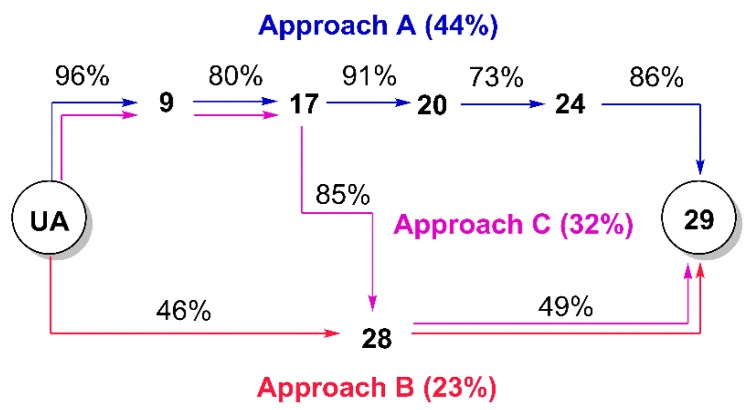
Comparison of synthetic routes **A**, **B**, and **C** for the preparation of UA-DOTA conjugate **29**.

Both synthetic approaches for the preparation of **29** (Figure 2) hold advantages but also some disadvantages. Although route A (5 steps) is significantly longer than B, but the former route gave the highest overall yield (44%). Approach B is a rather short and quick way to synthesize **29** (2 steps only), but the overall yield (23%) is barely half as high as in route A. Combining routes A and B, as shown in approach C led to compound **29** in 4 steps with an overall yield of 32%. A major difference between the approaches A and B is the availability and preparation of the DOTA precursors. Both, DOTA-tris(tert-butyl ester) and DO3A-tert-butyl ester (**6**) are available from commercial suppliers, with DOTA-3T being almost twice as expensive as **6**. Most advantageous is the one-step synthesis of **6** starting from cylcen (**2**), since **2** is commercially available for a price, being almost tenfold lower than that of **6**.

Due to cytotoxicity evaluation, the prepared UA-DOTA conjugates were screened in rhodamine B assays employing a series of human tumor cell lines and non-malignant mouse fibroblasts (NIH 3T3). Results of this investigation are summarized in Table 1.

The DO3A-tert-butyl ester (**6**) showed moderate cytotoxicity as indicated by EC_50_ values between 10 µM and 14 µM, while DO3A-benzyl ester (**7**) showed EC_50_ values lower than 5 µM. Unfortunately, most of the UA-DOTA conjugates were not soluble in solvents suitable for SRB assays. However, combining **7** with ursolic acid gave cytotoxic conjugate **13**, showing EC_50_ values below 6 µM. Removal of ester units (as in **15**) resulted in a complete loss of cytotoxicity (EC_50_ >60 µM for all tumor cells). Compounds **22** and **24**, both holding tert-butyl esters but different spacer units were also highly cytotoxic. Piperazine-spacered conjugate **22** showed the highest cytotoxicity observed in this screening for A375 tumor cells (EC_50_ = 1.5 ± 0.4 µM), while being quite selective, too (SI (NIH 3T3/A375) = 3.07, Table 2). EC_50_ values of ethylenediamine-spacered conjugate **24** were below 2.5 µM for all tumor cell lines. The highest cytotoxicity was observed for ovarian carcinoma (A2780, EC_50_ = 1.7 ± 0.1 µM). Removal of the acetyloxy moiety of **24** (as in **29**) had almost no significant impact on cytotoxicity.

**Figure 3:**
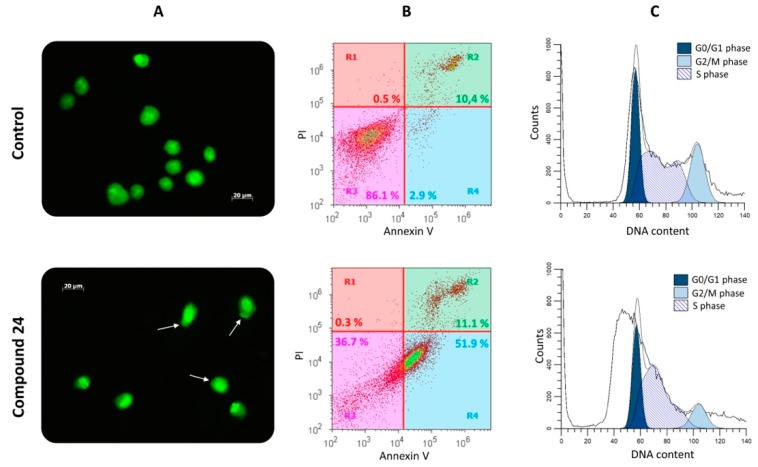
Extended cytotoxicity investigation after treatment of A375 cells with **24** (4.0 µM) for 24 h: (**A**) Fluorescence microscopic images (scale bar 20 µm), AO and PI were used; (**B**) Annexin V-FITC/PI assay. Examples of density plots determined by flow cytometry (Attune^®^ Cytometric Software v 1.2.5), R1: necrotic, R2: secondary necrotic/late stage apoptotic, R3: vital, R4: apoptotic; (**C**) Representative examples for cell cycle evaluation via ModFit LT 5.0.

Because UA-DOTA conjugates **22** and **24** were the most active compounds of this study, these compounds were selected for further cytotoxicity investigations including fluorescence microscopy, annexin V assays, and cell cycle evaluation employing melanoma cells (A375). Microscopic images of A375 cells treated with compound **24** for 24 h showed vital cells (green staining) with some of them having ruptured cell membranes (Figure 3A, white arrows). Further indications of apoptosis have been detected employing flow cytometry and annexin V-FITC/PI staining. After 24 h 63% of the tumor cells treated with **24** were annexin V-FITC-positive, and 51.9% of all cells having died by apoptosis. Additionally, the number of vital cells decreased in comparison to the control from 86.1% to 36.7 % (Figure 3B). An extra investigation of the cell cycle showed a decreased number of cells in G1/G0, G2/M, as well as in S phase. Additionally, a large population of cells has been shifted into the subG1 region (Figure 3C).

**Figure 4:**
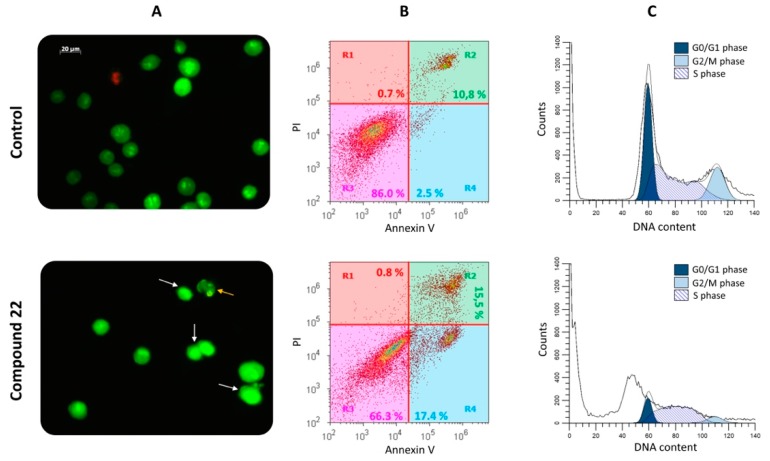
Extended cytotoxicity investigation after treatment of A375 cells with **22** (3.0 µM) for 48 h: (**A**) Fluorescence microscopic images (scale bar 20 µm), AO and PI were used; (**B**) Annexin V-FITC/PI assay. Examples of density plots determined by flow cytometry (Attune^®^ Cytometric Software vl 1.2.5), R1: necrotic, R2: secondary necrotic/late stage apoptotic, R3: vital, R4: apoptotic; (**C**) Representative examples for cell cycle evaluation via ModFit LT 5.0.

Fluorescence microscopic images and density plots of A375 cells treated with **22** for 24 h showed no significant differences in comparison to the control (Supplementary material, Figure S1). Therefore, further investigations were performed with a prolonged incubation time of 48 h. After treating A375 cells with compound **22** for 48 h, subsequent fluorescence microscopic investigations using AO/PI staining showed ruptures of the plasma membrane (Figure 4A, white arrows). Additionally, some necrotic/late stage apoptotic cells were observed, indicated by slightly orange stained nuclei (Figure 4 A, orange arrow). The density plot of A375 cells treated with **22** for 48 h showed a decreased number of vital cells (66.3%) compared to the control (86.0%), while 32.9% of the cells were considered annexin V-FITC-positive. Nearly half of them (17.4% of all cells) have died by apoptosis, and the remaining cells (15.5% of all cells) were secondary necrotic/late stage apoptotic (Figure 4 B). During extra investigations of the cell cycle, some differences compared to the control have been observed. Cells treated with **22** for 48 h showed a quite broad and flat DNA distribution. G1/G0, G2/M, and S phase were drastically reduced, while an increased population of cells with reduced DNA content has been observed in the subG1 region (Figure 4C).

## 3. Conclusions

In this study, a series of overall 11 amine-spacered UA-DOTA conjugates have been prepared starting from the natural occurring triterpenoid ursolic acid (UA). We hereby report a synthetic approach to UA-DOTA conjugates, which is applicable for several amine spacers and other triterpenoic backbones, too. Additionally, we compared three synthetic approaches for the preparation of compound **29** in terms of yield, number of steps and precursor availability. This conjugate offers the possibility for further modifications at the C-3 hydroxylic group, which is known to influence cytotoxicity. All of the prepared DOTA conjugates were screened in SRB assays showing some compounds to be of good cytotoxicity. EC_50_ values were determined to range from 17.3 µM to 1.4 µM. The most active compound of this series was a piperazinyl spacered conjugate **22** showing low EC_50_ values such as 1.5 ± 0.4 µM for A375 tumor cells and 1.9 ± 0.3 µM for A2780 tumor cells, respectively, while showing good selectivity (SI (NIH 3T3(A375) = 3.07), too. Unfortunately, the selectivity of the other screened conjugates was quite low. Additional cytotoxicity investigations such as fluorescence microscopy, annexin V assays, and cell cycle analyses were performed employing the UA-DOTA conjugates **22** and **24** to gain information about their mode of action. The results of these extended biological testing indicate **24** to induce death of A375 cancer cells by apoptosis. These results hold some starting points for further studies. Conjugate **15** and structural related compounds (holding free carboxylic acids at the DOTA unit) are currently subjects of ongoing investigations regarding their ability to form complexes with metal ions or radioactive isotopes like ^68^Ga to examine possible future uses as tracer or contrast agents in molecular imaging techniques, such as positron emission tomography (PET) [22].

## 4. Materials and Methods

### 4.1. General

NMR spectra were recorded using the Varian spectrometers Gemini 2000 or Unity 500 (Varian GmbH, Darmstadt, Germany) δ given in ppm, *J* in Hz; typical experiments: APT, H-H-COSY, HMBC, HSQC, NOESY), MS spectra were taken on a Finnigan MAT LCQ 7000 (ThermoFisher Scientific, Braunschweig, Germany) electrospray, voltage 4.1 kV, sheath gas nitrogen) instrument. The optical rotations were measured on a Perkin-Elmer polarimeter (Perkin Elmer LAS, Rodgau, Germany) or on a Jasco P-2000 polarimeter (Jasco Germany, Pfungstadt, Germany) at 20 °C; TLC was performed on NP or RP18 silica gel (Macherey-Nagel, detection with cerium molybdate or Dragendorff’s reagent). Melting points are uncorrected (*Leica* hot stage microscope, or BUCHI melting point M-565), and elemental analyses were performed on a Foss-Heraeus Vario EL (CHNS, Elementar Analysensysteme GmbH, Langenselbold, Germany) unit. IR spectra were recorded on a Perkin Elmer FT-IR spectrometer Spectrum 1000 or on a Perkin-Elmer Spectrum Two (UATR Two Unit; both instruments from Perkin Elmer LAS, Rodgau, Germany). UV-VIS spectra were taken on a Perkin-Elmer Lambda 14 spectrometer or on a Perkin-Elmer Lambda 750 S (UV/VIS/NIR) spectrometer (both instruments from Perkin Elmer LAS, Rodgau, Germany). The solvents were dried according to usual procedures. The purity of the compounds was determined by HPLC and found to be >96%.

### 4.2. Cytotoxicity

#### 4.2.1. Cell Lines and Culture Conditions

The cell lines used are human cancer cell lines: A2780 (ovarian carcinoma), HT29 (colon adenocarcinoma), MCF-7 (breast adenocarcinoma), A375 (melanoma), FaDu (pharynx squamous cell carcinoma) and non-malignant mouse fibroblasts NIH 3T3; all cell lines were obtained from the Department of Oncology (Martin-Luther-University Halle-Wittenberg). Cultures were maintained as monolayers in RPMI 1640 medium with l-glutamine (Capricorn Scientific GmbH, Ebsdorfergrund, Germany) supplemented with 10% heat inactivated fetal bovine serum (Sigma-Aldrich Chemie GmbH, Steinheim, Germany) and penicillin/streptomycin (Capricorn Scientific GmbH, Ebsdorfergrund, Germany) at 37 °C in a humidified atmosphere with 5% CO_2_.

#### 4.2.2. Cytotoxic Assay (SRB)

The cytotoxicity of the compounds was evaluated using the sulforhodamine-B (Kiton-Red S, ABCR) micro culture colorimetric assay. Cells were seeded into 96-well plates on day 0 at appropriate cell densities to prevent confluence of the cells during the period of experiment. After 24 h, the cells were treated with six different concentrations (1, 3, 7, 12, 20, and 30 µM) minimum. The final concentration of DMSO/DMF never exceeded 0.5%, which was non-toxic to the cells. After a 72-h treatment, the supernatant medium from the 96-well plates was discarded, the cells were fixed with 10% trichloroacetic acid (TCA) and allowed to rest at 4 °C. After 24 h fixation, the cells were washed in a strip washer and dyed with SRB solution (100 µL, 0.4%, in 1% acetic acid) for about 20 min. After dying, the plates were washed four times with 1% acetic acid to remove the excess of the dye and allowed to air-dry overnight. Tris base solution (200 µL, 10 mM) was added to each well and absorbance was measured at λ = 570 nm using a 96 well plate reader (Tecan Spectra, Crailsheim, Germany). The EC_50_ values were averaged from three independent experiments performed each in triplicate calculated from semi logarithmic dose response curves applying a non-linear 4P Hills-slope equation (GraphPad Prism5; variables top and bottom were set to 100 and 0, respectively).

#### 4.2.3. AO/PI Dye Exclusion Test

Morphological characteristics of cell death were analyzed employing an AO/PI assay using human cancer cell line A375. Approx. 2 × 10^5^ cells were seeded in cell culture flasks (25 cm^2^), and the cells were allowed to grow up for 24 h. After removing of the used medium, the substance loaded fresh medium was reloaded (or a blank new medium as a control). After 24 h and 48 h, the content of the flask was collected and centrifuged (1200 rpm, 4 °C), the pellet was gently suspended in phosphate-buffered saline (PBS (w/Ca^2+^ and Mg^2+^), 1 mL) and centrifuged again. The PBS was removed, and the pellet gently suspended in PBS (150 µL) again. The analysis of the cells was performed using a fluorescence microscope after having mixed the cell suspension (10 µL) with a solution of AO/PI (5 µg/mL, 10 µL).

#### 4.2.4. Annexin V-FITC/PI Assay

Approximately 2 × 10^5^ cells (A375) were seeded in cell culture flasks (25 cm^2^), and the cells were allowed to grow up for 24 h. After removing of the used medium, the substance loaded fresh medium was reloaded (or a blank fresh medium as a control). After 24 h and 48 h, the cells were harvested, centrifuged (1200 rpm, 4 °C), and washed twice with PBS (w/Ca^2+^ and Mg^2+^, 1 mL). The cells were counted and approximately 1∙10^6^ cells were washed with Annexin V binding buffer (BioLegend^®^, San Diego, USA) and treated with propidium iodide solution (3 µL, 1 mg/mL) and Annexin V-FITC (5 µL, BioLegend^®^, San Diego, CA, USA) for 15 min in the dark at room temperature. After adding Annexin V binding buffer (400 µL) the suspension was analyzed using Attune^®^ FACS machine. After gating for living cells, the data from detectors BL-1A and BL-3A were collected (20,000 events) in technical triplicates. The assay was performed in duplicates; cell distribution was calculated using Attune^®^ Software (ThermoFisher Scientific, Braunschweig, Germany).

#### 4.2.5. Cell Cycle Investigations

Approximately 2 × 10^5^ cells (A375) were seeded in cell culture flasks (25 cm^2^), and the cells were allowed to grow up for 24 h. After removing of the used medium, the substance loaded fresh medium was reloaded (or a blank fresh medium as a control). After 24 h or 48 h, respectively, only the adherent cells were harvested, centrifuged (1200 rpm, 4 °C), and washed twice with PBS ((*w*/*w*), 1 mL). The cells were counted and approximately 1 × 10^6^ cells were fixed with ethanol (70%, 4 °C, 24 h). After centrifugation (4500 rpm, 4 °C) the cells were washed with PBS ((*w*/*w*), 1 mL) and centrifuged. The pellet was resuspended in 1 mL RNAse A containing PI buffer (100µL RNAse (100 mg/mL), 15 µL PI solution (1 mg/mL)) and after incubating for 30 min at room temperature in the dark, cells were analyzed using the Attune^®^ FACS machine; collecting data from the BL-2A channel. Doublet cells were excluded from the measurements by plotting BL-2A against BL-2H. For each cell cycle distribution 20,000 events were collected in technical triplicates, each sample was measured in duplicates. Cell cycle distribution was calculated using ModFitLT™ (Verity Software House, Topsham, ME, USA).

### 4.3. Syntheses

#### 4.3.1. General

Ursolic acid (**1**) was obtained from betulinines (Stříbrná Skalice, Czech Republic). 1,4,7,10-Tetraazacyclododecane (cyclen, **2**) was bought from abcr GmbH (Karlsruhe, Germany) in 95% purity. Benzyl bromoacetate (96%) and tert-Butyl bromoacetate (98%) were both purchased from Sigma-Aldrich Chemie GmbH (Steinheim, Germany). DOTA-tris(*tert*-butyl ester) was obtained from TCI Deutschland GmbH (Eschborn, Germany) in 97% purity. Ursolic acid derivatives **10**, **16**–**18** and **28** have been synthesized as previously reported [20,21]. Experimental procedures and full analytical data of these compounds can be found in the supplementary material [23,24]. 

#### 4.3.2. General Procedure A for the Synthesis of DOTA Precursors (**6**–**8**)

To a suspension of cyclen (5.81 mmol) and sodium bicarbonate (17.43 mmol) in dry acetonitrile (100 mL), a solution of the respective bromoacetate (**3**–**5**, 17.43 mmol) in dry acetonitrile (10 mL) was added dropwise under argon atmosphere. The mixture was stirred for 48 h at 25 °C. After usual aqueous work-up, the solvent was removed under reduced pressure, and the crude products were subjected to column chromatography (silica gel, chloroform/methanol mixtures) affording DOTA precursors **6**–**8** (50–68%).

#### 4.3.3. General Procedure B for the Synthesis of Carboxamides (**10**, **16**–**18**)

Compound **9** (0.5 mmol) was dissolved in dry DCM (10 mL), cooled to 0 °C and oxalyl chloride (3.2 mmol) and dry DMF (2 drops) were added. After warming to 25 °C, the mixture was stirred for 1 h. The solvent was removed under reduced pressure, re-evaporated with dry THF (4 × 15 mL), and the residue was immediately resolved in dry DCM (10 mL). This mixture was then added dropwise to a solution of the amine (3.0 mmol) in dry DCM (2 mL) and stirred at 25 °C for 2 h. After usual aqueous work-up, the solvent was removed under reduced pressure, and the crude products were subjected to column chromatography (silica gel, chloroform/methanol mixtures). Compounds **10** and **16**–**18** were each obtained as colorless solids (78–82%).

#### 4.3.4. General Procedure C for the Alkylation with Chloroacetyl Chloride (**11**, **19**–**21**)

Chloroacetyl chloride (2.20 mmol) was added dropwise to a solution of the respective carboxamide (**10**, **16**–**18**; 1.43 mmol) and triethylamine (0.71 mmol) in dry dichloromethane (75 mL). The mixture was stirred at 25 °C for 0.5–4 h. After usual aqueous work-up, the solvent was removed under reduced pressure, and the crude products were subjected to column chromatography (silica gel, chloroform/acetone mixtures). Compounds **11** and **19**–**21** were each obtained as colorless solids (91%–94%).

#### 4.3.5. General Procedure D for the Synthesis of Ursolic Acid Chelator Conjugates (**12**–**14**, **22**–**27**)

To a solution of the respective chloroacetyl derivative (**11, 19**–**21**; 0.44 mmol) and freshly grounded potassium carbonate (0.83 mmol) in dry acetonitrile (15 mL) was added potassium iodide (0.35 mmol) and the respective DOTA precursor (**6**–**8,** 0.41 mmol in 5 mL dry acetonitrile). The mixture was stirred for 2–5 days at 25 °C. After completion of the reaction (as indicated by TLC) the mixture was filtered, and the solvent was removed under reduced pressure. The crude products were subjected to column chromatography (silica gel, chloroform/methanol mixtures) to afford compounds **12**–**14** and **22**–**27** (yield: 54–88%), respectively.

*Allyl bromoacetate* (**5**), To a solution of allyl alcohol (0.62 mol), bromo acetylbromide (0.1 mol) was added dropwise over a period of 30 min at 0 °C under argon atmosphere. The mixture was stirred for 1 h at 0 °C, warmed to 25 °C and stirred for another 3 h. The solvent was removed under reduced pressure and the residue was dissolved in dichloromethane. After usual aqueous work-up, the solvent was removed under reduced pressure, and the crude product was purified by vacuum distillation affording allyl bromoacetate as colorless oil (68%). ^1^H NMR (400 MHz, CDCl_3_): δ = 5.88 (*ddt*, *J* = 16.5, 11.0, 5.8 Hz, 1H, CH=CH_2_), 5.38–5.17 (*m*, 2H, CH=C*H*_2_), 4.61 (*dt*, *J* = 5.8, 1.4 Hz, 2H, C*H*_2_CH=CH_2_), 3.82 (*s*, 2H, BrC*H*_2_) ppm; ^13^C NMR (101 MHz, CDCl_3_): δ = 166.8 (C=O), 131.2 (*C*H=CH_2_), 119.0 (CH=*C*H_2_), 66.6 (*C*H_2_CH=CH_2_), 25.8 (Br*C*H_2_) ppm.

*Tri-tert-butyl 2,2′,2’’-(1,4,7,10-tetraazacyclododecane-1,4,7-triyl)triacetate* (**6**), Compound **6** was prepared from **2** according to general procedure A using *tert*-butyl bromoacetate (**3**). Column chromatography (SiO_2_, CHCl_3_/MeOH 9:1) gave **6** (yield: 50%); m.p. 180–182 °C (lit.: 181–183 °C [25]); R_f_ = 0.27 (CHCl_3_/MeOH 95:5); IR (ATR): ν = 2974*w*, 2943*w*, 2912*w*, 2853*w*, 2736*w*, 1718*s*, 1576*w*, 1466*w*, 1453*w*, 1412*w*, 1392*w*, 1368*m*, 1330*w*, 1255*m*, 1218*w*, 1147*s*, 1117*m*, 1099*m*, 1050*w*, 935*m*, 873*m*, 848*m* cm^−1^; ^1^H NMR (400 MHz, CDCl_3_): δ = 3.36 (*s*, 4H, 2 × CH_2_ (acetate)), 3.28 (*s*, 2H, CH_2_ (acetate)), 3.12–3.06 (*m*, 4H, 2 × CH_2_ (cyclen)), 2.95–2.84 (*m*, 12H, 6 × CH_2_ (cyclen)), 1.45 (*s*, 18H, 6 × CH_3_ (*t*-butyl)), 1.45 (*s*, 9H, 3 × CH_3_ (*t*-butyl)) ppm; ^13^C NMR (101 MHz, CDCl_3_): δ = 170.6 (2 × CO, acetate), 169.8 (CO, acetate), 81.9 (C_q_, *t*-butyl), 81.8 (2 × C_q_, *t*-butyl), 58.4 (2 × CH_2_, acetate), 51.5 (2 × CH_2_, cyclen), 51.4 (2 × CH_2_, cyclen), 49.4 (2 × CH_2_, cyclen), 49.0 (CH_2_, acetate), 47.7 (2 × CH_2_, cyclen), 28.4 (3 × CH_3_, *t*-butyl), 28.3 (6 × CH_3_, *t*-butyl) ppm; MS (ESI, MeOH): *m*/*z* = 515.3 (100%, [M + H]^+^), 537.3 (10%, [M + Na]^+^); analysis calcd for C_26_H_50_N_4_O_6_ (514.71): C 60.67, H 9.79, N 10.89; found: C 60.51, H 9.98, N 10.67.

*Tribenzyl 2,2’,2’’-(1,4,7,10-tetraazacyclododecane-1,4,7-triyl)triacetate* (**7**). Compound **7** was prepared from **2** according to general procedure A using benzyl bromoacetate (**4**). Column chromatography (SiO_2_, CHCl_3_/MeOH 9:1) gave **7** (yield: 68%); R_f_ = 0.32 (CHCl_3_/MeOH 95:5); IR (KBr): ν = 2948*w*, 2857*w*, 2738*w*, 1732*s*, 1586*w*, 1498*w*, 1455*m*, 1418*w*, 1381*w*, 1314*w*, 1169*s*, 1096*m*, 1049*m*, 994*m*, 739*s*, 697*s* cm^−1^; UV-Vis (CHCl_3_): λ_max_ (logε) = 251 nm (2.87), 258 nm (2.86), 263 nm (2.76); ^1^H NMR (400 MHz, CDCl_3_): δ = 7.40–7.29 (*m*, 15H, 15 × CH (Bn)), 5.13 (*s*, 4H, 2 × CH_2_ (Bn)), 5.13 (*s*, 2H, CH_2_ (Bn)), 3.48 (*s*, 4H, 2 × CH_2_ (acetate)), 3.41 (*s*, 2H, CH_2_ (acetate)), 3.12–3.05 (*m*, 4H, 2 × CH_2_ (cyclen)), 2.93–2.79 (*m*, 12H, 6 × CH_2_ (cyclen)) ppm; ^13^C NMR (101 MHz, CDCl_3_): δ = 171.1 (2 × CO, acetate), 170.3 (CO, acetate), 135.5 (C_i_, Bn), 128.8 (CH, Bn), 128.8 (CH, Bn), 128.7 (CH, Bn), 128.7 (CH, Bn), 128.6 (CH, Bn), 66.8 (CH_2_, Bn), 57.4 (2 × CH_2_, acetate), 51.9 (2 × CH_2_, cyclen), 51.7 (2 × CH_2_, cyclen), 49.6 (2 × CH_2_, cyclen), 48.8 (CH_2_, acetate), 47.5 (2 × CH_2_, cyclen) ppm; MS (ESI, MeOH): *m*/*z* = 309.0 (10%, [M + 2H]^2+^), 617.4 (100%, [M + H]^+^), 639.3 (10%, [M + Na]^+^); analysis calcd for C_35_H_44_N_4_O_6_ (616.76): C 68.16, H 7.19, N 9.08; found: C 67.84, H 7.39, N 8.81.

*Triallyl 2,2’,2’’-(1,4,7,10-tetraazacyclododecane-1,4,7-triyl)triacetate* (**8**), Compound **8** was prepared from **2** according to general procedure A using allyl bromoacetate (**5**). Column chromatography (SiO_2_, CHCl_3_/MeOH 95:5) gave **8** (yield: 63%); R_f_ = 0.27 (CHCl_3_/MeOH 95:5); IR (KBr): ν = 2945*w*, 2858*w*, 2743*w*, 1731*s*, 1673*w*, 1648*w*, 1455*w*, 1420*w*, 1364*w*, 1314*w*, 1179*s*, 1095*m*, 985*s*, 928*s* cm^−1^; ^1^H NMR (400 MHz, CDCl_3_): δ = 5.93–5.81 (*m*, 3H, 3 × CH (allyl)), 5.32–5.20 (*m*, 6H, 3 × CH_2_ (allyl)), 4.60–4.53 (*m*, 6H, 3 × CH_2_ (allyl)), 3.49 (*s*, 4H, 2 × CH_2_ (acetate)), 3.41 (*s*, 2H, CH_2_ (acetate)), 3.12–3.06 (*m*, 4H, 2 × CH_2_ (cyclen)), 2.96–2.81 (*m*, 12H, 6 × CH_2_ (cyclen)) ppm; ^13^C NMR (101 MHz, CDCl_3_): *δ* = 170.8 (2 × CO, acetate), 170.0 (CO, acetate), 131.7 (2 × CH, allyl), 131.7 (CH, allyl), 119.2 (CH_2_, allyl), 119.0 (2 × CH_2_, allyl), 65.5 (2 × CH_2_, allyl), 65.4 (CH_2_, allyl), 57.3 (2 × CH_2_, acetate), 51.7 (2 × CH_2_, cyclen), 51.6 (2 × CH_2_, cyclen), 49.4 (2 × CH_2_, cyclen), 48.5 (CH_2_, acetate), 47.4 (CH_2_, cyclen) ppm; MS (ESI, MeOH): *m*/*z* = 234.1 (18%, [M + 2H]^2+^), 467.3 (100%, [M + H]^+^), 489.3 (10%, [M + Na]^+^); analysis calcd for C_23_H_38_N_4_O_6_ (466.6): C 59.21, H 8.21, N 12.01; found: C 59.03, H 8.44, N 11.78.

*(3β) 3-Acetyloxy-urs-12-en-28-oic acid* (**9**), Compound **1** was prepared from ursolic acid according to the procedure given in the literature [26]. Yield: 96%; m.p. 287–290 °C (lit.: 289–290 °C [27]).

*(3β) N-(2-(4-(2-Chloroacetyl)piperazin-1-yl)ethyl)-3-acetyloxy-urs-12-en-28-amide* (**11**). Compound **11** was synthesized from **10** according to general procedure C. Column chromatography (SiO_2_, CHCl_3_/acetone 4:1) furnished compound **11** (91%); m.p. 124–129 °C; [α]_D_ = +32.3° (*c* 0.320, CHCl_3_); R_f_ = 0.38 (CHCl_3_/acetone 4:1); IR (KBr): ν = 3423*s*, 2947*s*, 1734*m*, 1654*s*, 1522*m*, 1458*m*, 1370*m*, 1247*s*, 1150*w*, 1027*m* cm^−1^; ^1^H NMR (400 MHz, CDCl_3_): *δ* = 6.32 (*t*, *J* = 5.0 Hz, 1H, N*H*), 5.28 (*t*, *J* = 3.6 Hz, 1H, 12-H), 4.49 (*dd*, *J* = 10.4, 5.4 Hz, 1H, 3-H), 4.06 (*s*, 2H, 36-H), 3.72–3.47 (*m*, 4H, 34-H, 34′-H), 3.47–3.36 (*m*, 1H, 31-H_a_), 3.25–3.15 (*m*, 1H, 31-H_b_), 2.58–2.39 (*m*, 6H, 33-H, 32-H, 33′-H), 2.04 (*s*, 3H, Ac), 2.02–1.80 (*m*, 5H, 11-H_a_, 11-H_b_, 16-H_a_, 22-H_a_, 18-H), 1.79–1.70 (*m*, 1H, 16-H_b_), 1.69–1.21 (*m*, 13H, 15-H_a_, 1-H_a_, 2-H_a_, 2-H_b_, 9-H, 6-H_a_, 21-H_a_, 7-H_a_, 22-H_b_, 19-H, 6-H_b_, 21-H_b_, 7-H_b_), 1.09 (*s*, 3H, 27-H), 1.08–1.01 (*m*, 2H, 1-H_b_, 15-H_b_), 0.96–0.94 (*m*, 4H, 20-H, 30-H), 0.93 (*s*, 3H, 25-H), 0.89–0.86 (*m*, 3H, 29-H), 0.86 (*s*, 3H, 23-H), 0.85 (*s*, 3H, 24-H), 0.84–0.79 (*m*, 1H, 5-H), 0.78 (*s*, 3H, 26-H) ppm; ^13^C NMR (101 MHz, CDCl_3_): δ = 178.1 (C-28), 171.1 (Ac), 165.2 (C-35), 140.0 (C-13), 125.3 (C-12), 80.9 (C-3), 56.7 (C-32), 55.4 (C-5), 54.2 (C-18), 53.0 (C-33), 52.5 (C-33′), 48.0 (C-17), 47.6 (C-9), 46.5 (C-34), 42.6 (C-14), 42.4 (C-34′), 40.9 (C-36), 39.9 (C-19), 39.7 (C-8), 39.3 (C-20) 38.4 (C-1), 37.8 (C-4), 37.5 (C-22), 37.0 (C-10), 35.9 (C-31), 32.8 (C-7), 31.0 (C-21), 28.2 (C-23), 28.0 (C-15), 25.0 (C-16), 23.6 (C-2), 23.6 (C-11), 23.4 (C-27), 21.4 (Ac), 21.3 (C-30), 18.3 (C-6), 17.5 (C-29), 17.1 (C-26), 16.9 (C-24), 15.8 (C-25) ppm; MS (ESI, MeOH): **m*/*z** = 686.5 (100%, [M + H]^+^); analysis calcd for C_40_H_64_ClN_3_O_4_ (686.42): C 69.99, H 9.40, N 6.12; found: C 69.70, H 9.63, N 6.02.

*Tris-t-butyl 2’,2’’-[10-[2-[4-[2-(3β-acetyloxy-urs-12-en-28-oylamino)ethyl]piperazin-1-yl]-2-oxoethyl]-1,4,7,10-tetraazacyclododecane-1,4,7-triyl]triacetate* (**12**), Compound **12** was synthesized from **6** and **11** according to general procedure D. Column chromatography (SiO_2_, CHCl_3_/MeOH 9:1) furnished compound **12** (54%). m.p. 247–250 °C (decomp.); [α]_D_ = +18.9° (c 0.345, CHCl_3_); R_f_ = 0.30 (CHCl_3_/MeOH 9:1); IR (KBr): ν = 2931*m*, 1727*s*, 1644*s*, 1529*w*, 1455*m*, 1425*w*, 1368*s*, 1306*m*, 1228*s*, 1159*s*, 1105*s*, 1005*m*, 755*m* cm^−1^; ^1^H NMR (400 MHz, CDCl_3_): δ = 6.38 (*s*, 1H, NH), 5.26 (*t*, *J* = 3.4 Hz, 1H, 12-H), 4.46 (*dd*, *J* = 10.5, 5.2 Hz, 1H, 3-H), 3.92–2.04 (*m*, 36H, 34-H, 34′-H, 36-H, 3 × CH_2_ (acetate), 31-H_a_, 31-H_b_, 8 × CH_2_ (cyclen), 32-H, 33-H, 33′-H), 2.01 (*s*, 3H, Ac), 2.00–1.68 (*m*, 4H, 16-H_a_, 11-H_a_, 11-H_b_, 18-H), 1.69–1.14 (*m*, 14H, 16-H_b_, 22-H_b_, 15-H_a_, 1-H_a_, 2-H_a_, 2-H_b_, 9-H, 6-H_a_, 21-H_a_, 7-H_a_, 19-H, 6-H_b_, 21-H_b_, 7-H_b_), 1.42 (*s*, 27H, 9 × CH_3_ (t-Butyl)), 1.05 (*s*, 3H, 27-H), 1.11–0.95 (*m*, 3H, 1-H_b_, 15-H_b_, 20-H), 0.91 (*d*, *J* = 6.1 Hz, 3H, 30-H), 0.90 (*s*, 3H, 25-H), 0.85 (*d*, *J* = 6.5 Hz, 3H, 29-H), 0.83 (*s*, 3H, 23-H), 0.82 (*s*, 3H, 24-H), 0.80–0.75 (*m*, 1H, 5-H), 0.74 (*s*, 3H, 26-H) ppm; ^13^C NMR (100 MHz, CDCl_3_): δ = 178.0 (C-28), 172.8 (CO, acetate), 171.0 (Ac), 169.8 (C-35), 139.8 (C-13), 125.3 (C-12), 81.9 (C_q_, *t*-Butyl), 81.7 (2 × C_q_, *t*-Butyl), 80.9 (C-3), 56.8 (C-32), 55.8 (3 × CH_2_, acetate), 55.3 (C-5), 55.2 (C-36), 53.9 (C-18), 53.4 (8 × CH_2_, cyclen), 52.2 (C-33, C-33′), 47.8 (C-17), 47.5 (C-9), 44.3 (C-34, C-34′), 42.5 (C-14), 39.8 (C-19), 39.7 (C-8), 39.1 (C-20), 38.4 (C-1), 37.8 (C-4), 37.4 (C-22), 36.9 (C-10), 35.7 (C-31), 32.8 (C-7), 31.0 (C-21), 28.2 (C-23), 28.0 (9 × CH_3_, t-Butyl), 27.9 (C-15), 24.8 (C-16), 23.6 (C-2), 23.5 (C-11), 23.4 (C-27), 21.4 (Ac), 21.3 (C-30), 18.3 (C-6), 17.4 (C-29), 17.1 (C-26), 16.8 (C-24), 15.7 (C-25) ppm; MS (ESI, MeOH): *m*/*z* = 593.9 (100%, [M + Na + H]^+^), 1186.7 (95%, [M + Na]^+^); analysis calcd for C_66_H_113_N_7_O_10_ (1164.67): C 68.06, H 9.78, N 8.42; found: C 67.75, H 9.97, N 8.51.

*Tribenzyl 2,2’,2’’-[10-[2-[4-[2-(3β-acetyloxy-urs-12-en-28-oylamino)ethyl]piperazin-1-yl]-2-oxoethyl]-1,4,7,10-tetraazacyclododecane-1,4,7-triyl]triacetate* (**13**). Compound **13** was synthesized from **7** and **11** according to general procedure D. Column chromatography (SiO_2_, CHCl_3_/MeOH 95:5) furnished compound **13** (82%). m.p. 142–146 °C; [α]_D_ = +14.5° (c 0.300, CHCl_3_); R_f_ = 0.50 (CHCl_3_/MeOH 9:1); IR (KBr): ν = 3440*s*, 2947*m*, 1734*s*, 1641*s*, 1456*m*, 1371*m*, 1310*w*, 1247*m*, 1197*s*, 1105*m*, 1006*w*, 750*m* cm^−1^; UV-Vis (CHCl_3_): λ_max_ (logε) = 257 nm (3.99); ^1^H NMR (400 MHz, CDCl_3_): δ = 7.36–7.24 (*m*, 15H, CH_Ar_), 6.36–6.28 (*m*, 1H, NH), 5.27 (*t*, *J* = 3.7 Hz, 1H, 12-H), 5.21–5.13 (*m*, 4H, 2 × CH_2_Bn), 5.12–5.05 (*m*, 2H, CH_2_Bn), 4.47 (*dd*, *J* = 10.7, 5.1 Hz, 1H, 3-H), 3.72–2.81 (*m*, 12H, 34-H, 34′-H, 36-H, 3 × CH_2_ (acetate)), 3.40–3.30 (*m*, 1H, 31-H_a_), 3.22–3.14 (*m*, 1H, 31-H_b_), 2.46–2.33 (*m*, 6H, 32-H, 33-H, 33′-H), 2.81–2.06 (*m*, 16H, 8 × CH_2_ (cyclen)), 2.03 (*s*, 3H, Ac), 2.00–1.68 (*m*, 6H, 16-H_a_, 11-H_a_, 11-H_b_, 18-H, 22-H_a_, 16-H_b_), 1.68–1.20 (*m*, 13H, 15-H_a_, 1-H_a_, 2-H_a_, 2-H_b_, 9-H, 6-H_a_, 21-H_a_, 7-H_a_, 22-H_b_, 19-H, 6-H_b_, 21-H_b_, 7-H_b_), 1.06 (*s*, 3H, 27-H), 1.05–0.94 (*m*, 3H, 1-H_b_, 15-H_b_, 20-H), 0.93 (*brs*, 3H, 30-H), 0.91 (*s*, 3H, 25-H), 0.85 (*d*, *J* = 5.7 Hz, 3H, 29-H), 0.85 (*s*, 3H, 23-H), 0.83 (*s*, 3H, 24-H), 0.82–0.77 (*m*, 1H, 5-H), 0.76 (*s*, 3H, 26-H) ppm; ^13^C NMR (101 MHz, CDCl_3_): δ = 177.9 (C-28), 173.5 (CO, acetate), 170.9 (Ac), 170.0 (C-35), 139.7 (C-13), 135.4 (C_Ar_), 135.3 (C_Ar_), 135.2 (C_Ar_), 128.7 (CH_Ar_), 128.6 (CH_Ar_), 128.5 (CH_Ar_), 128.3 (CH_Ar_), 128.3 (CH_Ar_), 128.2 (CH_Ar_), 125.3 (CH_Ar_), 80.8 (C-3), 67.0 (CH_2_, Bn), 66.8 (CH_2_, Bn), 56.7 (C-32), 55.4 (C-36), 55.3 (CH_2_, acetate), 55.2 (C-5), 53.9 (C-18), 53.4 (CH_2_, cyclen), 52.7 (C-33, C-33′), 47.7 (C-17), 47.4 (C-9), 42.4 (C-14), 39.7 (C-19), 39.5 (C-8), 39.0 (C-20), 38.3 (C-1), 37.6 (C-4), 37.3 (C-22), 36.8 (C-10), 35.8 (C-31), 32.7 (C-7), 30.9 (C-21), 28.0 (C-23), 27.8 (C-15), 24.8 (C-16), 23.5 (C-2), 23.4 (C-11), 23.2 (C-27), 21.3 (Ac), 21.2 (C-30), 18.1 (C-6), 17.3 (C-29), 17.0 (C-26), 16.7 (C-24), 15.6 (C-25) ppm; MS (ESI, MeOH): *m*/*z* = 634 (20%, [M + 2H]^2+^), 645 (100%, [M + H + Na]^2+^), 1289 (62%, [M + Na]^+^); analysis calcd for C_75_H_107_N_7_O_10_ (1266.72): C 71.11, H 8.51, N 7.74; found: C 70.73, H 8.70, N 7.49.

*Triallyl 2,2’,2’’-[10-[2-[4-[2-(3β-acetyloxy-urs-12-en-28-oylamino)ethyl]piperazin-1-yl]-2-oxoethyl]-1,4,7,10-tetraazacyclododecane-1,4,7-triyl]triacetate* (**14**). Compound **14** was synthesized from **8** and **11** according to general procedure D. Column chromatography (SiO_2_, CHCl_3_/MeOH 95:5) furnished compound **14** (80%); m.p. 159–163 °C (decomp.); [α]_D_ = +14.8° (c 0.310, CHCl_3_); R_f_ = 0.35 (SiO_2_, CHCl3/MeOH 9:1); IR (KBr): ν = 3342*s*, 2946*m*, 2852*w*, 1734*s*, 1642*s*, 1522*w*, 1456*m*, 1386*w*, 1310*w*, 1246*m*, 1202*m*, 1106*m*, 1026*w* cm^−1^; ^1^H NMR (400 MHz, CDCl_3_): δ = 6.35 (*s*, 1H, NH), 5.97 – 5.83 (*m*, 3H, 3 × CH (allyl)), 5.34–5.19 (*m*, 7H, 12-H, 3 × CH_2_ (allyl)), 4.67–4.55 (*m*, 6H, 3 × CH_2_ (allyl)), 4.47 (*dd*, *J* = 10.6, 5.3 Hz, 1H, 3-H), 3.72–2.97 (*m*, 14H, 34-H, 34′-H, 36-H, 3 × CH_2_ (acetate), 31-H_a_, 31-H_b_), 2.97–2.14 (*m*, 22H, 8 × CH_2_ (cyclen), 32-H, 33-H, 33′-H), 2.03 (*s*, 3H, Ac), 2.01–1.68 (*m*, 6H, 16-H_a_, 11-H_a_, 11-H_b_, 18-H, 22-H_a_, 16-H_b_), 1.68–1.19 (*m*, 13H, 15-H_a_, 1-H_a_, 2-H_a_, 2-H_b_, 9-H, 6-H_a_, 21-H_a_, 7-H_a_, 22-H_b_, 19-H, 6-H_b_, 21-H_b_, 7-H_b_), 1.07 (*s*, 3H, 27-H), 1.06–0.94 (*m*, 3H, 1-H_b_, 15-H_b_, 20-H), 0.94 (*d*, *J* = 6.5 Hz, 3H, 30-H), 0.92 (*s*, 3H, 25-H), 0.87 (*d*, *J* = 6.5 Hz, 3H, 29-H), 0.85 (*s*, 3H, 23-H), 0.83 (*s*, 3H, 24-H), 0.82–0.78 (*m*, 1H, 5-H), 0.76 (*s*, 3H, 26-H) ppm; ^13^C NMR (101 MHz, CDCl_3_): δ = 178.0 (C-28), 173.4 (2 x CO, acetate), 173.3 (CO, acetate), 171.1 (Ac), 170.0 (C-35), 139.8 (C-13), 131.9 (2 × CH, allyl), 131.7 (CH, allyl), 125.4 (C-12), 119.0 (CH_2_, allyl), 118.8 (2 × CH_2_, allyl), 80.9 (C-3), 66.0 (CH_2_, allyl), 65.9 (2 × CH_2_, allyl), 56.8 (C-32), 55.4 (C-36), 55.3 (C-5), 55.2 (3 × CH_2_, acetate), 53.9 (C-18), 53.6 (8 × CH_2_, cyclen), 52.7 (C-33, C-33′), 47.9 (C-17), 47.5 (C-9), 45.0 (C-34, C-34′), 42.6 (C-24), 39.8 (C-19), 39.7 (C-8), 39.1 (C-20), 38.4 (C-1), 37.8 (C-4), 37.4 (C-22), 37.0 (C-10), 35.9 (C-31), 32.8 (C-7), 31.0 (C-21), 28.2 (C-23), 27.9 (C-15), 24.9 (C-16), 23.6 (C-2), 23.6 (C-11), 23.3 (C-27), 21.4 (Ac), 21.3 (C-30), 18.3 (C-6), 17.4 (C-29), 17.1 (C-26), 16.8 (C-24), 15.7 (C-25) ppm; MS (ESI, MeOH): *m*/*z* = 569.8 (100%, [M + Na + H]^+^), 1138.8 (52%, [M + Na]^+^); analysis calcd for C_63_H_101_N_7_O_10_ (1116.54): C 67.77, H 9.12, N 8.78; found: C 67.50, H 9.37, N 8.43.

*2,2’,2’’-[10-[2-[4-[2-(3β-Acetyloxy-urs-12-en-28-oylamino)ethyl]piperazin-1-yl]-2-oxoethyl]-1,4,7,10-tetraazacyclododecane-1,4,7-triyl]triacetic acid* (**15**). Triphenylphosphane (0.038 mmol), [(PPh_3_)_4_Pd] (0.013 mmol) and pyrrolidine (0.290 mmol) were added to a solution of compound **14** (0.128 mmol) in acetonitrile (4 mL), and the mixture was stirred for 6 days at 25 °C. After filtration, the solvent was removed under reduced pressure, and the crude product was subjected to column chromatography (RP18, MeCN/MeOH/TFA 60:40:0.1) affording compound **15** as colorless solid (96%); m.p. 206–210 °C (decomp.); [α]_D_ = +17.1° (c 0.315, MeOH); R_f_ = 0.35 (RP18, ACN/TFA 100:1); IR (ATR): ν = 2925*w*, 1634*s*, 1371*m*, 1245*s*, 1199*s*, 1127*s*, 1026*m*, 829*m*, 800*m*, 719*m* cm^−1^; ^1^H NMR (400 MHz, CD_3_OD): δ = 5.35 (*t*, *J* = 3.6 Hz, 1H, 12-H), 4.47 (*dd*, *J* = 11.0, 5.3 Hz, 1H, 3-H), 3.72–2.93 (*m*, 14H, 34-H, 34‘-H, 36-H, 31-H_a_, 31-H_b_, 3 × CH_2_ (acetate)), 2.91–2.20 (*m*, 22H, 32-H, 33-H, 33‘-H, 8 × CH_2_ (cyclen)), 2.09–2.06 (*m*, 1H, 18-H), 2.03 (*s*, 3H, Ac), 2.02–1.93 (*m*, 3H, 11-H_a_, 11-H_b_, 16-H_a_), 1.84–1.23 (*m*, 15H, 15-H_a_, 22-H_a_, 1-H_a_, 16-H_b_, 2-H_a_, 2-H_b_, 9-H, 7-H_a_, 6-H_a_, 21-H_a_, 22-H_b_, 19-H, 6-H_b_, 21-H_b_, 7-H_b_), 1.15 (*s*, 3H, 27-H), 1.12–0.96 (*m*, 3H, 15-H_b_, 1-H_b_, 20-H), 0.99 (*s*, 3H, 25-H), 0.97 (*brs*, 3H, 30-H), 0.92 (*d*, *J* = 6.4 Hz, 3H, 29-H), 0.89 (*s*, 3H, 24-H), 0.88 (*s*, 3H, 23-H), 0.87–0.84 (*m*, 1H, 5-H), 0.83 (*s*, 3H,26-H) ppm; ^13^C NMR (101 MHz, CD_3_OD): δ = 180.1 (C-28), 172.8 (Ac), 172.5 (CO, acetate), 172.1 (2 × CO, acetate), 171.2 (C-35), 140.2 (C-13), 127.0 (C-12), 82.4 (C-3), 59.8 (CH_2_, acetate), 59.7 (CH_2_, acetate), 59.1 (CH_2_, acetate), 57.7 (C-32), 56.7 (C-5), 55.3 (C-36), 54.4 (C-18), 54.2 (8 × CH_2_, cyclen), 53.8 (C-33, C-33‘), 49.0 (C-17), 48.8 (C-9), 46.2 (C-34. C-34‘), 43.4 (C-14), 40.9 (C-8), 40.9 (C-19), 40.3 (C-20), 39.4 (C-1), 38.7 (C-4), 38.7 (C-22), 38.1 (C-10), 37.3 (C-31), 34.0 (C-7), 31.9 (C-21), 29.0 (C-15), 28.6 (C-23), 25.4 (C-16), 24.6 (C-2), 24.5 (C-11), 24.0 (C-27), 21.6 (C-30), 21.1 (Ac), 19.3 (C-6), 18.0 (C-26), 17.8 (C-29), 17.2 (C-24), 16.1 (C-25) ppm; MS (ESI, MeOH, positive ion mode): *m*/*z* = 1018.6 (27%, [M + Na]^+^), 1034.7 (100%, [M + K]^+^); MS (ESI, MeOH, negative ion mode): *m*/*z* = 1017.7 (13%, [M − 2H + Na]^–^), 1032.6 (100%, [M – 2H + K]^–^); analysis calcd for C_54_H_89_N_7_O_10_ (996.35): C 65.10, H 9.00, N 9.84; found: C 64.82, H 9.21, N 9.61.

*1-(3β-Acetyloxy-urs-12-en-28-oyl)-4-(2-chloroacetyl) piperazine* (**19**). Compound **19** has been synthesized from **16** according to general procedure C. Column chromatography (SiO_2_, CHCl_3_/acetone/hexanes 95:5:20) furnished compound **19** (94%); m.p. 155–158 °C; [α]_D_ = +34.3° (*c* 0.370, CHCl_3_); R_f_ = 0.66 (CHCl_3_/acetone 9:1); IR (ATR): ν = 2924*m*, 2871*w*, 1731*m*, 1658*s*, 1455*m*, 1392*m*, 1370*m*, 1243*s*, 1200*m*, 1145*m*, 1025*m*, 985*m*, 752*m* cm^−1^; ^1^H NMR (400 MHz, CDCl_3_): *δ* = 5.21 (*t*, *J* = 3.6 Hz, 1H, 12-H), 4.52–4.45 (*m*, 1H, 3-H), 4.06 (*s*, 2H, 34-H), 3.73–3.46 (*m*, 8H, 31-H_,_ 31′-H, 32-H, 32′-H), 2.41 (*d*, *J* = 11.6 Hz, 1H, 18-H), 2.24–2.11 (*m*, 1H, 16-H_a_), 2.03 (*s*, 3H, Ac), 1.91 (*dd*, *J* = 8.9, 3.6 Hz, 2H, 11-H_a_, 11-H_b_), 1.80–1.24 (*m*, 15H, 15-H_a_, 16-H_b_, 22-H_a_, 1-H_a_, 2-H_a_, 2-H_b_, 22-H_b_, 9-H, 6-H_a_, 21-H_a_, 7-H_a_, 19-H, 6-H_b_, 21-H_b_, 7-H_b_), 1.07 (*s*, 3H, 27-H), 1.12–0.98 (*m*, 3H, 1-H_b_, 15-H_b_, 20-H), 0.95 (*d*, *J* = 6.2 Hz, 3H, 30-H), 0.93 (*s*, 3H, 25-H), 0.88 (*d*, *J* = 6.4 Hz, 3H, 29-H), 0.85 (*s*, 3H, 23-H), 0.84 (*s*, 3H, 24-H), 0.84–0.77 (*m*, 1H, 5-H), 0.73 (*s*, 3H, 26-H) ppm; ^13^C NMR (101 MHz, CDCl_3_): δ = 175.8 (C-28), 171.1 (Ac), 165.5 (C-33), 138.6 (C-13), 125.5 (C-12), 81.0 (C-3), 55.5 (C-5), 55.1 (C-18), 48.8 (C-17), 47.7 (C-9), 46.3 (C-31), 45.5 (C-31′), 45.1 (C-32), 42.3 (C-32′, C-14), 40.9 (C-34), 39.6 (C-19), 39.6 (C-8), 38.9 (C-20), 38.4 (C-1), 37.8 (C-4), 37.1 (C-10), 34.6 (C-22), 33.1 (C-7), 30.6 (C-21), 28.3 (C-15), 28.2 (C-23), 23.9 (C-27), 23.7 (C-2, C-16), 23.4 (C-11), 21.4 (Ac), 21.4 (C-30), 18.3 (C-6), 17.6 (C-29), 17.0 (C-26), 16.9 (C-24), 15.6 (C-25) ppm; MS (ESI, MeOH): *m*/*z* = 643.5 (100%, [M + H]^+^), 665.4 (56%, [M + Na]^+^), 1307.3 78%, [2M + Na]^+^); analysis calcd for C_38_H_59_ClN_2_O_4_ (643.4): C 70.94, H 9.24, N 4.35; found: C 70.72, H 9.51, N 4.09.

*(3β) N-(2-(2-Chloroacetyl)aminoethyl)-3-acetyloxy-urs-12-en-28-amide* (**20**). Compound **20** was synthesized from **17** according to general procedure C. Column chromatography (SiO_2_, CHCl_3_/acetone/hexanes 95:5:20) furnished compound **20** (91%); m.p. 103–107 °C; [α]_D_ = +25.2° (*c* 0.300, CHCl_3_); R_f_ = 0.40 (CHCl_3_/acetone 9:1); IR (KBr): ν = 3422*br s*, 2948*s*, 2872*m*, 1734*s*, 1640*s*, 1532*s*, 1456*m*, 1370*m*, 1246*s*, 1148*w*, 1092*w*, 1028*m*, 756*m* cm^−1^; ^1^H NMR (400 MHz, CDCl_3_): *δ* = 7.43 (*t*, *J* = 5.1 Hz, 1H, NH), 6.28 (*t*, *J* = 5.7 Hz, 1H, NH), 5.32 (*t*, *J* = 3.6 Hz, 1H, 12-H), 4.48 (*dd*, *J* = 9.7, 6.1 Hz, 1H, 3-H), 4.00 (*s*, 2H, 34-H), 3.56–3.46 (*m*, 1H, 31-H_a_), 3.41–3.35 (*m*, 2H, 32-H), 3.26–3.18 (*m*, 1H, 31-H_b_), 2.04 (*s*, 3H, Ac), 2.03–1.80 (*m*, 5H, 16-H_a_, 11-H_a_, 11-H_b_, 18-H, 22-H_a_), 1.80–1.22 (*m*, 14H, 16-H_b_, 2-H_a_, 2-H_b_, 1-H_a_, 15-H_a_, 9-H, 6-H_a_, 21-H_a_, 7-H_a_, 22-H_b_, 19-H, 6-H_b_, 21-H_b_, 7-H_b_), 1.08 (*s*, 3H, 27-H), 1.08–0.95 (*m*, 3H, 1-H_b_, 15-H_b_, 20-H), 0.94 (*s*, 3H, 30-H), 0.93 (*s*, 3H, 25-H), 0.87 (*d*, *J* = 6.5 Hz, 3H, 29-H), 0.86 (*s*, 3H, 23-H), 0.84 (*s*, 3H, 24-H), 0.84–0.79 (*m*, 1H, 5-H), 0.75 (*s*, 3H, 26-H) ppm; ^13^C NMR (101 MHz, CDCl_3_): δ = 180.0 (C-28), 171.1 (Ac), 167.0 (C-33), 139.7 (C-13), 125.9 (C-12), 81.0 (C-3), 55.4 (C-5), 53.8 (C-18), 48.0 (C-17), 47.6 (C-9), 42.6 (C-14), 42.6 (C-34), 41.3 (C-32), 39.9 (C-19), 39.7 (C-8), 39.2 (C-31), 39.2 (C-20), 38.4 (C-1), 37.8 (C-4), 37.4 (C-22), 37.0 (C-10), 32.8 (C-7), 31.0 (C-21), 28.2 (C-23), 27.9 (C-15), 24.9 (C-16), 23.7 (C-2), 23.5 (C-11), 23.4 (C-27), 21.4 (Ac), 21.3 (C-30), 18.3 (C-6), 17.4 (C-29), 17.0 (C-26), 16.8 (C-24), 15.7 (C-25) ppm; MS (ESI, MeOH): *m*/*z* = 617.3 (48%, [M + H]^+^), 639.5 (52%, [M + Na]^+^), 1255.4 (100%, [2M + Na]^+^); analysis calcd for C_36_H_57_ClN_2_O_4_ (617.31): C 70.04, H 9.31, N 4.54; found: C 69.83, H 9.52, N 4.11.

*(3β) N-(2-(2-(2-Chloroacetyl)aminoethoxy)ethyl)-3-acetyloxy-urs-12-en-28-amide* (**21**). Compound **21** has been synthesized from **18** according to general procedure C. Column chromatography (SiO_2_, CHCl_3_/acetone/hexanes 95:5:20) furnished compound **21** (91%); m.p. 94–97 °C; [α]_D_ = +33.7° (*c* 0.335, CHCl_3_); R_f_ = 0.35 (CHCl_3_/acetone 9:1); IR (KBr): ν = 3426*br s*, 2928*m*, 2872*m*, 1734*m*, 1638*s*, 1528*m*, 1458*w*, 1386*w*, 1248*s*, 1124*w*, 1028*m* cm^−1^; ^1^H NMR (400 MHz, CDCl_3_): δ = 7.00–6.90 (*m*, 1H, NH), 6.22 (*t*, *J* = 5.0 Hz, 1H, NH), 5.30 (*t*, *J* = 3.6 Hz, 1H, 12-H), 4.49 (*dd*, *J* = 10.0, 5.7 Hz, 1H, 3-H), 4.06 (*s*, 2H, 36-H), 3.57–3.47 (*m*, 7H, 32-H, 31-H_a_, 33-H, 34-H), 3.31–3.21 (*m*, 1H, 31-H_b_), 2.04 (*s*, 3H, Ac), 2.02–1.76 (*m*, 5H, 16-H_a_, 11-H_a_, 11-H_b_, 18-H, 22-H_a_), 1.77–1.21 (*m*, 14H, 16-H_b_, 15-H_a_, 1-H_a_, 2-H_a_, 2-H_b_, 9-H, 6-H_a_, 21-H_a_, 7-H_a_, 22-H_b_, 19-H, 6-H_b_, 21-H_b_, 7-H_b_), 1.09 (*s*, 3H, 27-H), 1.08–0.95 (*m*, 3H, 1-H_b_, 15-H_b_, 20-H), 0.94 (*d*, *J* = 6.2 Hz, 3H, 30-H), 0.93 (*s*, 3H, 25-H), 0.88 (*d*, *J* = 6.2 Hz, 3H, 29-H), 0.86 (*s*, 3H, 23-H), 0.85 (*s*, 3H, 24-H), 0.84–0.80 (*m*, 1H, 5-H), 0.78 (*s*, 3H, 26-H) ppm; ^13^C NMR (101 MHz, CDCl_3_): δ = 178.4 (C-28), 171.1 (Ac), 166.1 (C-35), 139.9 (C-13), 125.6 (C-12), 80.9 (C-3), 70.0 (C-33), 69.4 (C-32), 55.4 (C-5), 54.0 (C-18), 48.0 (C-17), 47.6 (C-9), 42.8 (C-36), 42.6 (C-14), 39.9 (C-29), 39.8 (C-34), 39.7 (C-8), 39.2 (C-31), 39.2 (C-20), 38.5 (C-1), 37.8 (C-4), 37.4 (C-22), 37.0 (C-10), 32.8 (C-7), 31.0 (C-21), 28.2 (C-23), 28.0 (C-15), 25.0 (C-16), 23.7 (C-2), 23.6 (C-11), 23.4 (C-27), 21.4 (Ac), 21.4 (C-30), 18.3 (C-6), 17.4 (C-29), 17.1 (C-26), 16.9 (C-24), 15.7 (C-25) ppm; MS (ESI, MeOH): *m*/*z* = 661.4 (60%, [M + H]^+^), 685.5 (86%, [M + Na]^+^), 1343.3 (100%, [2M + Na]^+^); analysis calcd for C_38_H_61_ClN_2_O_5_ (661.37): C 69.01, H 9.30, N 4.24, Cl 5.36; found: C 68.80, H 9.61, N 4.01.

*Tri-tert-butyl 2,2’,2’’-[10-[2-[4-(3β-acetyloxy-urs-12-en-28-oyl)piperazin-1-yl]-2-oxoethyl]-1,4,7,10-tetraazacyclododecane-1,4,7-triyl]triacetate* (**22**). Compound **22** was synthesized from **6** and **19** according to general procedure D. Column chromatography (SiO_2_, CHCl_3_/MeOH 95:5) furnished compound **22** (88%); m.p. 237–240 °C (decomp.); [α]_D_ = +15.0° (c 0.3, CHCl_3_); R_f_ = 0.42 (CHCl_3_/MeOH 9:1); IR (ATR): ν = 2927*w*, 2928*w*, 2871*w*, 2829*w*, 1726*s*, 1646*m*, 1453*m*, 1424*w*, 1368*s*, 1305*m*, 1227*s*, 1160*s*, 1105*s*, 1004*m*, 975*m*, 754*m* cm^−1^; ^1^H NMR (400 MHz, CDCl_3_): δ = 5.19 (*t*, *J* = 3.5 Hz, 1H, 12-H), 4.47 (*dd*, *J* = 9.8, 6.0 Hz, 1H, 3-H), 3.93–2.05 (*m*, 32H, 31-H, 31′-H, 32-H, 32′-H, 8 × CH_2_ (cyclen), 3 × CH_2_ (acetate), 34-H), 2.40 (*d*, *J* = 11.0 Hz, 1H, 18-H), 2.19–2.09 (*m*, 1H, 16-H_a_), 2.02 (*s*, 3H, Ac), 1.93–1.85 (*m*, 2H, 11-H_a_, 11-H_b_), 1.82–1.67 (*m*, 3H, 15-H_a_, 16-H_b_, 22-H_a_), 1.65–1.21 (*m*, 12H, 2-H_a_, 2-H_b_, 22-H_b_, 1-H_a_, 9-H, 21-H_a_, 6-H_a_, 7-H_a_, 19-H, 21-H_b_, 6-H_b_, 7-H_b_), 1.43 (*s*, 9H, CH_3_ (*t*Butyl)), 1.42 (*s*, 9H; CH_3_ (*t*Butyl)), 1.42 (*s*, 9H, CH_3_ (*t*Butyl)), 1.10–0.96 (*m*, 3H, 1-H_b_, 15-H_b_, 20-H), 1.05 (*s*, 3H, 27-H), 0.92 (*d*, *J* = 6.3 Hz, 3H, 30-H), 0.91 (*s*, 3H, 25-H), 0.86 (*d*, *J* = 6.3 Hz, 3H, 29-H), 0.84 (*s*, 3H, 23-H), 0.82 (*s*, 3H, 24-H), 0.82–0.76 (*m*, 1H, 5-H), 0.71 (*s*, 3H, 26-H) ppm; ^13^C NMR (101 MHz, CDCl_3_): δ = 175.9 (C-28), 172.8 (CO, acetate), 172.7 (CO, acetate), 171.0 (Ac), 170.7 (C-33), 138.7 (C-13), 125.1 (C-12), 81.9 (C_q_, *t*Butyl), 81.7 (C_q_, *t*Butyl), 81.7 (C_q_, *t*Butyl), 81.0 (C-3), 55.8 (CH_2_, acetate), 55.8 (CH_2_, acetate), 55.7 (CH_2_, cyclen), 55.4 (C-5), 55.1 (C-18), 48.8 (C-17), 47.6 (C-9), 44.6 (C-31, C-31′, C-32, C-32′), 42.3 (C-14), 41.7 (C-34), 39.5 (C-8), 39.4 (C-19), 38.8 (C-20), 38.3 (C-1), 37.8 (C-4), 37.0 (C-10), 34.4 (C-22), 33.1 (C-7), 30.5 (C-21), 28.3 (C-15), 28.2 (C-23), 28.1 (CH_3_, *t*Butyl), 28.0 (CH_3_, *t*Butyl), 23.8 (C-27), 23.6 (C-2, C-16), 23.4 (C-11), 21.4 (Ac), 21.3 (C-30), 18.2 (C-6), 17.5 (C-29), 17.0 (C-26), 16.8 (C-24), 15.6 (C-25) ppm; MS (ESI, MeOH): *m*/*z* = 1143.7 (100%, [M + Na]^+^); analysis calcd for C_64_H_108_N_6_O_10_ (1121.60): C 68.54, H 9.71, N 7.49; found: C 68.31, H 10.03, N 7.27.

*Tribenzyl 2,2’,2’’-[10-[2-[4-(3β-acetyloxy-urs-12-en-28-oyl)piperazin-1-yl]-2-oxoethyl]-1,4,7,10-tetraazacyclododecane-1,4,7-triyl]triacetate* (**23**). Compound **23** was synthesized from **19** and **7** according to general procedure D. Column chromatography (SiO_2_, CHCl_3_/MeOH 95:5) furnished compound **23** (72%); m.p. 157–161 °C; [α]_D_ = +4.2° (c 0.300, CHCl_3_); R_f_ = 0.37 (CHCl_3_/MeOH 9:1); IR (ATR): ν = 2945*w*, 2836*w*, 1730*s*, 1638*m*, 1454*m*, 1424*w*, 1392*m*, 1370*m*, 1302*m*, 1243*s*, 1194*s*, 1105*s*, 1005*m*, 966*m*, 743*m*, 697*s* cm^−1^; UV-Vis (CHCl_3_): λ_max_ (logε) = 246 nm (3.96), 294 nm (3.47), 364 nm (3.23); ^1^H NMR (400 MHz, CDCl_3_): δ = 7.39–7.26 (*m*, 15H, 15 × CH (Bn)), 5.33–4.97 (*m*, 7H, 12-H, 3 × CH_2_ (Bn)), 4.45 (*dd*, *J* = 10.1, 5.9 Hz, 1H, 3-H), 4.02–2.05 (*m*, 34H, 31-H, 31‘-H, 32-H, 32‘-H, 8 × CH_2_ (cyclen), 3 × CH_2_ (acetate), 34-H, 16-H_a_, 18-H), 2.02 (*s*, 3H, Ac), 1.95–1.65 (*m*, 5H, 11-H_a_, 11-H_b_, 15-H_a_, 16-H_b_, 22-H_a_), 1.65–1.09 (*m*, 12H, 2-H_a_, 2-H_b_, 22-H_b_, 1-H_a_, 9-H, 21-H_a_, 6-H_a_, 7-H_a_, 19-H, 21-H_b_, 6-H_b_, 7-H_b_), 1.03 (*s*, 3H, 27-H), 1.08–0.95 (*m*, 3H, 1-H_b_, 15-H_b_, 20-H), 0.93 (*d*, *J* = 6.0 Hz, 3H, 30-H), 0.85 (*d*, *J* = 6.3 Hz, 3H, 29-H), 0.85 (*s*, 3H, 25-H), 0.82 (*s*, 3H, 23-H), 0.79 (*s*, 3H, 24-H), 0.79–0.71 (*m*, 1H, 5-H), 0.66 (*s*, 3H, 26-H) ppm; ^13^C NMR (101 MHz, CDCl_3_): δ = 175.9 (C-28), 173.6 (CO, acetate), 171.0 (Ac), 170.8 (C-33), 138.6 (C-13), 135.5 (C_i_, Bn), 135.3 (C_i_, Bn), 128.7 (CH, Bn), 128.7 (CH, Bn), 128.6 (CH, Bn), 128.6 (CH, Bn), 128.4 (CH, Bn), 128.4 (CH, Bn), 125.1 (C-12), 81.0 (C-3), 67.1 (CH_2_, Bn), 66.9 (CH_2_, Bn), 55.7 (CH_2_, acetate), 55.4 (CH_2_, cyclen), 55.2 (C-5), 55.1 (C-18), 48.7 (C-17), 47.6 (C-9), 44.8 (C-31, C-31‘, C-32, C-32‘), 42.2 (C-14), 42.0 (C-34), 39.5 (C-19), 39.5 (C-8), 38.8 (C-20), 38.3 (C-1), 37.7 (C-4), 36.9 (C-10), 34.5 (C-22), 33.1 (C-7), 30.6 (C-21), 28.2 (C-15), 28.1 (C-23), 23.6 (C-2, C-16), 23.5 (C-27), 23.4 (C-11), 21.4 (Ac), 21.3 (C-30), 18.2 (C-6), 17.5 (C-29), 17.0 (C-26), 16.8 (C-24), 15.5 (C-25) ppm; MS (ESI, MeOH): *m*/*z* = 1245.8 (100%, [M + Na]^+^); analysis calcd for C_73_H_102_N_6_O_10_ (1223.65): C 71.65, H 8.40, N 6.87; found: C 71.42, H 8.69, N 6.56.

*Tri-tert-butyl 2,2’,2’’-[10-[2-[2-(3β-acetyloxy-urs-12-en-28-oylamino)ethyl]amino-2-oxoethyl]-1,4,7,10-tetraazacyclododecane-1,4,7-triyl]triacetate* (**24**). Compound **24** was synthesized from **20** and **6** according to general procedure D. Column chromatography (SiO_2_, CHCl_3_/MeOH 95:5) furnished compound **24** (73%); m.p. 254–257 °C (decomp.); [α]_D_ = +33.8° (c 0.300, CHCl_3_); R_f_ = 0.40 (CHCl_3_/MeOH 9:1); IR (KBr): ν = 2971*m*, 2929*m*, 2829*w*, 1727*s*, 1668*m*, 1520*m*, 1454*m*, 1426*w*, 1368*s*, 1307*m*, 1228*s*, 1159*s*, 1106*s*, 1027*m*, 1006*m*, 975*m*, 755*m* cm^−1^; ^1^H NMR (400 MHz, CDCl_3_): δ = 8.03 (*s*, 1H, NH), 7.08 (*s*, 1H, NH), 5.46 (*t*, *J* = 3.4 Hz, 1H, 12-H), 4.47 (*dd*, *J* = 9.8, 6.1 Hz, 1H, 3-H), 3.80–2.05 (*m*, 28H, 31-H, 32-H, 34-H, 3 × CH_2_ (acetate), 8 × CH_2_ (cyclen)), 2.44–2.38 (*m*, 1H, 18-H), 2.02 (*s*, 3H, Ac), 2.00–1.67 (*m*, 6H, 15-H_a_, 11-H_a_, 11-H_b_, 16-H_a_, 16-H_b_, 22-H_a_), 1.66–1.19 (*m*, 12H, 1-H_a_, 2-H_a_, 2-H_b_, 22-H_b_, 9-H, 6-H_a_, 7-H_a_, 21-H_a_, 19-H, 6-H_b_, 21-H_b_, 7-H_b_), 1.44 (*s*, 9H, CH_3_ (*t*Butyl)), 1.43 (*s*, 9H, CH_3_ (*t*Butyl)), 1.43 (*s*, 9H, CH_3_ (*t*Butyl)), 1.15–0.98 (*m*, 3H, 20-H, 1-H_b_, 15-H_b_), 1.04 (*s*, 3H, 27-H), 0.91 (*s*, 3H, 25-H), 0.90 (*d*, *J* = 6.1 Hz, 3H, 30-H), 0.88 (*d*, *J* = 6.1 Hz, 3H, 29-H), 0.84 (*s*, 3H, 23-H), 0.83 (*s*, 3H, 24-H), 0.82–0.77 (*m*, 1H, 5-H), 0.74 (*s*, 3H, 26-H) ppm; ^13^C NMR (101 MHz, CDCl_3_): δ = 178.7 (C-28), 172.4 (CO, acetate), 171.7 (C-33), 171.1 (Ac), 139.0 (C-13), 125.5 (C-12), 82.1 (C_q_, *t*Butyl), 82.1 (C_q_, *t*Butyl), 82.1 (C_q_, *t*Butyl), 81.1 (C-3), 56.4 (3 × CH_2_, acetate), 55.8 (C-34), 55.8 (CH_2_, cyclen), 55.4 (C-5), 52.3 (C-18), 47.7 (C-9), 47.6 (C-17), 42.2 (C-14), 40.0 (C-32), 39.7 (C-19), 39.7 (C-8), 38.5 (C-20), 38.4 (C-31, C-1), 37.8 (C-4), 37.4 (C-22), 37.0 (C-10), 32.9 (C-7), 31.2 (C-21), 28.2 (C-23), 28.2 (CH_3_, *t*Butyl), 28.1 (CH_3_, *t*Butyl), 28.1 (CH_3_, *t*Butyl), 27.9 (C-15), 24.4 (C-16), 23.7 (C-2), 23.5 (C-27), 23.4 (C-11), 21.5 (C-30), 21.4 (Ac), 18.4 (C-6), 17.2 (C-29), 17.0 (C-26), 16.9 (C-24), 15.7 (C-25) ppm; MS (ESI, MeOH): *m*/*z* = 1117.7 (100%, [M + Na]^+^); analysis calcd for C_62_H_106_N_6_O_10_ (1095.56): C 67.97, H 9.75, N 7.67; found: C 67.68, H 10.02, N 7.41.

*Tribenzyl 2,2’,2’’-[10-[2-[2-(3β-acetyloxy-urs-12-en-28-oylamino)ethyl]amino-2-oxoethyl]-1,4,7,10-tetraazacyclododecane-1,4,7-triyl]triacetate* (**25**). Compound **25** was synthesized from **7** and **20** according to general procedure D. Column chromatography (SiO_2_, CHCl_3_/MeOH 95:5) furnished compound **25** (75%); m.p. 142–146 °C; [α]_D_ = +20.5° (c 0.390, CHCl_3_); R_f_ = 0.36 (CHCl_3_/MeOH 9:1); IR (ATR): ν = 2945*w*, 2832*w*, 1732*s*, 1663*m*, 1519*w*, 1454*m*, 1370*m*, 1305*m*, 1245*s*, 1194*s*, 1176*s*, 1105*s*, 1007*m*, 965*m*, 747*m*, 697*s* cm^−1^; UV-Vis (CHCl_3_): λ_max_ (logε) = 241 nm (3.89), 295 nm (3.29), 364 nm (3.08); ^1^H NMR (400 MHz, CDCl_3_): δ = 8.20 (*t*, *J* = 4.5 Hz, 1H, NH), 7.39–7.27 (*m*, 15H, CH (Bn)), 7.01 (*t*, *J* = 4.9 Hz, 1H, NH), 5.48 (*s*, 1H, 12-H), 5.26–5.05 (*m*, 6H, CH_2_ (Bn)), 4.47 (*dd*, *J* = 10.0, 5.7 Hz, 1H, 3-H), 3.77–2.07 (*m*, 28H, 31-H_a_, 31-H_b_, 32-H, 34-H, 3 × CH_2_ (acetate), 8 × CH_2_ (cyclen)), 2.41 (*d*, *J* = 10.7 Hz, 1H, 18-H), 2.02 (*s*, 3H, Ac), 2.00–1.64 (*m*, 6H, 16-H_a_, 16-H_b_, 11-H_a_, 11-H_b_, 22-H_a_, 15-H_a_), 1.64–1.19 (*m*, 12H, 1-H_a_, 2-H_a_, 2-H_b_, 22-H_b_, 9-H, 6-H_a_, 7-H_a_, 21-H_a_, 6-H_b_, 19-H, 21-H_b_, 7-H_b_), 1.17–0.97 (*m*, 3H, 20-H, 1-H_b_, 15-H_b_), 1.04 (*s*, 3H, 27-H), 0.89 (*d*, *J* = 6.3 Hz, 6H, 29-H, 30-H), 0.87 (*s*, 3H, 25-H), 0.83 (*s*, 3H, 23-H), 0.81 (*s*, 3H, 24-H), 0.80–0.76 (*m*, 1H, 5-H), 0.72 (*s*, 3H, 26-H) ppm; ^13^C NMR (101 MHz, CDCl_3_): δ = 178.8 (C-28), 173.2 (CO, acetate), 172.0 (C-33), 171.0 (Ac), 139.0 (C-13), 135.4 (C_i_, Bn), 135.4 (C_i_, Bn), 135.3 (C_i_, Bn), 128.8 (CH, Bn), 128.8 (CH, Bn), 128.7 (CH, Bn), 128.6 (CH, Bn), 125.6 (12-H), 81.0 (3-H), 67.3 (CH_2_, Bn), 67.3 (CH_2_, Bn), 67.2 (CH_2_, Bn), 56.8 (CH_2_, acetate), 55.4 (C-34), 55.3 (C-5), 55.3 (CH_2_, cyclen), 52.3 (C-18), 47.7 (C-17), 47.6 (C-9), 42.2 (C-14), 40.1 (C-32), 39.8 (C-19), 39.7 (C-8), 38.5 (C-20), 38.5 (C-31, C-1), 37.8 (C-4), 37.4 (C-22), 37.0 (C-10), 32.9 (C-7), 31.2 (C-21), 28.2 (C-23), 28.0 (C-15), 24.5 (C-16), 23.7 (C-2), 23.5 (C-27), 23.4 (C-11), 21.4 (Ac), 21.4 (C-30), 18.4 (C-6), 17.2 (C-29), 17.1 (C-26), 16.8 (C-24), 15.6 (C-25) ppm; MS (ESI, MeOH): *m*/*z* = 1219.8 (100%, [M + Na]^+^); analysis calcd for C_71_H_100_N_6_O_10_ (1197.61): C 71.21, H 8.42, N 7.02; found: 70.93, H 8.56, N 6.82.

*Tri-tert-butyl 2,2’,2’’-[10-[2-[2-[2-(3β-acetyloxy-urs-12-en-28-oylamino)ethoxy]ethyl]amino-2-oxoethyl]-1,4,7,10-tetraazacyclododecane-1,4,7-triyl]triacetate* (**26**). Compound **26** was synthesized from **6** and **21** according to general procedure D. Column chromatography (SiO_2_, CHCl_3_/MeOH 95:5) furnished compound **26** (74%); m.p. 263–266 °C (decomp.); [α]_D_ = +21.9° (c 0.315, CHCl_3_); R_f_ = 0.38 (CHCl_3_/MeOH 9:1); IR (ATR): ν = 2972*m*, 2930*m*, 1726*s*, 1166*m*, 1523*w*, 1453*m*, 1368*s*, 1307*m*, 1228*s*, 1160*s*, 1106*s*, 1026*m*, 1006*m*, 975*m*, 755*m* cm^−1^; ^1^H NMR (400 MHz, CDCl_3_): δ = 7.82 (*t*, *J* = 5.6 Hz, 1H, NH), 6.46 (*t*, *J* = 5.1 Hz, 1H, NH), 5.33 (*t*, *J* = 3.7 Hz, 1H, 12-H), 4.47 (*dd*, *J* = 10.2, 5.9 Hz, 1H, 3-H), 3.56–3.30 (*m*, 9H, 32-H, 31-H_a_, 33-H, 36-H, 34-H), 3.28–3.16 (*m*, 1H, 31-H_b_), 3.15–2.05 (*m*, 22H, 3 × CH_2_ (acetate), 8 × CH_2_ (cyclen)), 2.02 (*s*, 3H, Ac), 2.02–1.21 (*m*, 19H, 18-H, 16-H_a_, 11-H_a_, 11-H_b_, 22-H_b_, 16-H_b_, 15-H_a,_ 1-H_a_, 2-H_a_, 2-H_b_, 9-H, 6-H_a_, 21-H_a_, 7-H_a_, 22-H_b_, 19-H, 6-H_b_, 21-H_b_, 7-H_b_), 1.44 (*s*, 9H, CH_3_ (*t*Butyl)), 1.43 (*s*, 18H, CH_3_ (*t*Butyl)), 1.06 (*s*, 3H, 27-H), 1.05–0.94 (*m*, 3H, 1-H_b_, 15-H_b_, 20-H), 0.92 (*s*, 3H, 25-H), 0.92 (*d*, *J* = 6.2 Hz, 3H, 30-H), 0.86 (*d*, *J* = 6.5 Hz, 3H, 29-H), 0.84 (*s*, 3H, 23-H), 0.83 (*s*, 3H, 24-H), 0.82–0.77 (*m*, 1H, 5-H), 0.76 (*s*, 3H, 26-H) ppm; ^13^C NMR (101 MHz, CDCl_3_): δ = 178.3 (C-28), 173.0 (CO, acetate), 172.5 (CO, acetate), 172.1 (C-35), 171.1 (Ac), 139.3 (C-13), 125.7 (C-12), 82.1 (C_q_, *t*Butyl), 82.0 (C_q_, *t*Butyl), 81.9 (C_q_, *t*Butyl), 81.0 (C-3), 69.5 (C-33), 69.0 (C-32), 56.5 (C-36), 55.9 (CH_2_, acetate), 55.8 (CH_2_, acetate), 55.7 (CH_2_, cyclen), 55.4 (C-5), 53.4 (C-18), 47.8 (C-17), 47.6 (C-9), 42.4 (C-14), 39.8 (C-19), 39.7 (C-8), 39.2 (C-31), 39.0 (C-34), 39.0 (C-20), 38.4 (C-1), 37.8 (C-4), 37.3 (C-22), 37.0 (C-10), 32.9 (C-7), 31.1 (C-21), 28.2 (CH_3_, *t*Butyl), 28.0 (CH_3_, *t*Butyl), 28.0 (C-23), 28.0 (C-15), 24.9 (C-16), 23.7 (C-2), 23.5 (C-11), 23.4 (C-27), 21.4 (Ac), 21.4 (C-30), 18.3 (C-6), 17.3 (C-29), 17.0 (C-26), 16.8 (C-24), 15.7 (C-25) ppm; MS (ESI, MeOH): *m*/*z* = 1161.7 (100%, [M + Na]^+^); analysis calcd for C_64_H_110_N_6_O_11_ (1139.6): C 67.45, H 9.73, N 7.37; found: C 67.31, H 9.87, N 7.09.

*Tribenzyl 2,2’,2’’-[10-[2-[2-[2-(3β-acetyloxy-urs-12-en-28-oylamino)ethoxy]ethyl]amino-2-oxoethyl]-1,4,7,10-tetraazacyclododecane-1,4,7-triyl]triacetate* (**27**). Compound **27** has been synthesized from **7** and **21** according to general procedure D. Column chromatography (SiO_2_, CHCl_3_/MeOH 95:5) furnished compound **27** (62%); m.p. 137–141 °C; [α]_D_ = +15.4° (c 0.350, CHCl_3_); R_f_ = 0.35 (CHCl_3_/MeOH 9:1); IR (ATR): ν = 2945*w*, 2830*w*, 1731*s*, 1662*m*, 1523*w*, 1454*m*, 1390*m*, 1370*m*, 1305*m*, 1245*s*, 1193*s*, 1105*s*, 1026*m*, 1008*m*, 967*m*, 747*m*, 697*m* cm^−1^; UV-Vis (CHCl_3_): λ_max_ (logε) = 244 nm (3.73), 294 nm (3.36), 363 nm (3.12); ^1^H NMR (400 MHz, CDCl_3_): δ = 8.02 (*t*, *J* = 5.4 Hz, 1H, NH), 7.37–7.27 (*m*, 15H, CH (Bn)), 6.48 (*t*, *J* = 5.1 Hz, 1H, NH), 5.32 (*t*, *J* = 3.5 Hz, 1H, 12-H), 5.24–5.14 (*m*, 4H, CH_2_ (Bn)), 5.14–5.05 (*m*, 2H, CH_2_ (Bn)), 4.46 (*dd*, *J* = 10.2, 5.7 Hz, 1H, 3-H), 3.59–3.33 (*m*, 9H, 32-H, 33-H, 31-H_a_, 34-H, 36-H), 3.33–2.01 (*m*, 23H, 31-H_b_, 8 × CH_2_ (cyclen), 3 × CH_2_ (acetate)), 2.02 (*s*, 3H, Ac), 2.00–1.17 (*m*, 19H, 18-H, 16-H_a_, 11-H_a_, 11-H_b_, 22-H_a_, 16-H_b_, 15-H_a_, 1-H_a_, 2-H_a_, 2-H_b_, 9-H, 6-H_a_, 22-H_b_, 7-H_a_, 21-H_a_, 19-H, 6-H_b_, 7-H_b_, 21-H_b_), 1.05 (*s*, 3H, 27-H), 1.04 – 0.94 (*m*, 3H, 1-H_b_, 15-H_b_, 20-H), 0.90 (*s*, 3H, 25-H), 0.90 (*d*, *J* = 5.8 Hz, 3H, 30-H), 0.85 (*d*, *J* = 6.6 Hz, 3H, 29-H), 0.84 (*s*, 3H, 23-H), 0.82 (*s*, 3H, 24-H), 0.81–0.76 (*m*, 1H, 5-H), 0.75 (*s*, 3H, 26-H) ppm; ^13^C NMR (101 MHz, CDCl_3_): δ = 178.3 (C-28), 173.3 (CO, acetate), 173.1 (CO, acetate), 172.3 (C-35), 171.1 (Ac), 139.3 (C-13), 135.4 (C_i_, Bn), 135.3 (C_i_, Bn), 128.7 (CH, Bn), 128.7 (CH, Bn), 128.7 (CH, Bn), 128.6 (CH, Bn), 128.5 (CH, Bn), 125.7 (C-12), 81.0 (C-3), 69.6 (C-33), 68.9 (C-32), 67.2 (CH_2_, Bn), 67.2 (CH_2_, Bn), 56.9 (C-36), 55.4 (3 × CH_2_, acetate), 55.4 (C-5), 55.3 (8 × CH_2_, cyclen), 53.3 (C-18), 47.8 (C-17), 47.6 (C-9), 42.4 (C-14), 39.8 (C-19), 39.7 (C-8), 39.2 (C-31, C-34), 38.9 (C-20), 38.4 (C-1), 37.8 (C-4), 37.3 (C-22), 36.9 (C-10), 32.9 (C-7), 31.1 (C-21), 28.2 (C-23), 28.0 (C-15), 24.8 (C-16), 23.6 (C-2), 23.5 (C-11), 23.4 (C-27), 21.4 (Ac), 21.4 (C-30), 18.3 (C-6), 17.3 (C-29), 17.1 (C-26), 16.8 (C-24), 15.7 (C-25) ppm; MS (ESI, MeOH): *m*/*z* = 1263.9 (100%, [M + Na]^+^); analysis calcd for C_73_H_104_N_6_O_11_ (1241.67): C 70.62, H 8.44, N 6.77; found: C 70.41, H 8.69, N 6.41.

*(3β) N-(2-Aminoethyl)-3-hydroxy-urs-12-en-28-amide* (**28**). Method A: The synthesis was performed according to the procedure given in the Supplementary material in 82%. *Method B*: Ursolic acid (100 mg, 0.20 mmol), HOBt·H_2_O (37 mg, 0.24 mmol) and EDC·HCl (46 mg, 0.24 mmol) were dissolved in dry DMF (5 mL), and the mixture was stirred for 30 min at 25 °C. Ethylene diamine (55 µL, 0.82 mmol) was added to the mixture, and stirring was continued for 24 h at 25 °C. Usual aqueous work-up followed by column chromatography (silica gel, CHCl_3_/MeOH/NH_4_OH 90:10:0.1) gave **28** (46%). Analytical data of this compound can be found in the supplementary material.

*Tri-tert-butyl 2,2’,2’’-[10-[2-[2-(3β-hydroxy-urs-12-en-28-oylamino)ethyl]amino-2-oxoethyl]-1,4,7,10-tetraazacyclododecane-1,4,7-triyl]triacetate* (**29**). Method A: To a solution of DOTA-tris(tert-butyl ester) (58 mg, 0.10 mmol) in dry DMF (8 mL) were added HOBt·H_2_O (29 mg, 0.19 mmol) and EDC·HCl (29 mg, 0.15 mmol). After stirring for 30 min at 25 °C, a solution of **28** (71 mg, 0.14 mmol) in dry DMF (2 mL) was added and stirring was continued for 5 days. After usual aqueous work-up, the solvent was removed under reduced pressure and the crude product was subjected to column chromatography (silica gel, CHCl_3_/MeOH 9:1) yielding compound **29** as colorless solid. Yield: 49%. Method B: Compound **24** (50 mg, 0.10 mmol) was dissolved in methanol (7 mL) and a solution of potassium hydroxide (12 mg, 0.21 mmol) in methanol (1 mL) was added. The mixture was stirred at 25 °C for 24 h. After completion of the reaction (as indicated by TLC) and usual work-up, the solvent was removed under reduced pressure, and the residue was subjected to column chromatography (silica gel, CHCl_3_/MeOH 9:1) affording **29** (yield: 86%); m.p. 136–139 °C; [α]_D_ = −44.4° (c 0.330, MeOH); R_f_ = 0.29 (CHCl_3_/MeOH 9:1); IR (KBr): ν = 3300*br* *w*, 2973*w*, 2928*m*, 2869*w*, 1728*s*, 1668*m*, 1525*m*, 1455*m*, 1425*w*, 1368*s*, 1307*m*, 1227*s*, 1159*s*, 1121*m*, 1106*s*, 1047*w*, 1006*w* cm^−1^; ^1^H NMR (400 MHz, CDCl_3_): δ = 8.84 (*s*, 1H, NH), 7.55 (*s*, 1H, NH), 5.36 (*t*, *J* = 3.5 Hz, 1H, 12-H), 3.59–2.05 (*m*, 28H, 31-H, 32-H, 33-H, 3 × CH_2_ (acetate), 8 × CH_2_ (cyclen)), 3.20 (*dd*, *J* = 11.1, 4.8 Hz, 1H, 3-H), 2.34 (*d*, *J* = 11.0 Hz, 2H, 18-H), 2.03–1.96 (*m*, 1H, 16-H_a_), 1.95–1.83 (*m*, 3H, 11-H_a_, 11-H_b_, 16-H_b_), 1.83–1.72 (*m*, 1H, 22-H_a_), 1.64–1.21 (*m*, 13H, 1-H_a_, 15-H_a_, 2-H_a_, 2-H_b_, 22-H_b_, 6-H_a_, 9-H, 7-H_a_, 21-H_a_, 19-H, 6-H_b_, 7-H_b_, 21-H_b_), 1.45 (*s*, 9H, CH_3_ (*t*Butyl)), 1.43 (*s*, 18H, CH_3_ (*t*Butyl)), 1.05 (*s*, 3H, 27-H), 1.04–0.97 (*m*, 3H, 1-H_b_, 15-H_b_, 20-H), 0.97 (*s*, 3H, 23-H), 0.89 (*d*, *J* = 6.3 Hz, 3H, 30-H), 0.89 (*s*, 3H, 25-H), 0.85 (*d*, *J* = 6.4 Hz, 3H, 29-H), 0.76 (*s*, 3H, 24-H), 0.75 (*s*, 3H, 26-H), 0.72–0.68 (*m*, 1H, 5-H) ppm; ^13^C NMR (101 MHz, CDCl_3_): δ = 178.4 (C-28), 172.4 (CO, acetate), 171.8 (C-33), 138.9 (C-13), 125.3 (C-12), 82.2 (C_q_, *t*Butyl), 82.1 (C_q_, *t*Butyl), 82.1 (C_q_, *t*Butyl), 79.2 (C-3), 56.0 (CH_2_, acetate), 55.9 (C-34), 55.8 (CH_2_, cyclen), 55.3 (C-5), 52.5 (C-18), 47.8 (C-9), 47.5 (C-17), 42.2 (C-14), 40.0 (C-32), 39.7 (C-8), 39.6 (C-19), 38.9 (C-4), 38.7 (C-20), 38.7 (C-31, C-1), 37.4 (C-22), 37.1 (C-10), 33.2 (C-7), 31.3 (C-21), 28.3 (C-23), 28.2 (CH_3_, *t*Butyl), 28.2 (CH_3_, *t*Butyl), 28.1 (CH_3_, *t*Butyl), 28.0 (C-15), 27.4 (C-2), 24.2 (C-16), 23.6 (C-27), 23.5 (C-11), 21.5 (C-30), 18.6 (C-6), 17.2 (C-29), 17.1 (C-26), 15.8 (C-24), 15.7 (C-25) ppm; MS (ESI, MeOH): *m*/*z* = 1075.7 (100%, [M + Na]^+^); analysis calcd for C_60_H_104_N_6_O_9_ (1053.53): C 68.40, H 9.95, N 7.98; found: C 60.09, H 10.11, N 7.83.

## Figures and Tables

**Table 1:** Cytotoxicity of UA-DOTA conjugates (**12**–**15**, **22**–**27**, **29**), DOTA precursors (**6**,**7**), ursolic acid (UA), and doxorubicin hydrochloride (DRC): EC_50_ values from SRB assays after 72 h of treatment are given in µM (n.d. not detected; n.s. not soluble); the values are averaged from three independent experiments each performed in triplicate; confidence interval CI = 95%.

	A375	A2780	HT29	MCF-7	FaDu	NIH 3T3
UA	n.d.	11.7 ± 0.6	10.6 ± 0.7	12.7 ± 0.1	n.d.	13.1 ± 1.1
**6**	12.4 ± 2.7	10.5 ± 3.0	8.7 ± 2.7	11.3 ± 2.3	9.5 ± 0.9	17.3 ± 0.1
**7**	4.7 ± 0.3	3.8 ± 0.3	2.0 ± 0.2	4.1 ± 0.5	3.9 ± 0.7	4.5 ± 0.3
**13**	n.d.	2.1 ± 0.4	4.0 ± 0.4	3.3 ± 0.2	6.0 ± 0.1	3.6 ± 0.5
**15**	>60	>60	>60	>60	>60	>60
**22**	1.5 ± 0.4	1.9 ± 0.3	5.7 ± 0.5	4.4 ± 0.7	3.7 ± 0.6	4.6 ± 1.0
**24**	2.0 ± 0.1	1.7 ± 0.1	2.3 ± 0.3	1.8 ± 0.1	2.0 ± 0.2	1.4 ± 0.1
**29**	2.0 ± 0.3	1.6 ± 0.5	1.7 ± 0.4	n.d.	2.9 ± 0.4	2.3 ± 0.7
**12, 14, 23, 25–27**	n.s.	n.s	n.s	n.s.	n.s.	n.s.
DRC	n.d.	0.01 ± 0.01	0.9 ± 0.2	1.1 ± 0.3	n.d.	0.06 ± 0.03

**Table 2:** Selectivity of selected UA-DOTA conjugates (**13**, **22**, **24,** and **29**), DOTA precursors (**6**, **7**), ursolic acid (UA) and doxorubicin hydrochloride (DRC): Selectivity index (SI) is defined as: SI = EC_50_ (NIH 3T3)/EC_50_ (tumor cell line).

	A375	A2780	HT29	MCF-7	FaDu
UA	-	1.12	1.24	1.03	-
**6**	1.40	1.65	1.99	1.53	1.82
**7**	0.96	1.18	2.25	1.10	1.15
**13**	-	1.71	0.90	1.09	0.60
**22**	3.07	2.42	0.81	1.05	1.24
**24**	0.70	0.82	0.61	0.78	0.70
**29**	1.15	1.44	1.35	-	0.79
DRC	-	6.00	0.07	0.05	-

## Supplementary Materials

Supplementary data related to this article including experimental procedures for compounds **10**, **17**, **18**, and **28**, Figure S1: Extended cytotoxicity investigation after treatment of A375 cells with **22** (3.0 µM) for 24 h, representative NMR spectra and calculation of ADMET parameters for compounds **22** and **24** can be found online.
