# Supplementary material for: Synthesis and Cytotoxicity Evaluation of DOTA-Conjugates of Ursolic Acid
Source: Molecules. 2019 Jun 17;24(12):2254. doi: 10.3390/molecules24122254 (PMC6630699; doi:10.3390/molecules24122254)
Supplement: Supplementary file 1 [file molecules-24-02254-s001.pdf]

Supplementary Material

Synthesis and Cytotoxicity Evaluation of DOTA-Conjugates of Ursolic Acid

Michael Kahnt [1], Sophie Hoenke [1], Lucie Fischer [1], Ahmed Al-Harrasi [2] and René Csuk [1,*]

[1] Organic Chemistry, Martin-Luther-University Halle-Wittenberg, Kurt-Mothes-Str. 2, D-06120 Halle (Saale), Germany; michael.kahnt@chemie.uni-halle.de (M.K.); sophie.hoenke@chemie.uni-halle.de (S.H.); lucie.fischer2018@gmx.de (L.F.)

[2] Natural and Medical Sciences Research Center, University of Nizwa, PO Box 33, Birkat Al-Mauz, Nizwa 616, Oman; aharrasi@unizwa.edu.om

***** Correspondence: rene.csuk@chemie.uni-halle.de; Tel.: +49-345-55-25660

1 Experimental Procedures and Analytical Data

*(3β)-N-(2-Piperazin-1-ylethyl)-3-acetyloxy-urs-12-en-28-amide (**10**)*

Compound **10** was prepared from **9** according to general procedure B using 1-(2-aminoethyl)-piperazine. Column chromatography (SiO_2, $CHCl_3$/MeOH 9:1) gave **10** (yield: 82%); m.p. 145–147 °C (lit.: 147–150 °C [26]); $[\alpha]_D$ = +35.9° (*c* 0.365, $CHCl_3$); R_f = 0.29 ($CHCl_3$/MeOH 9:1); IR (KBr): ν = 3441*s*, 2947*m*, 1734*m*, 1636*m*, 1458*w*, 1370*w*, 1247*m*, 1027*w* cm^{-1}; ^{1}H NMR (400 MHz, $CDCl_3$): δ = 6.42 (*t*, *J* = 4.6 Hz, 1H, N*H*), 5.30 (*t*, *J* = 3.6 Hz, 1H, 12-H), 4.49 (*dd*, *J* = 10.4, 5.3 Hz, 1H, 3-H), 3.42 – 3.32 (*m*, 1H, 31-H_a), 3.23 – 3.13 (*m*, 1H, 31-H_b), 2.93 (*t*, *J* = 4.9 Hz, 4H, 34-H, 34'-H), 2.49 – 2.37 (*m*, 6H, 32-H, 33-H, 33'-H), 2.04 (*s*, 3H, Ac), 2.02 – 1.80 (*m*, 5H, 16-H_a, 11-H_a, 11-H_b, 22-H_a, 18-H), 1.80 – 1.22 (*m*, 14H, 16-H_b, 15-H_a, 1-H_a, 2-H_a, 2-H_b, 9-H, 6-H_a, 21-H_a, 7-H_a, 22-H_b, 19-H, 6-H_b, 21-H_b, 7-H_b), 1.08 (*s*, 3H, 27-H), 1.07 – 0.94 (*m*, 3H, 1-H_b, 15-H_b, 20-H), 0.96 – 0.94 (*m*, 3H, 30-H), 0.93 (*s*, 3H, 25-H), 0.88 (*d*, *J* = 6.5 Hz, 3H, 29-H), 0.86 (*s*, 3H, 23-H), 0.85 (*s*, 3H, 24-H), 0.84 – 0.78 (*m*, 1H, 5-H), 0.77 (*s*, 3H, 26-H) ppm; ^{13}C NMR (101 MHz, $CDCl_3$): δ = 178.0 (C-28), 171.1 (Ac), 139.7 (C-13), 125.5 (C-12), 81.0 (C-3), 57.1 (C-32), 55.4 (C-5), 54.1 (C-18), 53.9 (C-33), 47.9 (C-17), 47.6 (C-9), 46.1 (C-34), 42.6 (C-14), 39.9 (C-19), 39.7 (C-8), 39.3 (C-20), 38.4 (C-1), 37.8 (C-4), 37.5 (C-22), 37.0 (C-10), 35.8 (C-31), 32.8 (C-7), 31.1 (C-21), 28.2 (C-23), 28.0 (C-15), 25.0 (C-16), 23.7 (C-2), 23.6 (C-11), 23.4 (C-27), 21.4 (Ac), 21.3 (C-30), 18.3 (C-6), 17.5 (C-29), 17.1 (C-26), 16.9 (C-24), 15.7 (C-25) ppm; MS (ESI, MeOH): *m/z* = 610 (100 %, $[M+H]^+$); analysis calcd for $C_{38}H_{63}N_3O_3$ (609.94): C 74.83, H 10.41, N 6.89; found: C 74.57, H 10.69, N 6.64.

*(3β)-N-(2-Aminoethyl)-3-acetyloxy-urs-12-en-28-amide (**17**)*

Compound **17** was prepared from **9** according to general procedure B using ethylenediamine. Column chromatography (SiO_2, $CHCl_3$/MeOH 9:1) gave **17** (yield: 80%); m.p. 202–205 °C

(lit.: 140–142 °C[26]); $[\alpha]_D$ = +39.4 ° (*c* 0.355, $CHCl_3$); R_f = 0.48 ($CHCl_3$/MeOH 9:1); IR (KBr): ν = 3413*br s*, 2948*s*, 1735*s*, 1633*s*, 1526*s*, 1456*s*, 1370*s*, 1247*s*, 1174*w*, 1147*w*, 1092*w*, 1028*s*, 1006*m*, 986*m*, 755*m* cm^{-1}; ^1H NMR (500 MHz, $CDCl_3$): δ = 6.88 (*t*, *J* = 5.3 Hz, 1H, NH), 5.34 (*t*, *J* = 3.3 Hz, 1H, 12-H), 4.49 (*dd*, *J* = 10.0, 5.9 Hz, 1H, 3-H), 3.62 – 3.54 (*m*, 1H, 31-H_a), 3.38 – 3.30 (*m*, 1H, 31-H_b), 3.13 – 3.01 (*m*, 2H, 32-H_a, 32-H_b), 2.09 – 2.04 (*m*, 1H, 18-H), 2.04 (*s*, 3H, Ac), 2.03 – 1.87 (*m*, 3H, 16-H_a, 11-H_a, 11-H_b), 1.82 – 1.22 (*m*, 15H, 22-H_a, 16-H_b, 1-H_a, 15-H_a, 2-H_a, 2-H_b, 9-H, 22-H_b, 6-H_a, 21-H_a, 7-H_a, 19-H, 6-H_b, 7-H_b, 21-H_b), 1.08 (*s*, 3H, 27-H), 1.07 – 0.95 (*m*, 3H, 1-H_b, 15-H_b, 20-H), 0.96 – 0.92 (*m*, 4H, 25-H, 20-H), 0.89 – 0.85 (*m*, 6H, 23-H, 29-H), 0.85 (*s*, 3H, 24-H), 0.84 – 0.80 (*m*, 1H, 5-H), 0.74 (*s*, 3H, 26-H) ppm; ^{13}C NMR (126 MHz, $CDCl_3$): δ = 180.2 (C-28), 171.1 (Ac), 139.3 (C-13), 126.0 (C-12), 81.0 (C-3), 55.4 (C-5), 53.1 (C-18), 47.9 (C-17), 47.6 (C-9), 42.4 (C-14), 40.6 (C-32), 39.8 (C-19), 39.7 (C-8), 39.0 (C-20), 38.7 (C-31), 38.5 (C-1), 37.8 (C-4), 37.4 (C-22), 37.0 (C-10), 32.8 (C-7), 31.0 (C-21), 28.2 (C-23), 28.0 (C-15), 24.8 (C-16), 23.7 (C-2), 23.5 (C-11), 23.5 (C-27), 21.4 (Ac), 21.3 (C-30), 18.3 (C-6), 17.4 (C-29), 17.2 (C-26), 16.9 (C-24), 15.7 (C-25) ppm; MS (ESI, MeOH): *m/z* = 541 (100 %, $[M+H]^+$); analysis calcd for $C_{34}H_{56}N_2O_3$ (540.83): C 75.51, H 10.44, N 5.18; found: C 75.32, H 10.61, N 5.01.

*(3β)-N-[2-(2-Aminoethoxy)ethyl]-3-acetyloxy-urs-12-en-28-amide (**18**)*

Compound **18** was prepared from **9** according to general procedure B using 2,2'-oxybis(ethylamine). Column chromatography (SiO_2, $CHCl_3$/MeOH/NH_4OH 90:10:0.1) gave **18** (yield: 78%); m.p. 91–94 °C; $[\alpha]_D$ = +18.3 ° (*c* 0.310, $CHCl_3$); R_f = 0.39 ($CHCl_3$/MeOH 9:1); IR (KBr): ν = 3424*br s*, 2927*s*, 2871*s*, 1735*s*, 1640*s*, 1529*m*, 1455*m*, 1370*m*, 1247*s*, 1120*m*, 1027*m* cm^{-1}; ^1H NMR (500 MHz, $CDCl_3$): δ = 6.29 (*t*, *J* = 5.0 Hz, 1H, NH), 5.28 (*t*, *J* = 3.6 Hz, 1H, 12-H), 4.48 (*dd*, *J* = 10.9, 5.3 Hz, 1H, 3-H), 3.57 – 3.44 (*m*, 5H, 31-H_a, 32-H, 33-H), 3.30 – 3.22 (*m*, 1H, 31-H_b), 2.87 (*t*, J = 5.3 Hz, 2H, 34-H), 2.03 (*s*, 3H, Ac), 2.01 – 1.81 (*m*, 5H, 16-H_a, 11-H_a, 11-H_b, 18-H, 22-H_a), 1.78 – 1.22 (*m*, 14H, 16-H_b, 15-H_a, 1-H_a, 2-H_a, 2-H_b, 9-H, 6-H_a, 21-H_a, 7-H_a, 22-H_b, 19-H, 6-H_b, 21-H_b, 7-H_b), 1.08 (*s*, 3H, 27-H), 1.12 – 1.00 (*m*, 2H, 1-H_b, 15-H_b), 0.99 – 0.90 (*m*, 4H, 30-H, 20-H), 0.93 (*s*, 3H, 25-H), 0.86 (*d*, *J* = 6.6 Hz, 3H, 29-H), 0.85 (*s*, 3H, 23-H), 0.84 (*s*, 3H, 24-H), 0.83 – 0.80 (*m*, 1H, 5-H), 0.78 (*s*, 3H, 26-H) ppm; ^{13}C NMR (126 MHz, $CDCl_3$): δ = 178.3 (C-28), 171.1 (Ac), 139.8 (C-13), 125.7 (C-12), 81.0 (C-3), 73.2 (C-33), 69.6 (C-32), 55.4 (C-5), 53.9 (C-18), 47.9 (C-17), 47.6 (C-9), 42.6 (C-14), 42.0 (C-34), 39.9 (C-19), 39.7 (C-8), 39.3 (C-31), 39.2 (C-20), 38.4 (C-1), 37.8 (C-4), 37.3 (C-22), 37.0 (C-10), 32.8 (C-7), 31.0 (C-21), 28.2 (C-23), 28.0 (C-15), 25.0

(C-16), 23.7 (C-2), 23.6 (C-11), 23.4 (C-27), 21.4 (Ac), 21.4 (C-30), 18.3 (C-6), 17.4 (C-29), 17.0 (C-26), 16.8 (C-24), 15.7 (C-25) ppm; MS (ESI, MeOH): *m/z* = 585 (100 %, $[M+H]^+$); analysis calcd for $C_{36}H_{60}N_2O_4$ (584.89): C 73.93, H 10.34, N 4.79; found: C 73.77, H 10.51, N 4.56.

*(3β)-N-(2-Aminoethyl)-3-hydroxy-urs-12-en-28-amide (**28**)*

To a solution of compound **17** (0.33 mmol) in methanol (10 mL) was added a solution of potassium hydroxide (1.65 mmol) in methanol (2 mL). The mixture was stirred at 25 ℃ for 2 days. After completion of the reaction (as indicated by TLC), aq. HCl was added until pH = 7. After usual work-up, the solvent was removed under reduced pressure, and the residue was subjected to column chromatography (SiO_2, $CHCl_3$/MeOH/NH_4OH 90:10:0.1) affording **28** (yield: 85%); m.p. 139–142 ℃ (lit.: 145–147 ℃[27]); $[\alpha]_D$ = +38.6 ° (*c* 0.300, $CHCl_3$); R_f = 0.34 ($CHCl_3$/MeOH 9:1); IR (KBr): ν = 3425*br s*, 2926*s*, 1638*m*, 1529*m*, 1454*m*, 1386*w*, 1092*w*, 1046*m*, 755*m* cm^{-1}; ^1H NMR (400 MHz, $CDCl_3$): δ = 6.36 (*t*, *J* = 5.4 Hz, 1H, N*H*), 5.33 (*t*, *J* = 3.4 Hz, 1H, 12-H), 3.46 – 3.36 (*m*, 1H, 31-H_a), 3.21 (*dd*, *J* = 11.1, 4.7 Hz, 1H, 3-H), 3.13 – 3.02 (*m*, 1H, 31-H_b), 2.82 (*t*, *J* = 5.9 Hz, 2H, 32-H_a, 32-H_b), 2.05 – 1.82 (*m*, 5H, 16-H_a, 11-H_a, 11-H_b, 18-H, 22-H_a), 1.77 – 1.23 (*m*, 14H, 16-H_b, 15-H_a, 1-H_a, 2-H_a, 2-H_b, 9-H, 6-H_a, 21-H_a, 7-H_a, 22-H_b, 19-H, 6-H_b, 21-H_b, 7-H_b), 1.09 (*s*, 3H, 27-H), 1.07 – 0.99 (*m*, 2H, 15-H_b, 1-H_b), 0.98 (*s*, 3H, 23-H), 0.96 – 0.93 (*m*, 4H, 20-H, 30-H), 0.91 (*s*, 3H, 25-H), 0.87 (*d*, *J* = 6.5 Hz, 3H, 29-H), 0.78 (*s*, 6H, 24-H, 26-H), 0.74 – 0.69 (*m*, 1H, 5-H) ppm; ^{13}C NMR (101 MHz, $CDCl_3$): δ = 178.8 (C-28), 139.7 (C-13), 125.9 (C-12), 79.1 (C-3), 55.3 (C-5), 53.9 (C-18), 48.0 (C-17), 47.7 (C-9), 42.6 (C-14), 41.8 (C-31), 41.3 (C-32), 39.9 (C-19), 39.7 (C-8), 39.2 (C-20), 38.9 (C-4), 38.8 (C-1), 37.5 (C-22), 37.1 (C-10), 32.9 (C-7), 31.1 (C-21), 28.3 (C-23), 28.0 (C-15), 27.4 (C-2), 25.0 (C-16), 23.6 (C-11), 23.4 (C-27), 21.4 (C-30), 18.4 (C-6), 17.4 (C-29), 17.1 (C-26), 15.8 (C-24), 15.7 (C-25) ppm; MS (ESI, MeOH): *m/z* = 499 (100 %, $[M+H]^+$); analysis calcd for $C_{32}H_{54}N_2O_2$ (498.80): C 77.06, H 10.91, N 5.62; found: C 76.92, H 11.08, N 5.40.

2 Cytotoxicity evaluation of compound 22 (24 h)

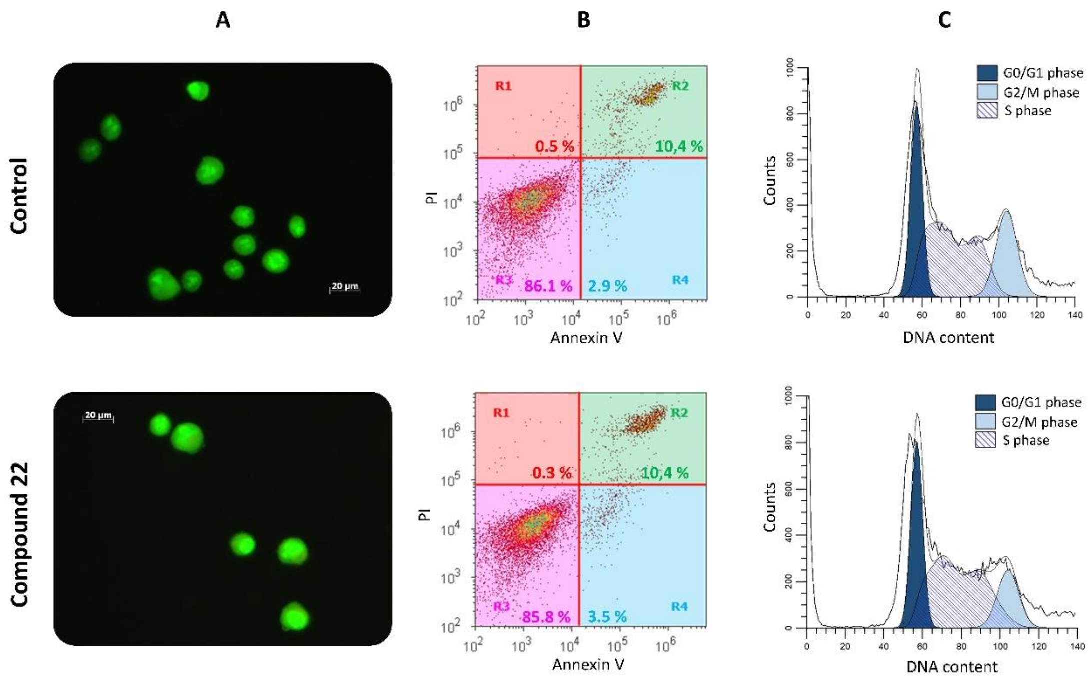

Fig. S1: Extended cytotoxicity investigation after treatment of A375 cells with **22** (3.0 µM) for 24 h: (**A**) Fluorescence microscopic images (scale bar 20 µm), AO and PI were used; (**B**) Annexin V-FITC/PI assay. Examples of density plots determined by flow cytometry (Attune® Cytometric Software vl 1.2.5), R1: necrotic, R2: secondary necrotic/late stage apoptotic, R3: vital, R4: apoptotic; (**C**) Representative examples for cell cycle evaluation via ModFit LT 5.0.

3 Representative NMR spectra

NMR spectra of 6

^{1}H NMR (400 MHz, $CDCl_3$)

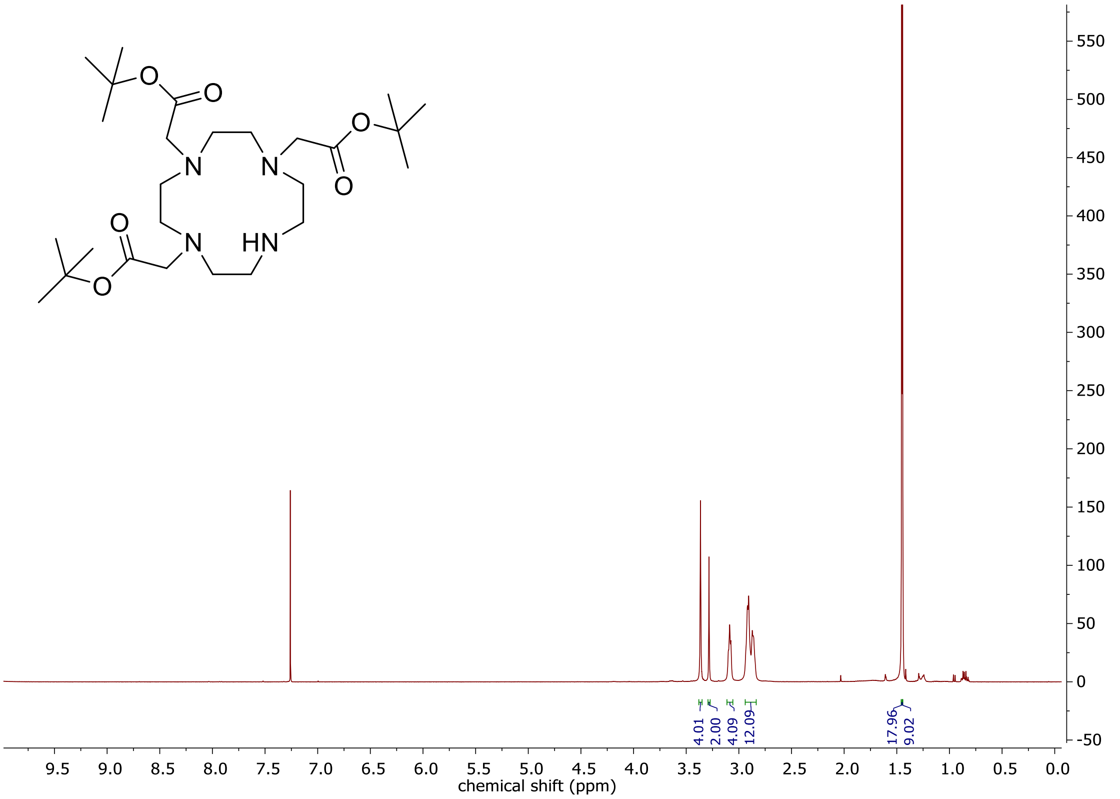

^{13}C NMR (101 MHz, $CDCl_3$)

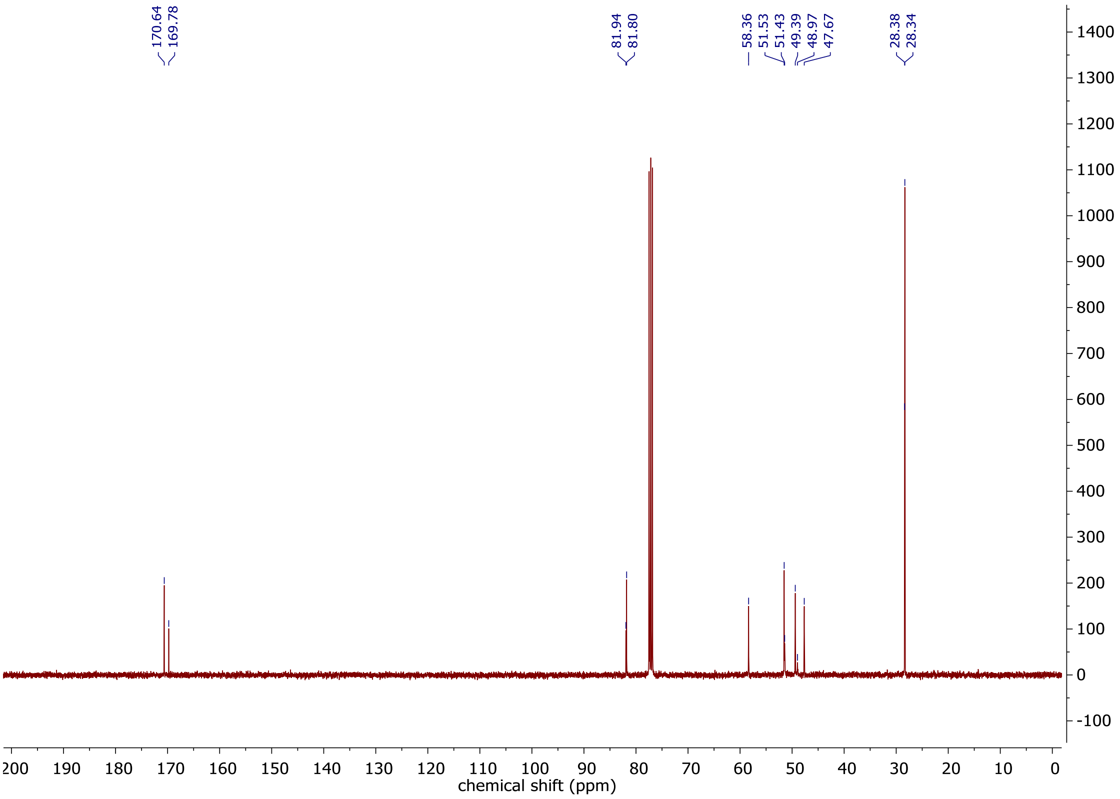

NMR spectra of 7

^{1}H NMR (400 MHz, $CDCl_3$)

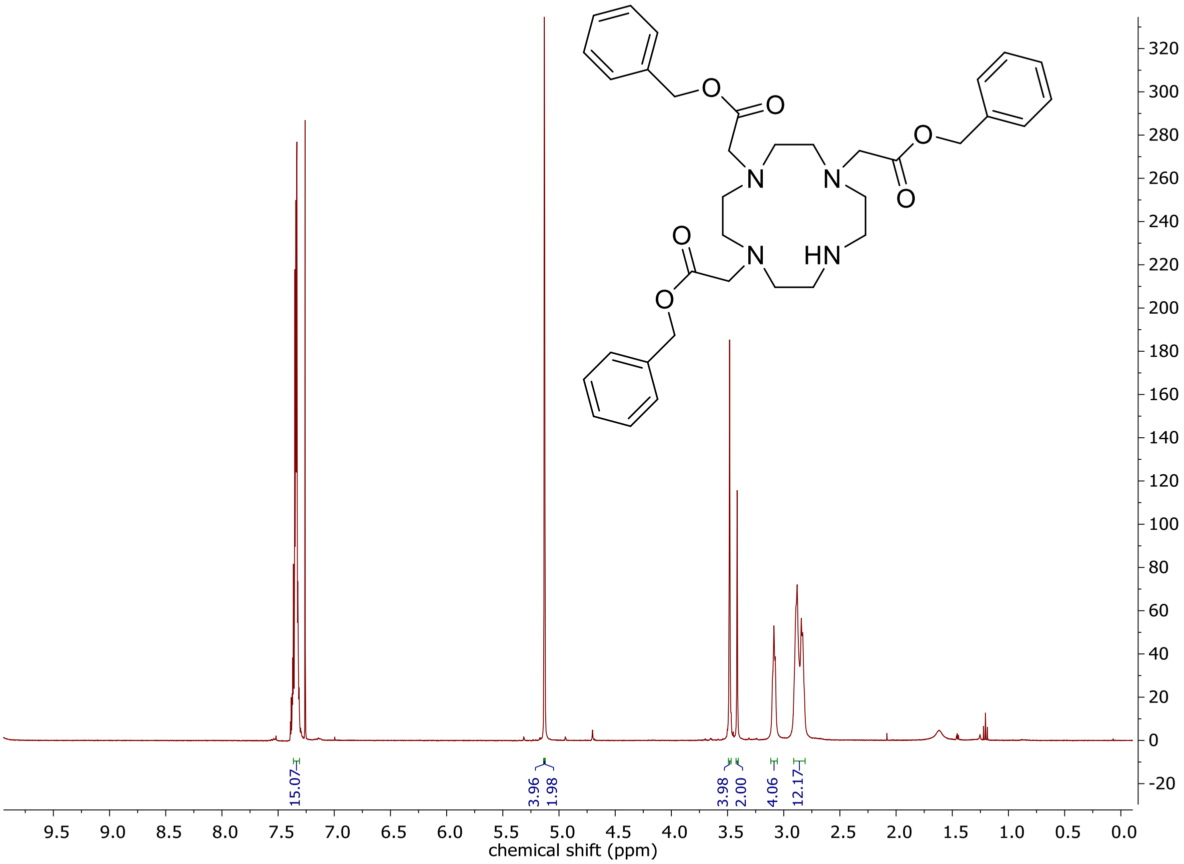

^{13}C NMR (101 MHz, $CDCl_3$)

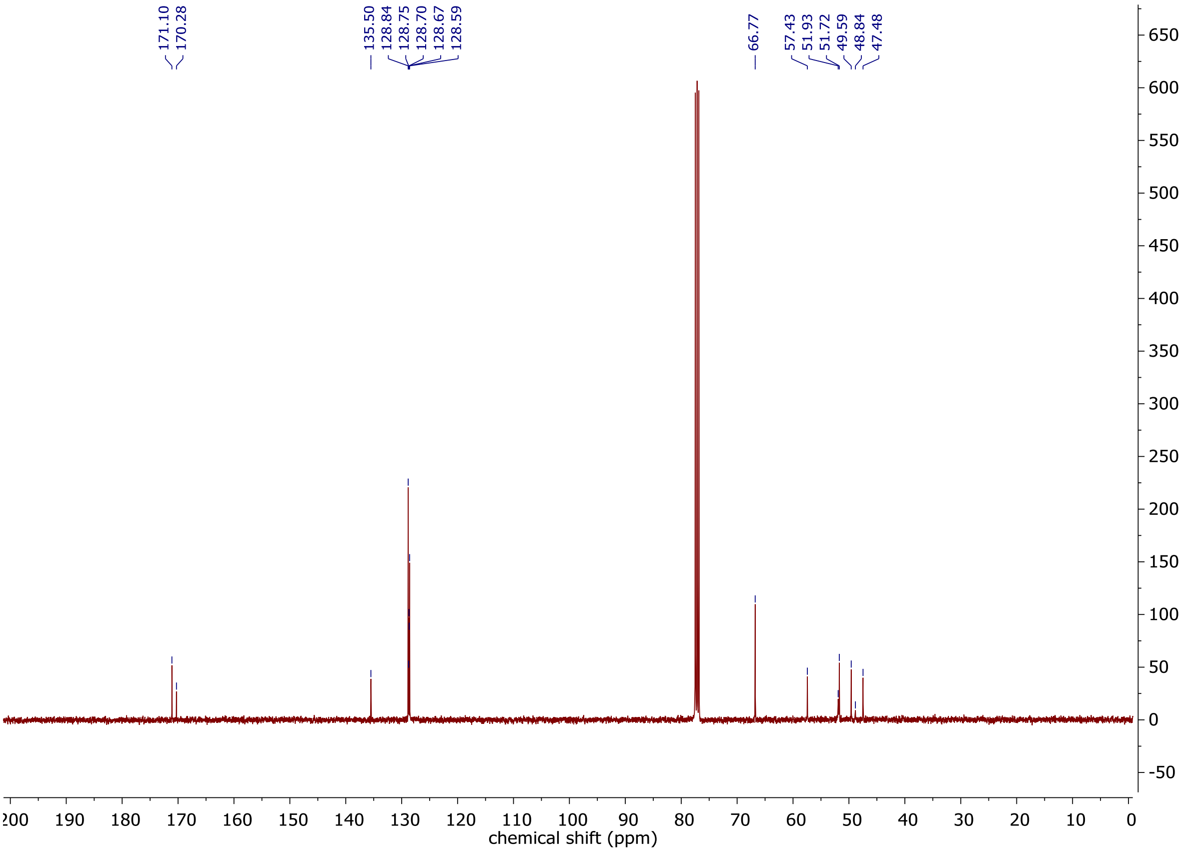

NMR spectra of 8

^{1}H NMR (400 MHz, $CDCl_3$)

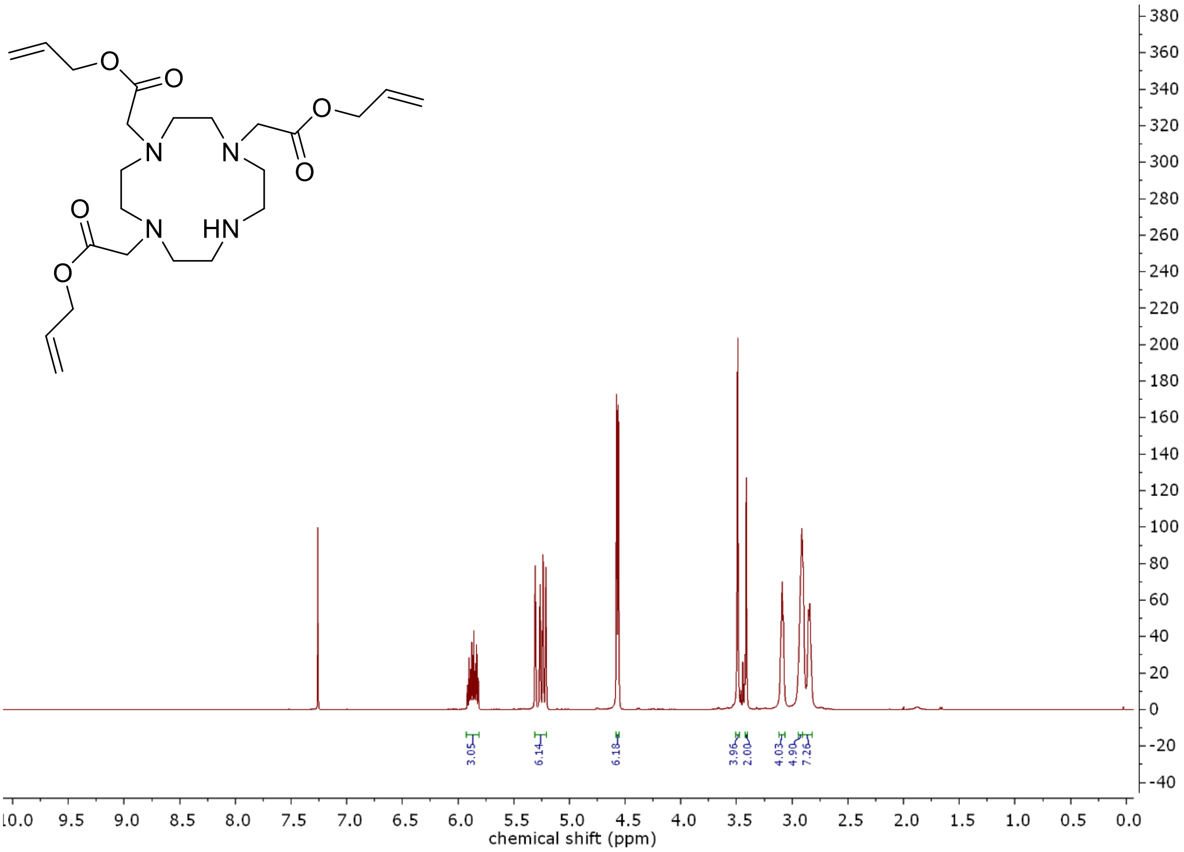

^{13}C APT NMR (101 MHz, $CDCl_3$)

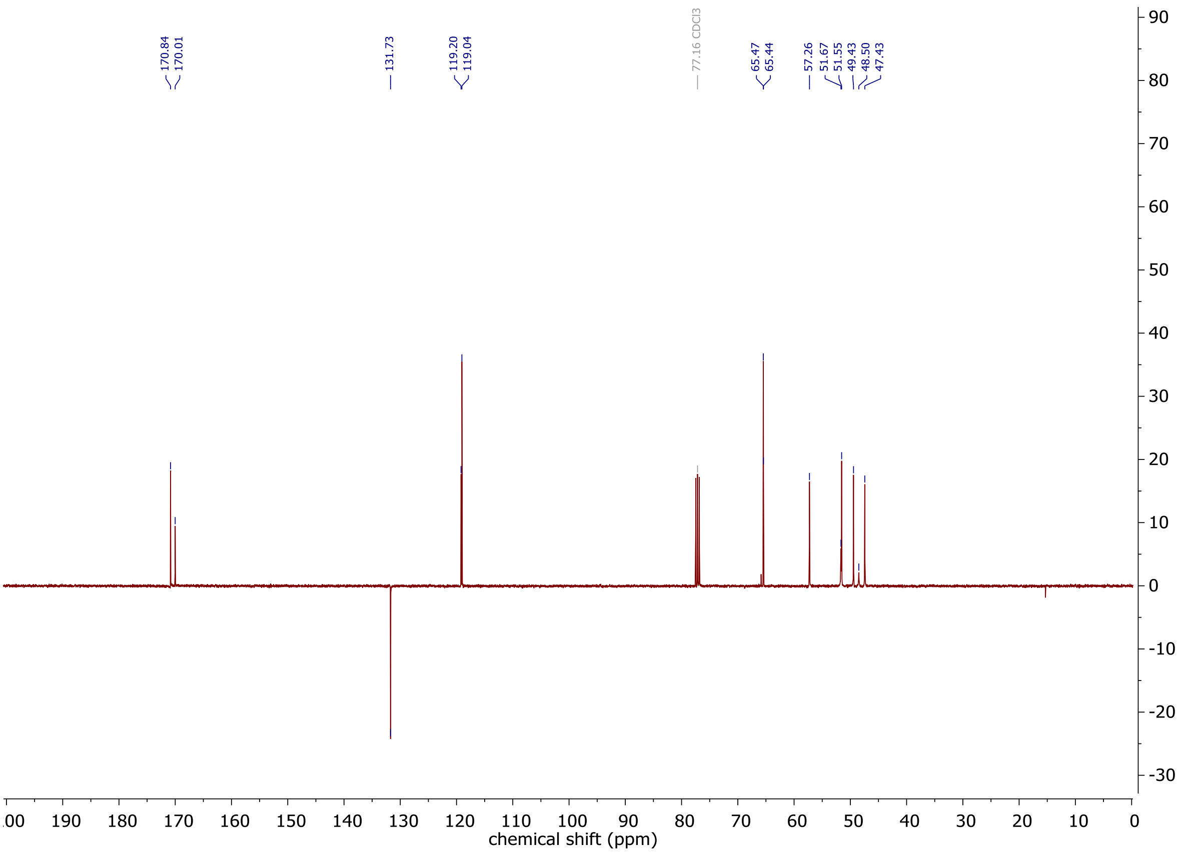

NMR spectra of 11

^{1}H NMR (400 MHz, $CDCl_3$)

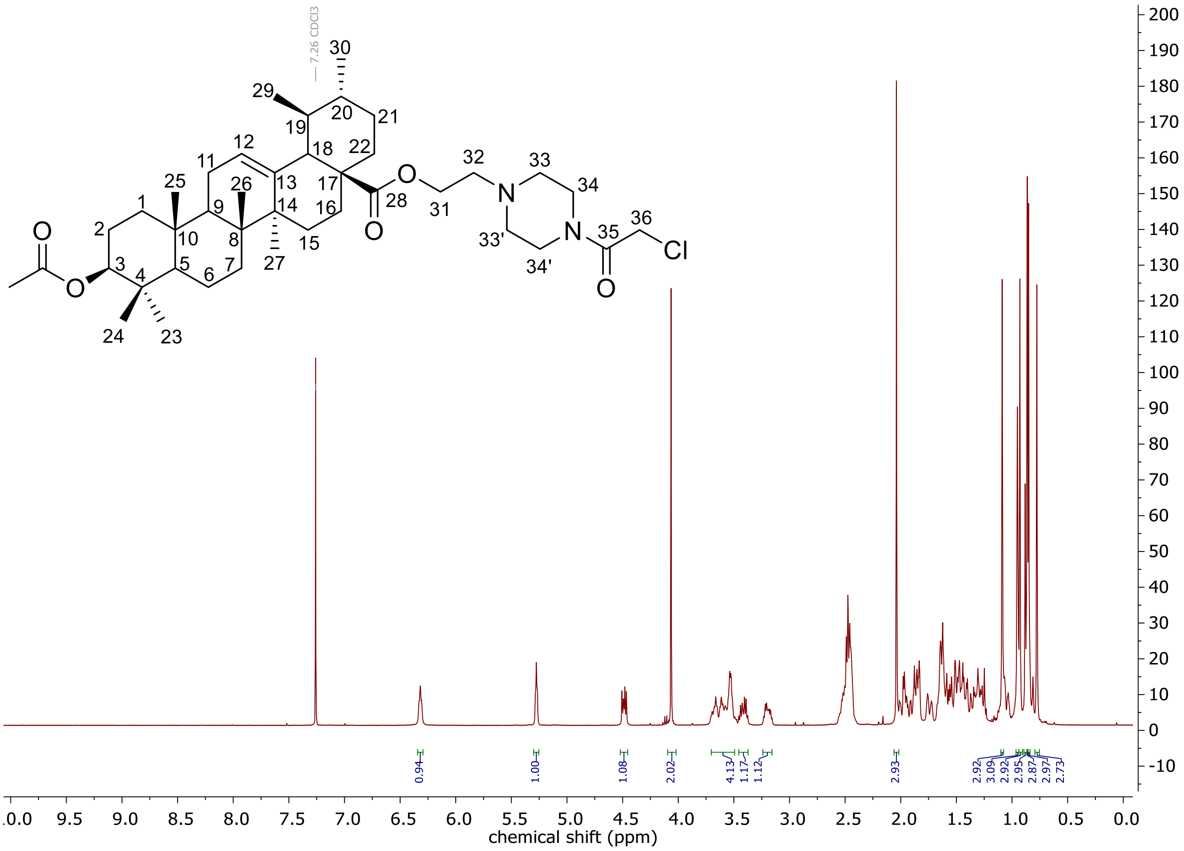

^{13}C APT NMR (101 MHz, $CDCl_3$)

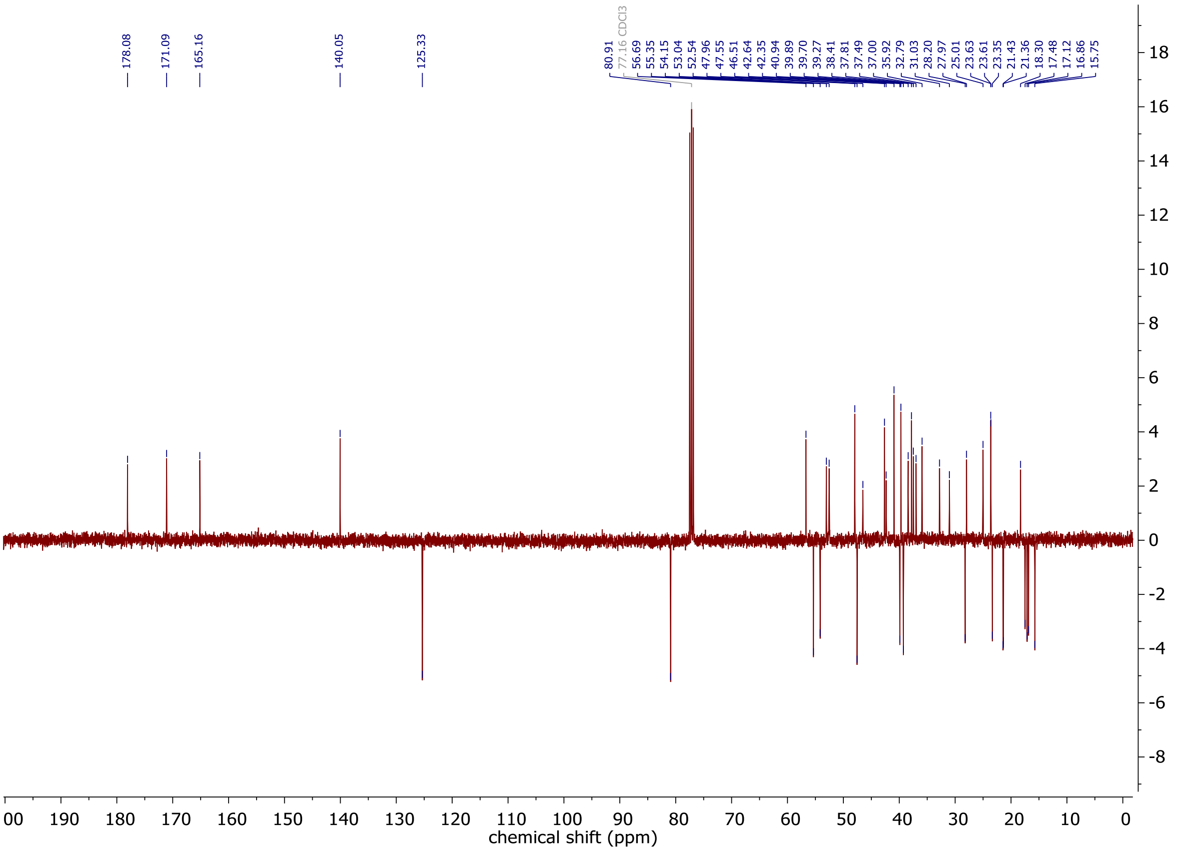

NMR spectra of 12

^{1}H NMR (400 MHz, $CDCl_3$)

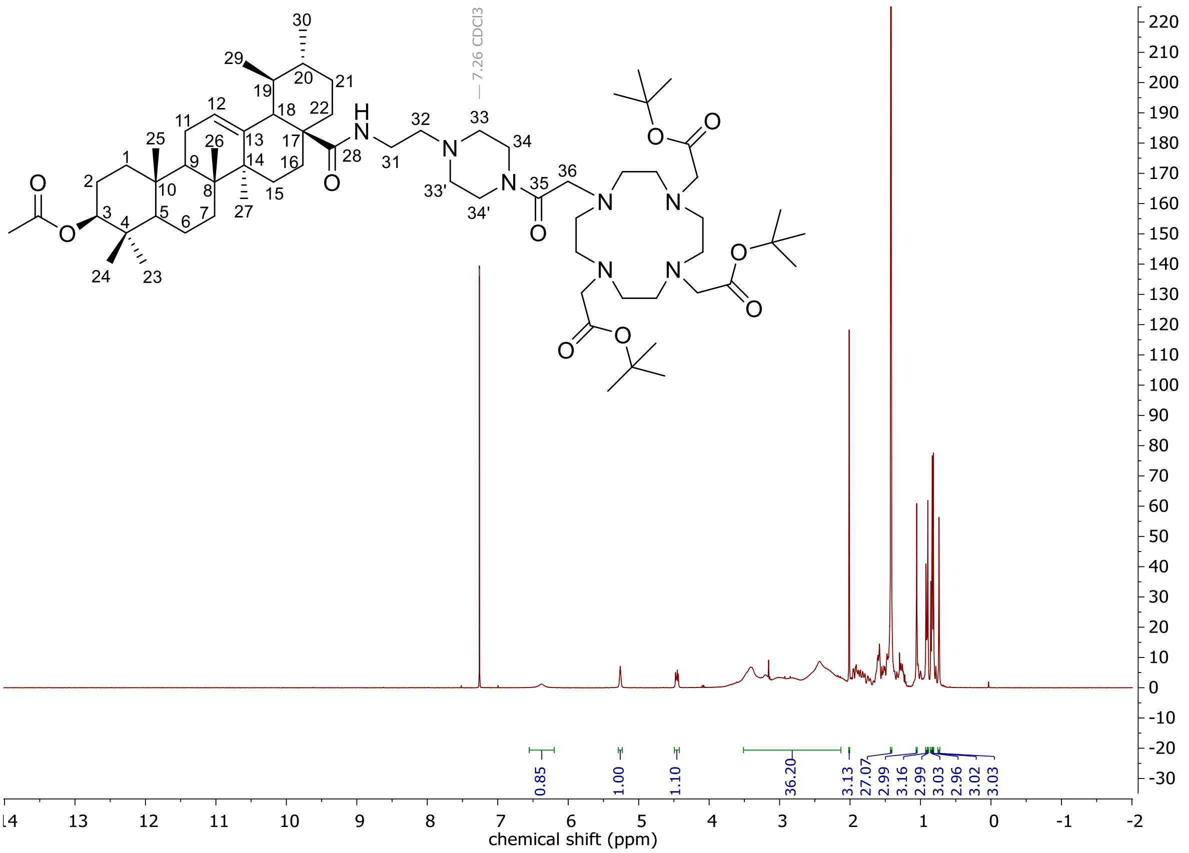

^{13}C NMR (101 MHz, $CDCl_3$)

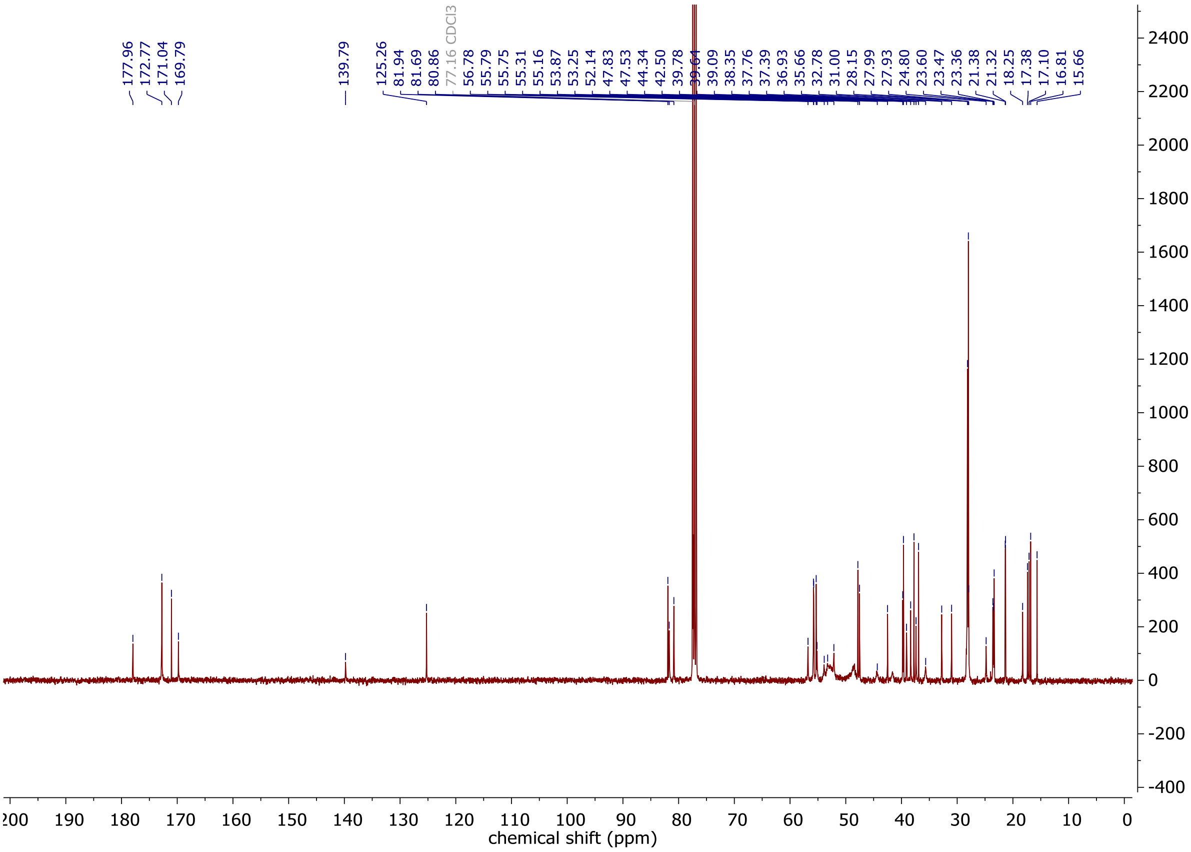

NMR spectra of 13

^{1}H NMR (400 MHz, $CDCl_3$)

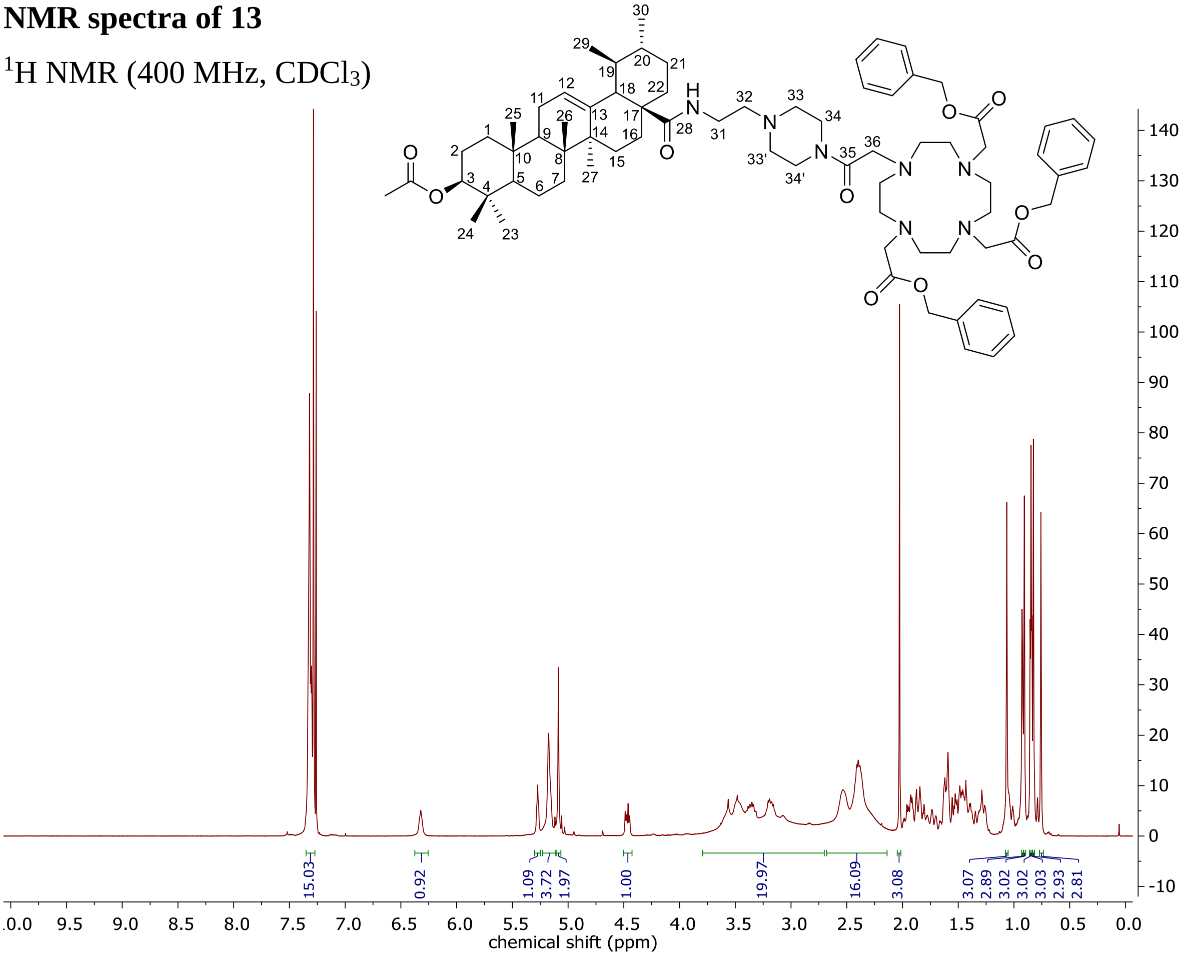

^{13}C APT NMR (101 MHz, $CDCl_3$)

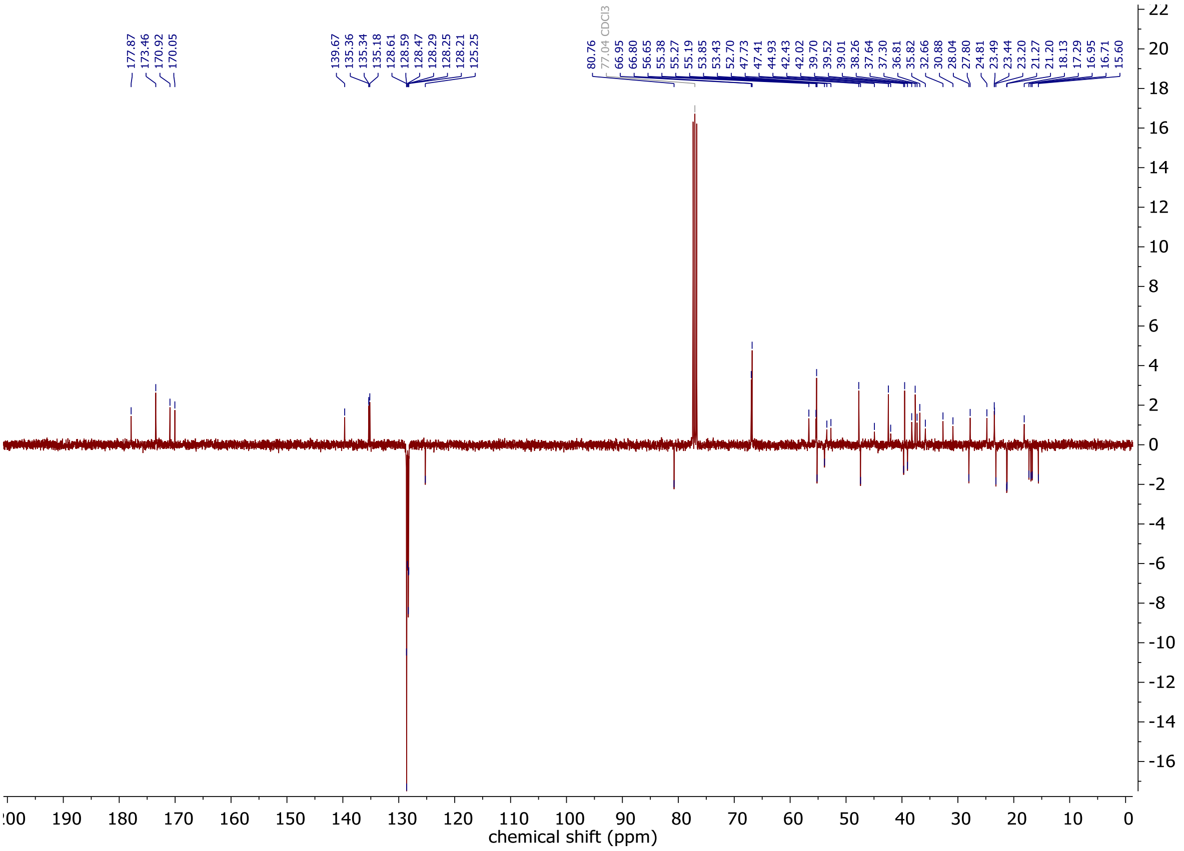

NMR spectra of 14

^{1}H NMR (400 MHz, $CDCl_3$)

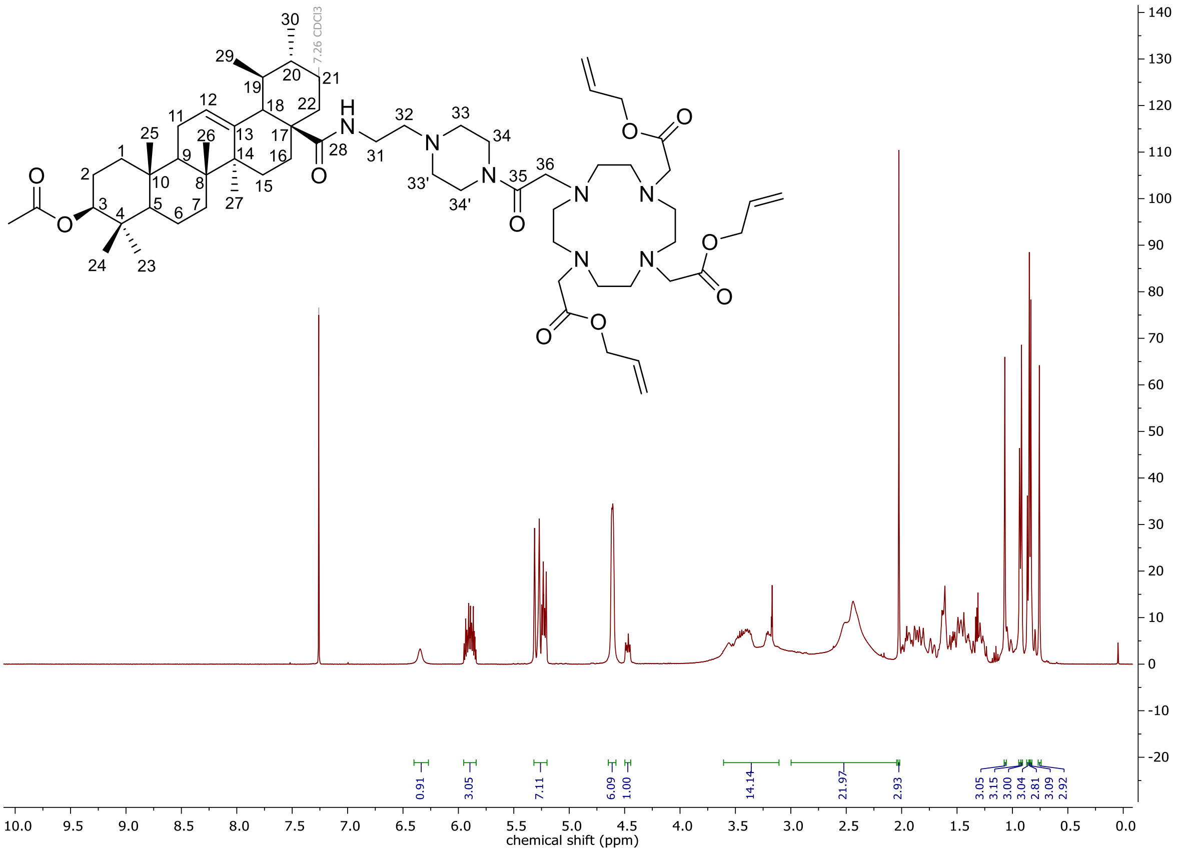

^{13}C APT NMR (101 MHz, $CDCl_3$)

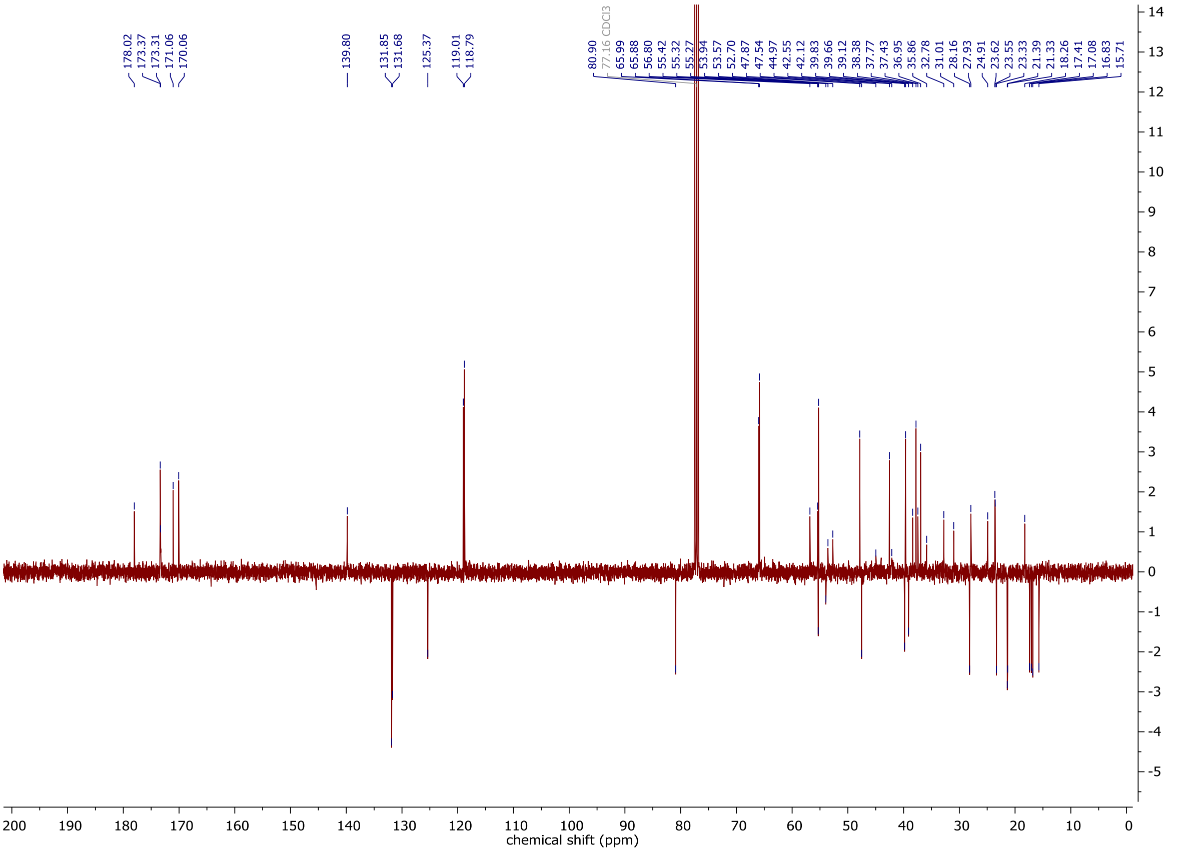

NMR spectra of 15

^{1}H NMR (400 MHz, CD_3OD)

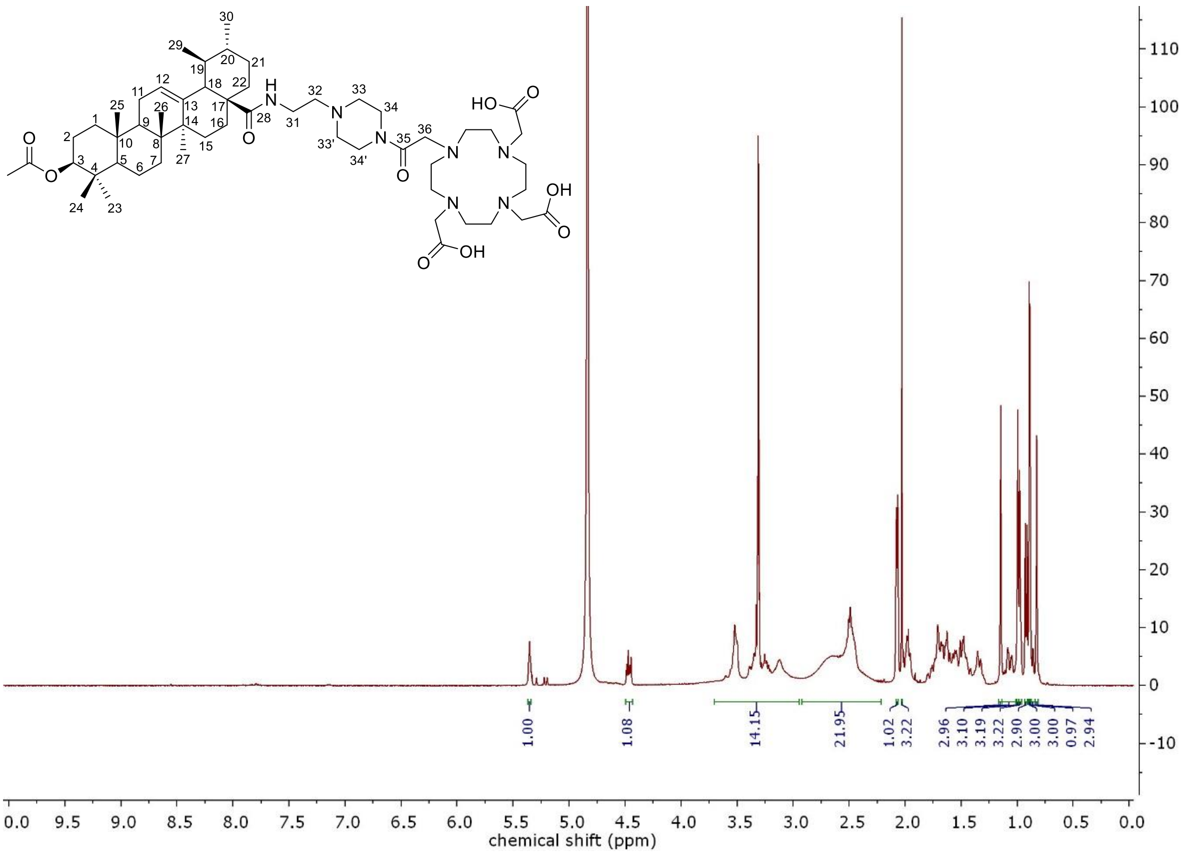

^{13}C APT NMR (101 MHz, CD_3OD)

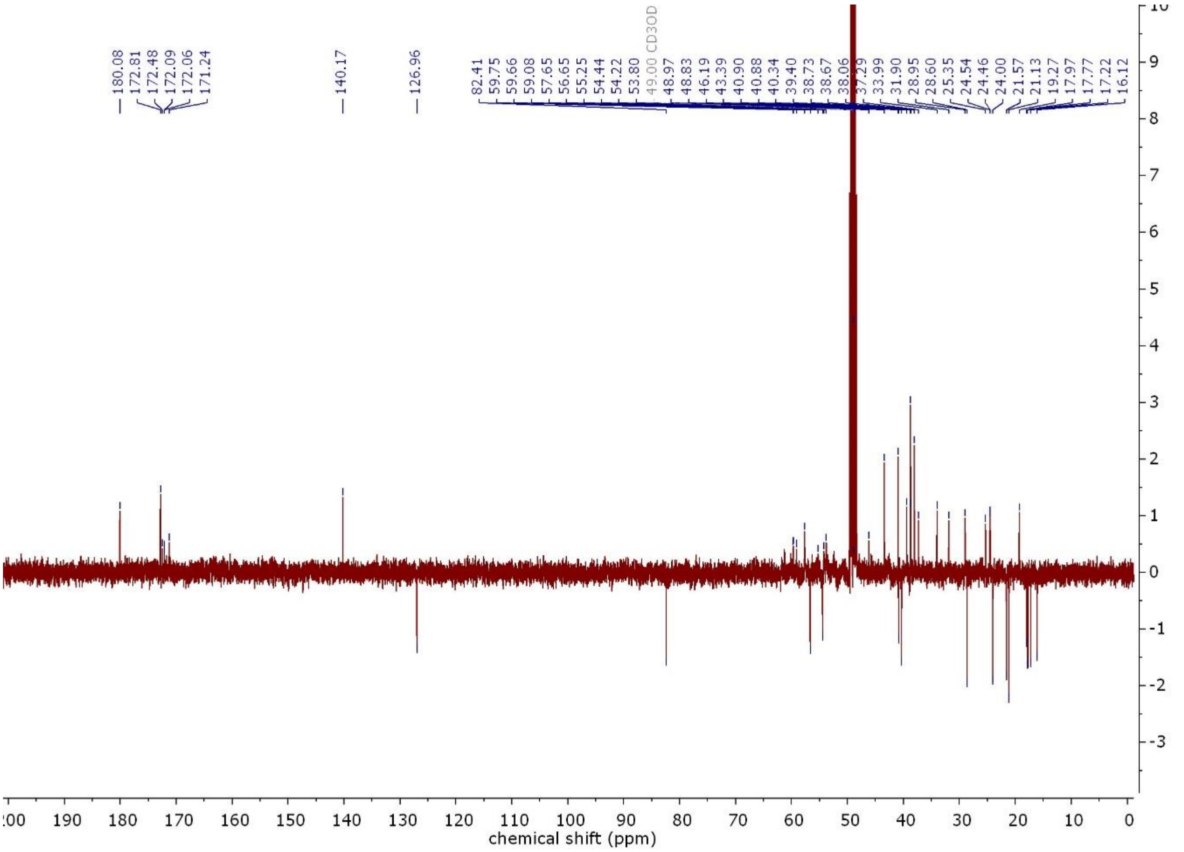

NMR spectra of 19

^{1}H NMR (400 MHz, $CDCl_3$)

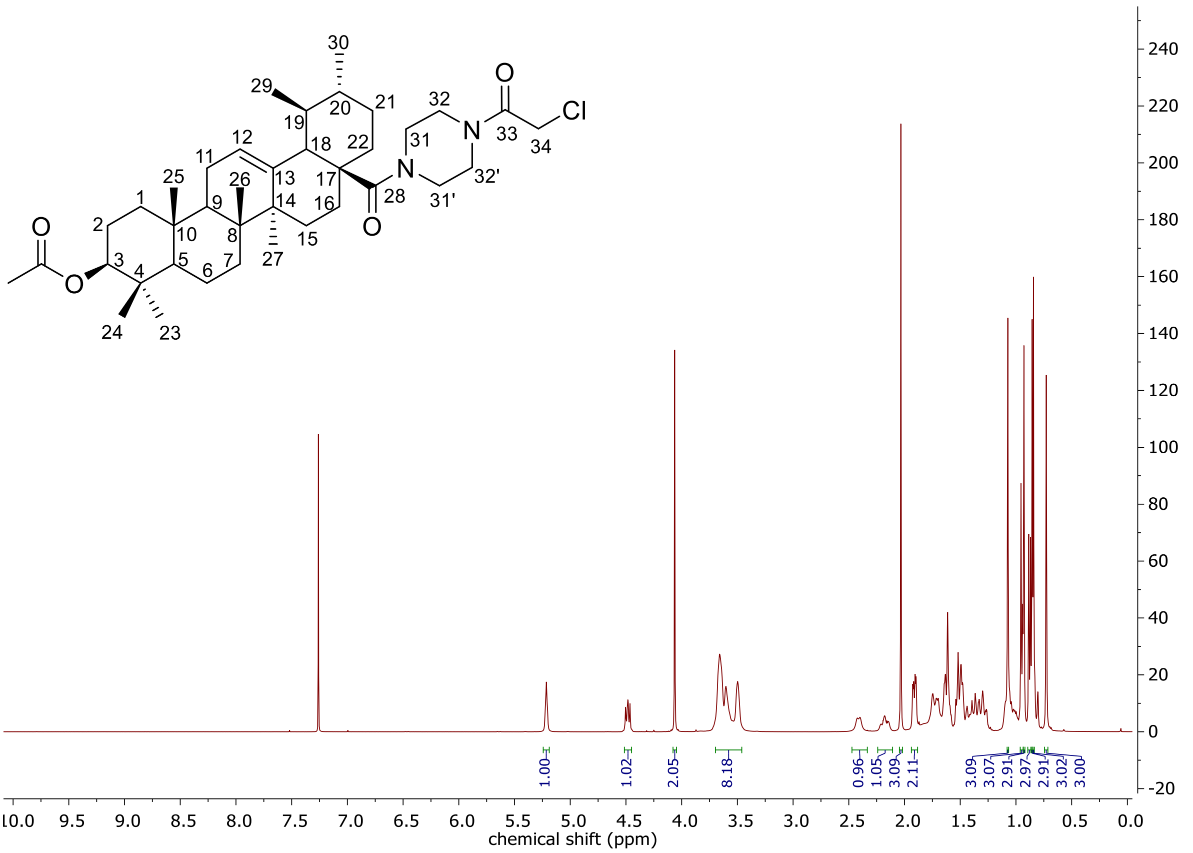

^{13}C APT NMR (101 MHz, $CDCl_3$)

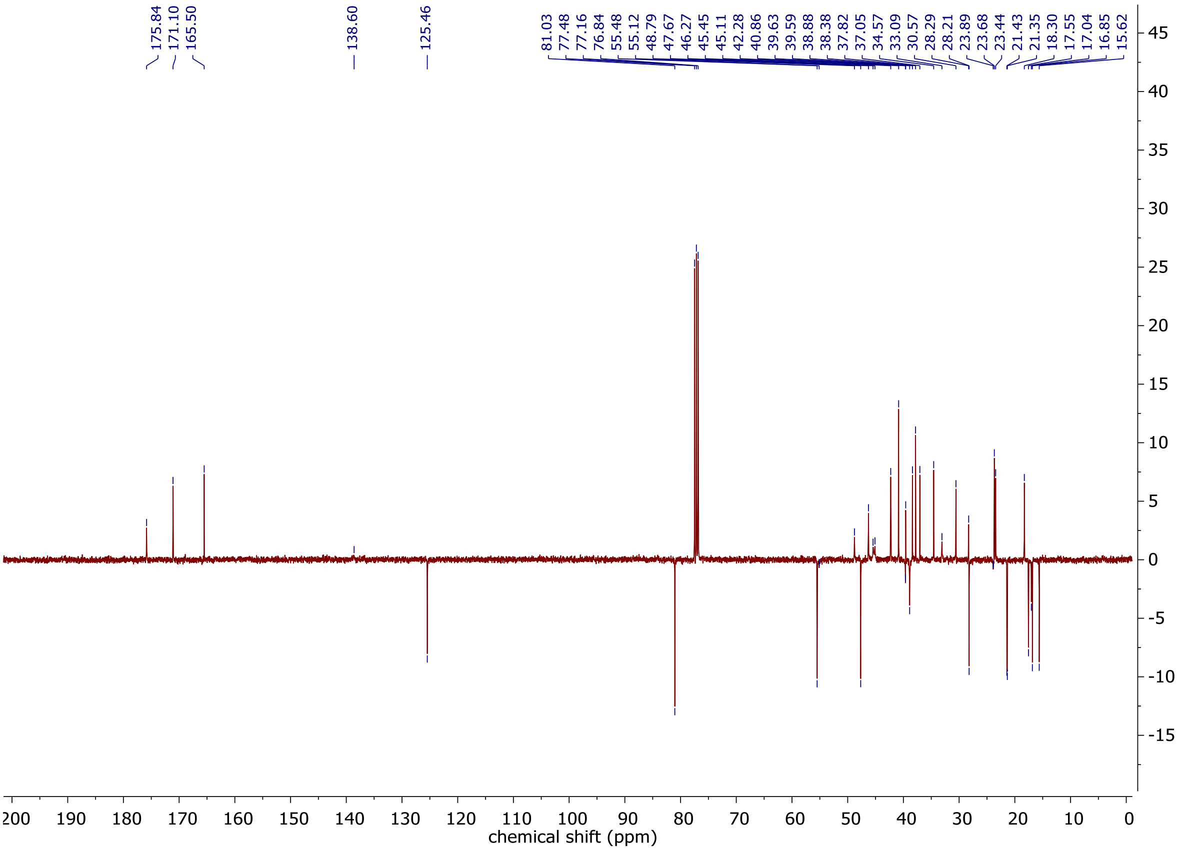

NMR spectra of 20

^{1}H NMR (400 MHz, $CDCl_3$)

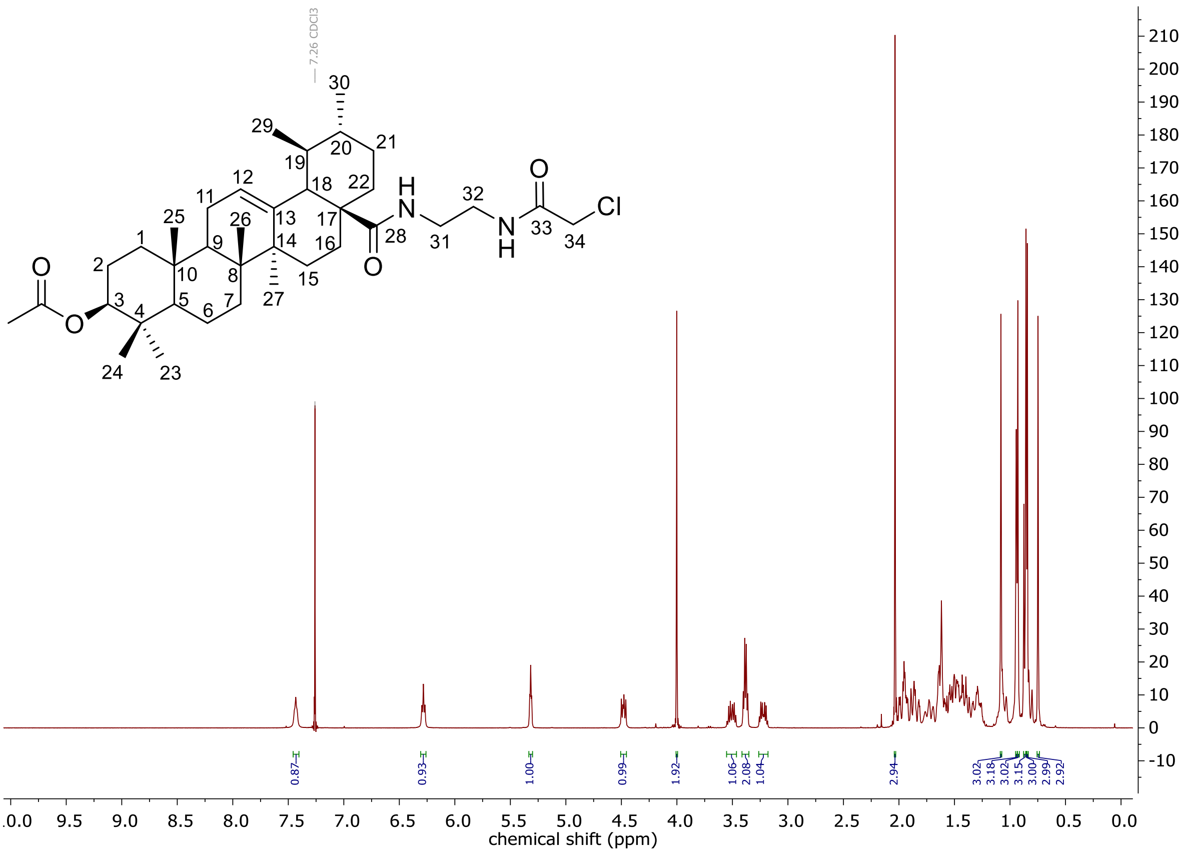

^{13}C APT NMR (101 MHz, $CDCl_3$)

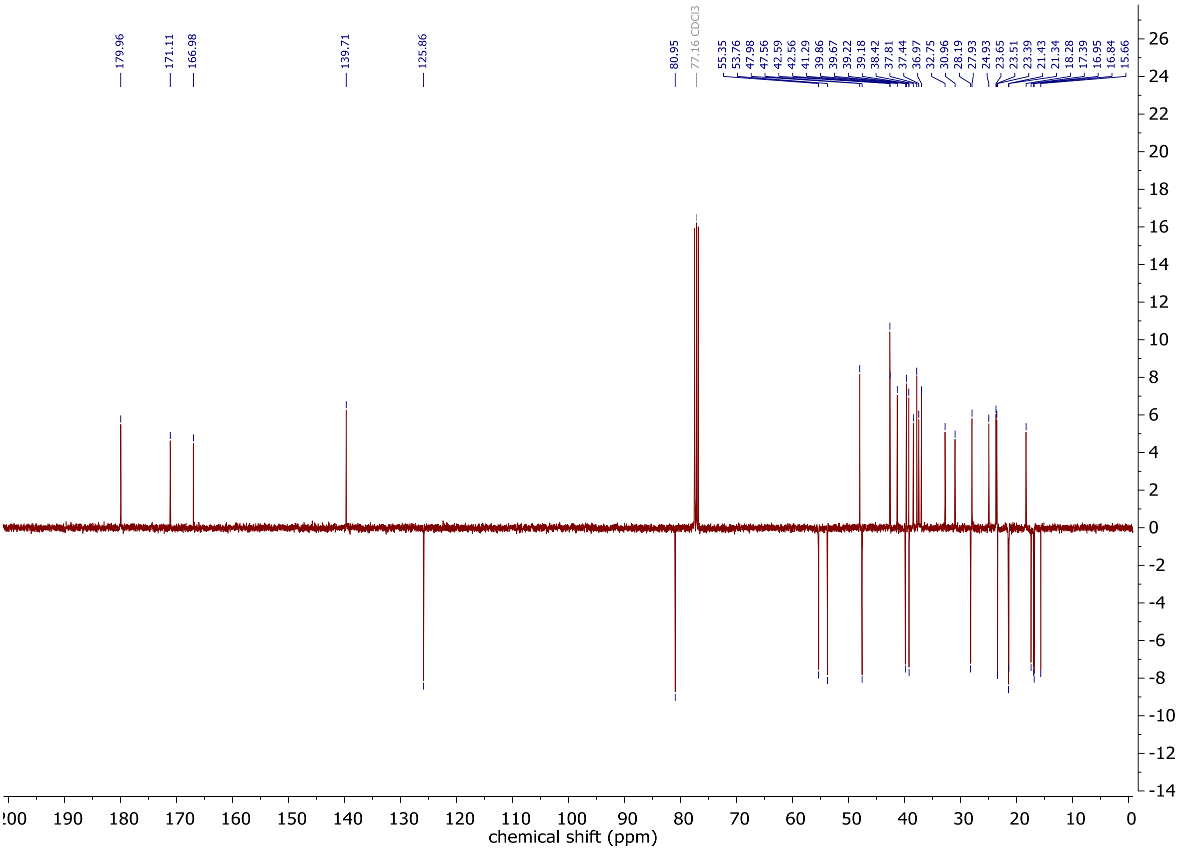

NMR spectra of 21

^{1}H NMR (400 MHz, CDCl$_3$)

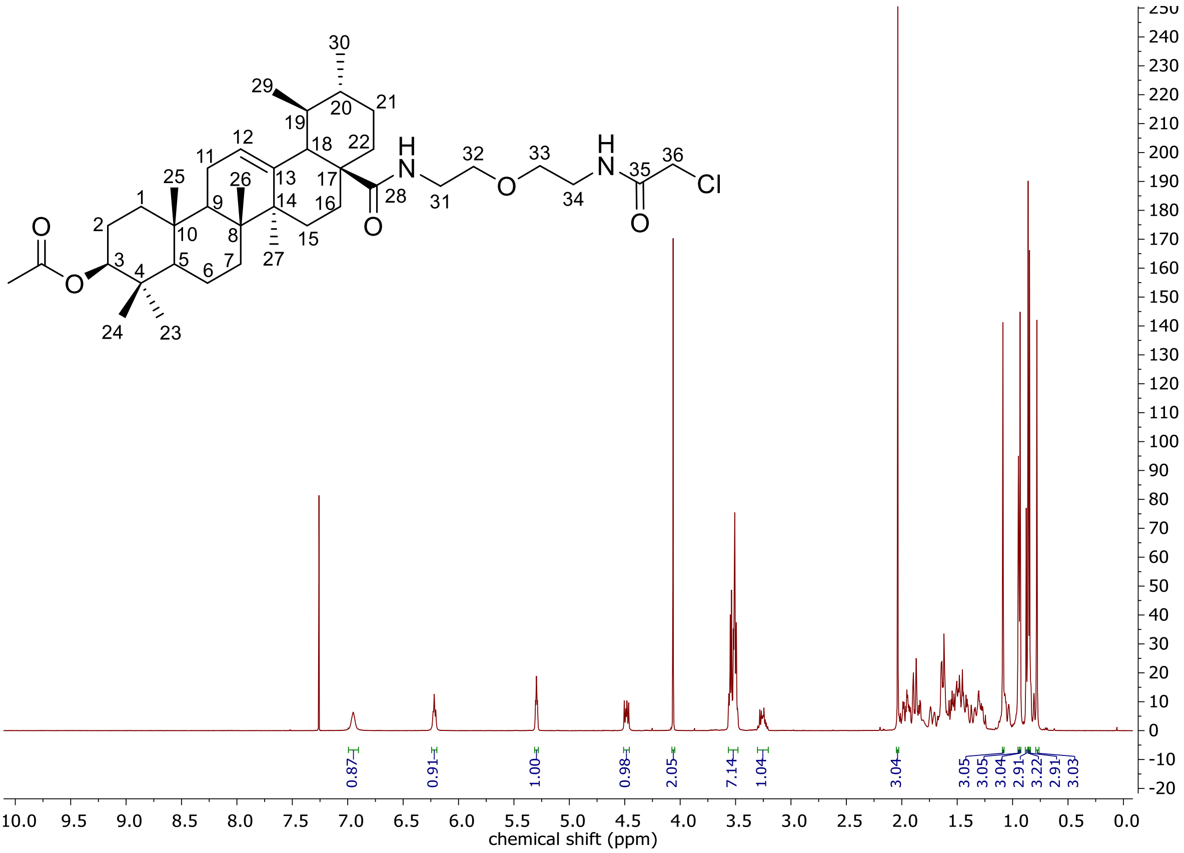

^{13}C APT NMR (101 MHz, CDCl$_3$)

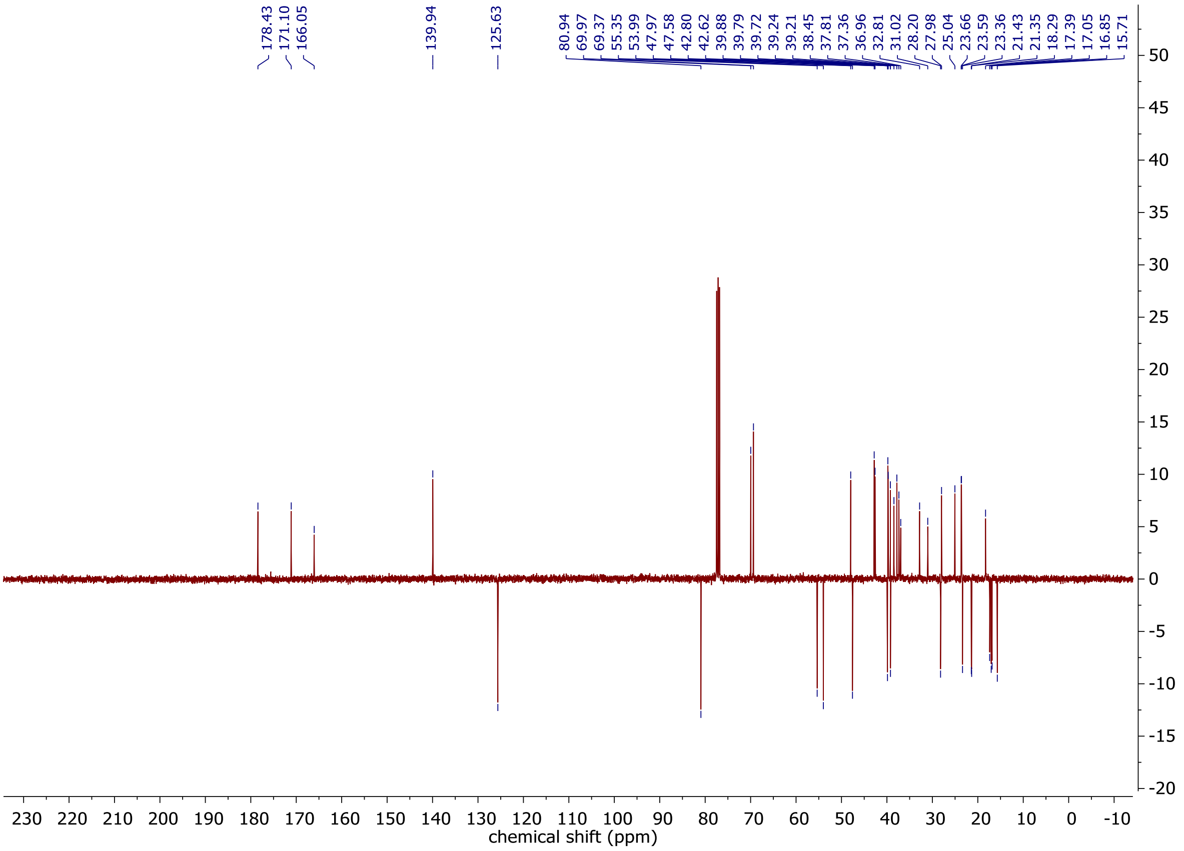

NMR spectra of 22

^{1}H NMR (400 MHz, $CDCl_3$)

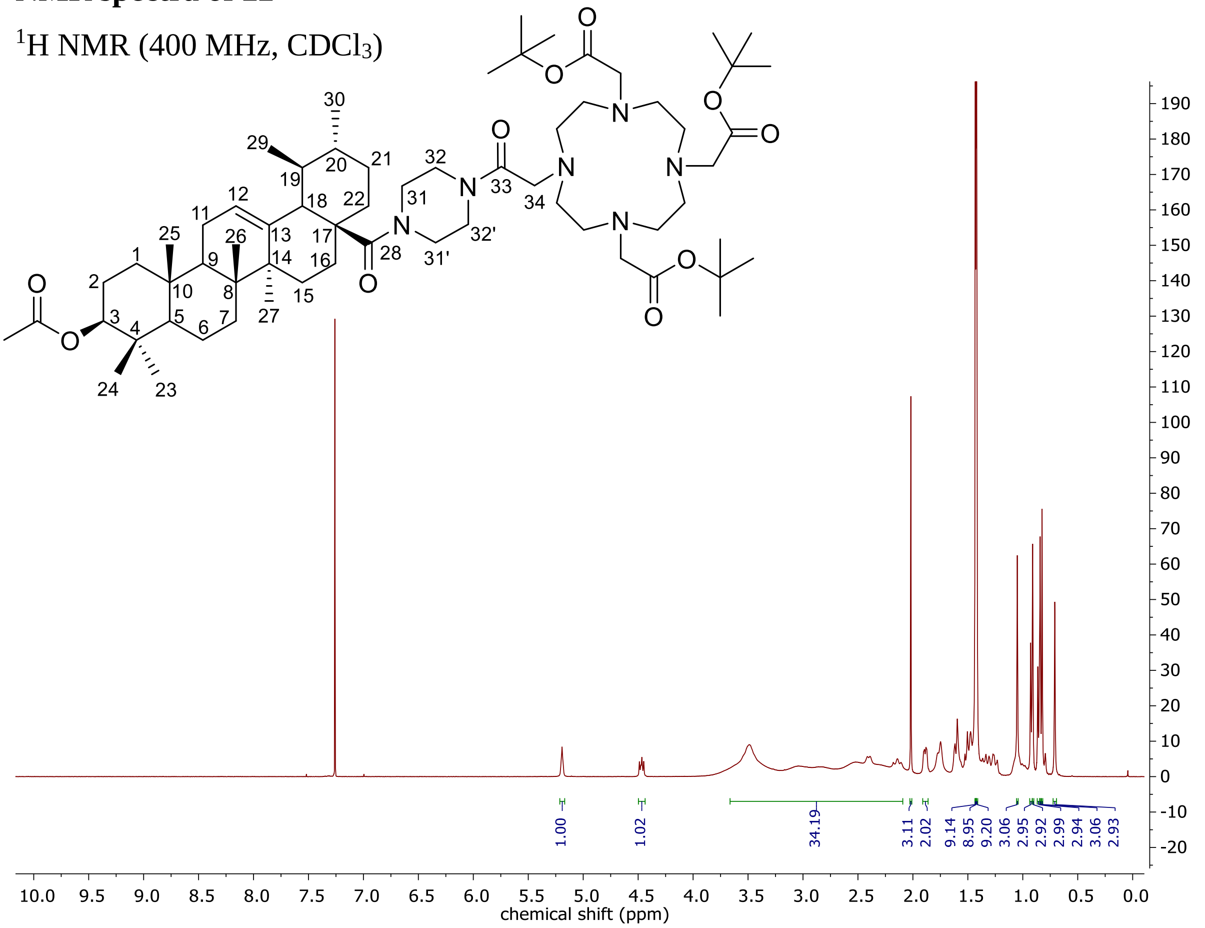

^{13}C NMR (101 MHz, $CDCl_3$)

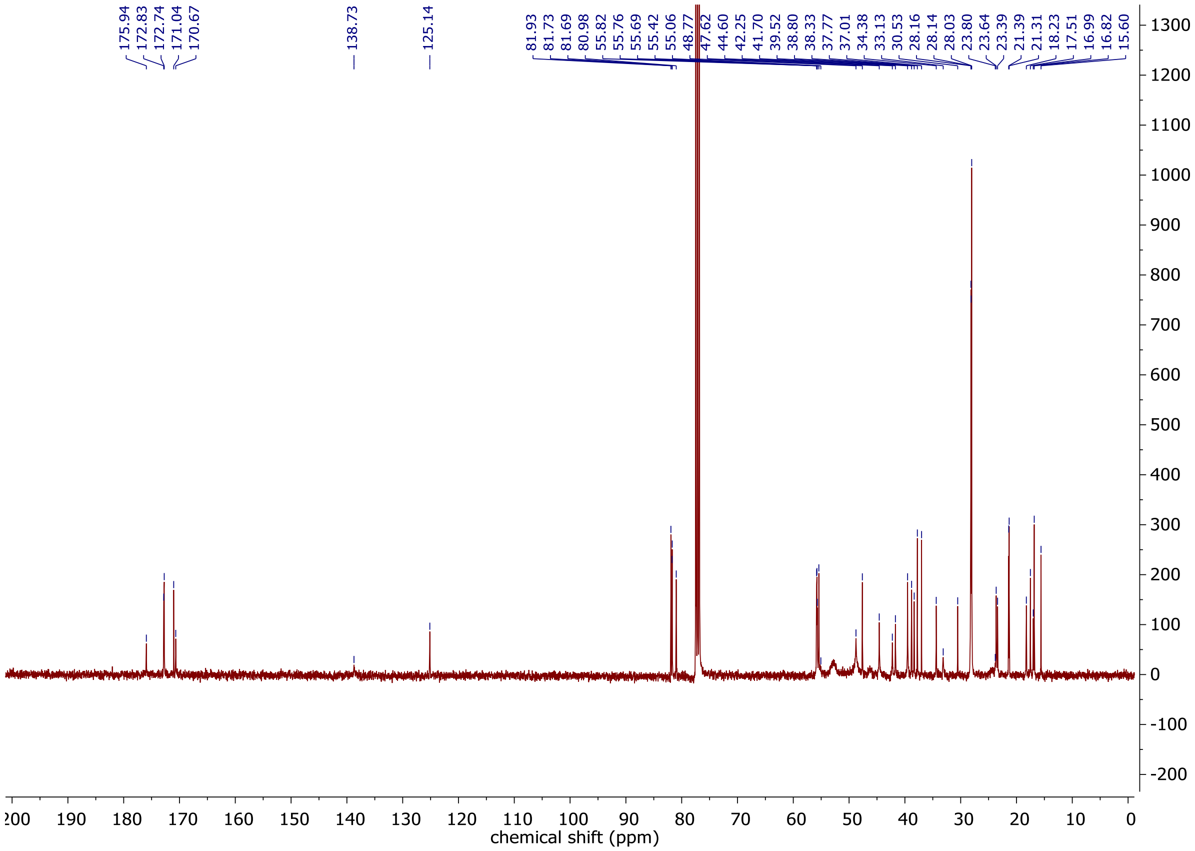

NMR spectra of 23

^{1}H NMR (400 MHz, $CDCl_3$)

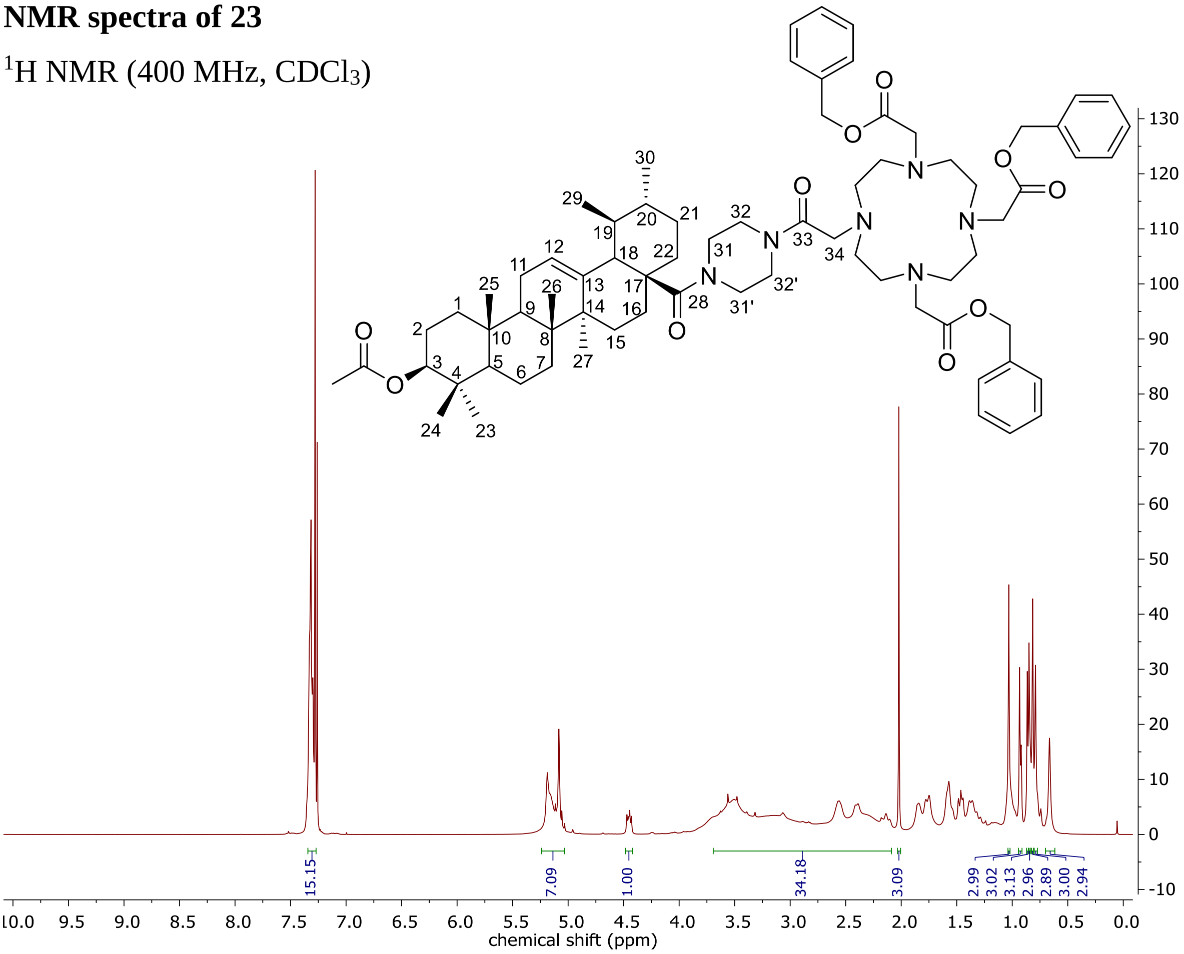

^{13}C NMR (101 MHz, $CDCl_3$)

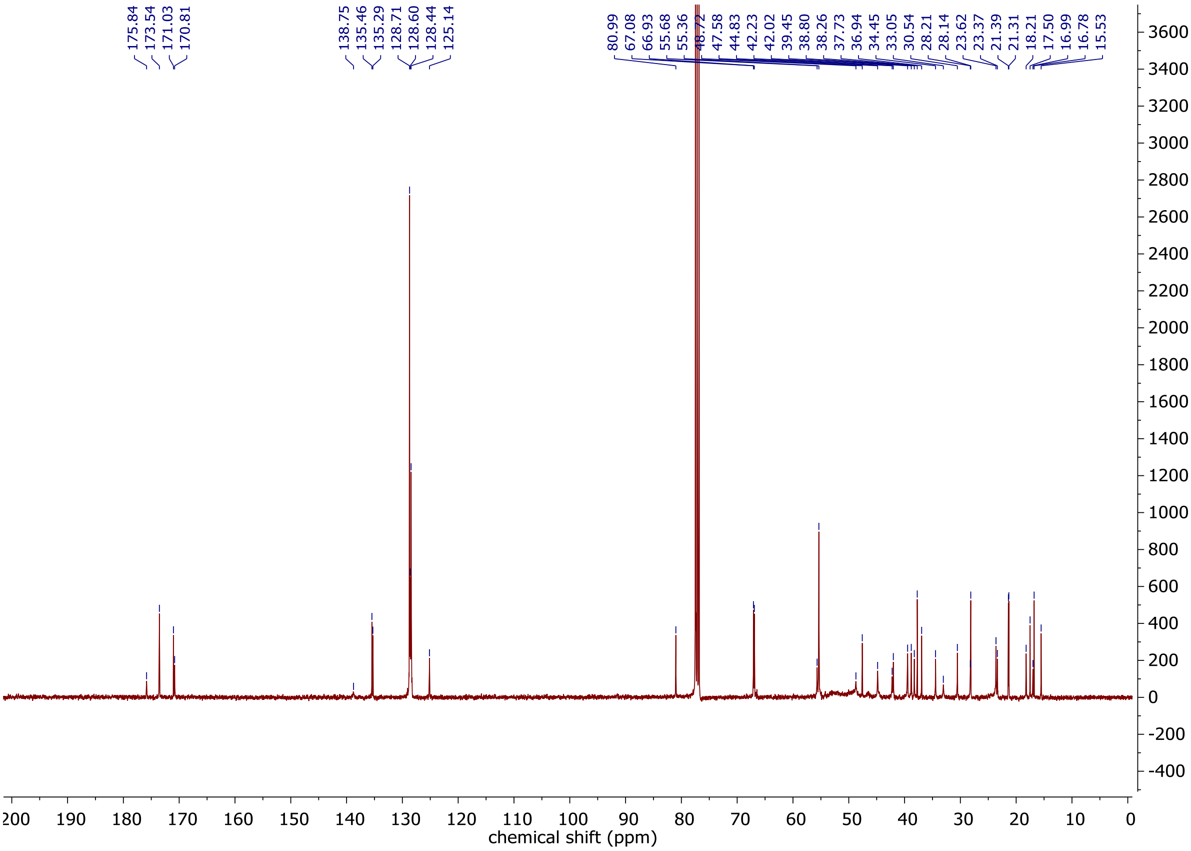

NMR spectra of 24

^{1}H NMR (400 MHz, $CDCl_3$)

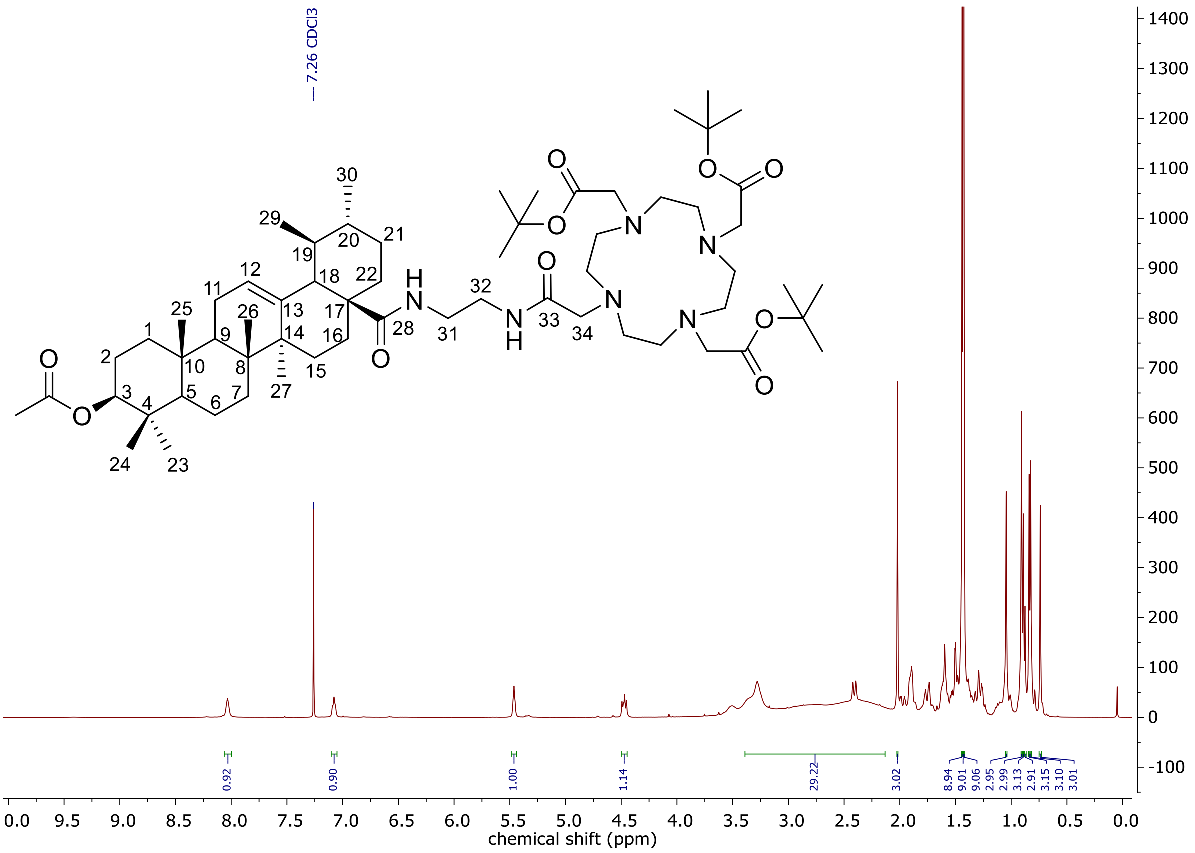

^{13}C NMR (101 MHz, $CDCl_3$)

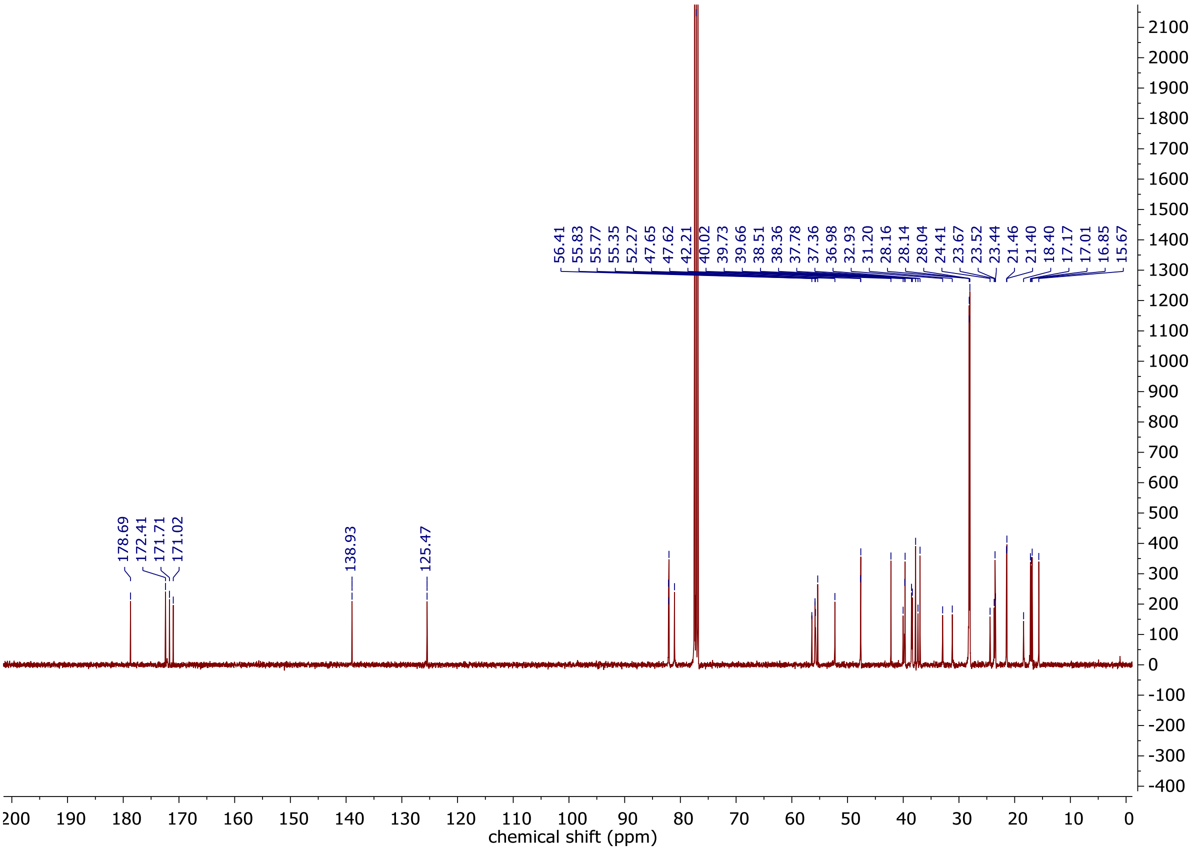

NMR spectra of 25

^{1}H NMR (400 MHz, $CDCl_3$)

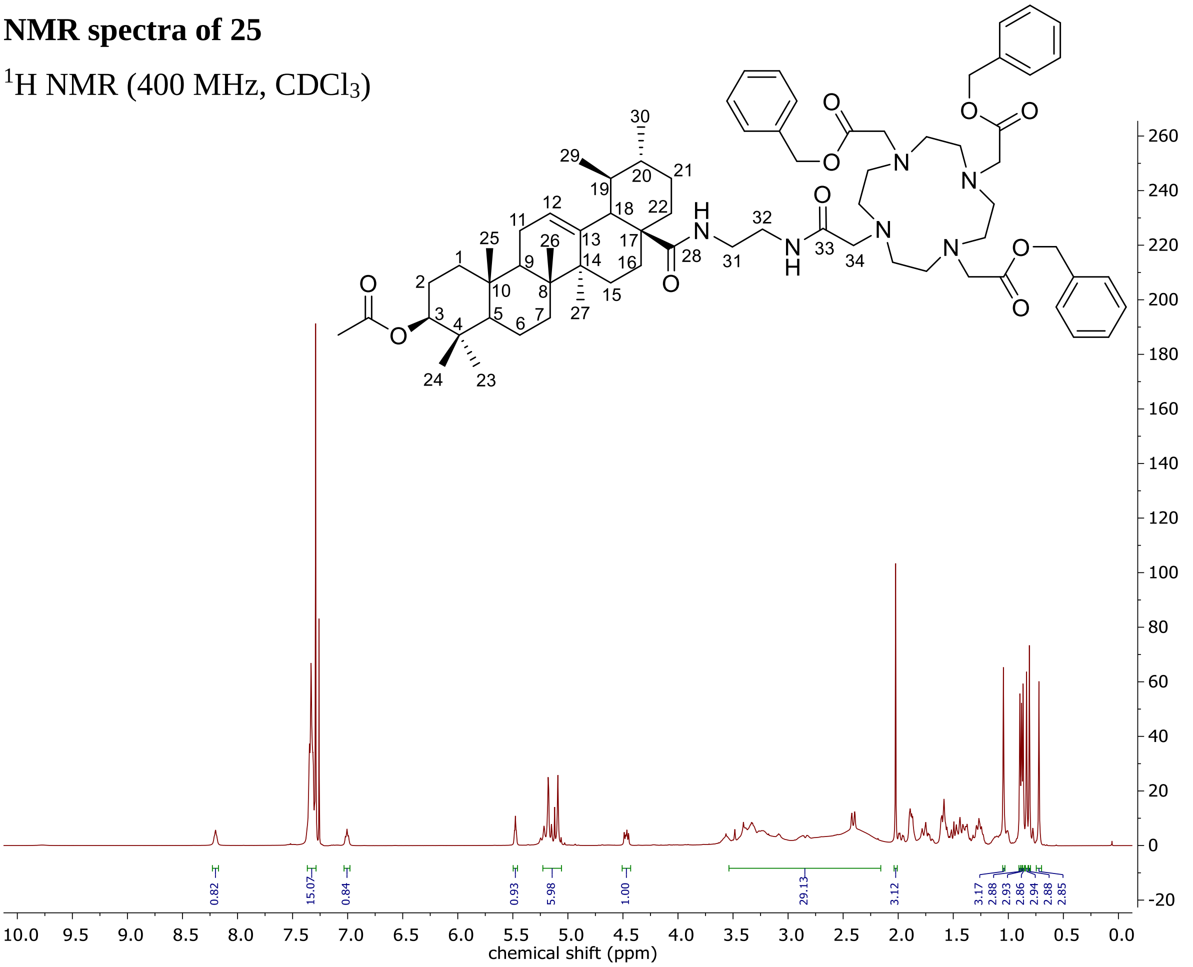

^{13}C NMR (101 MHz, $CDCl_3$)

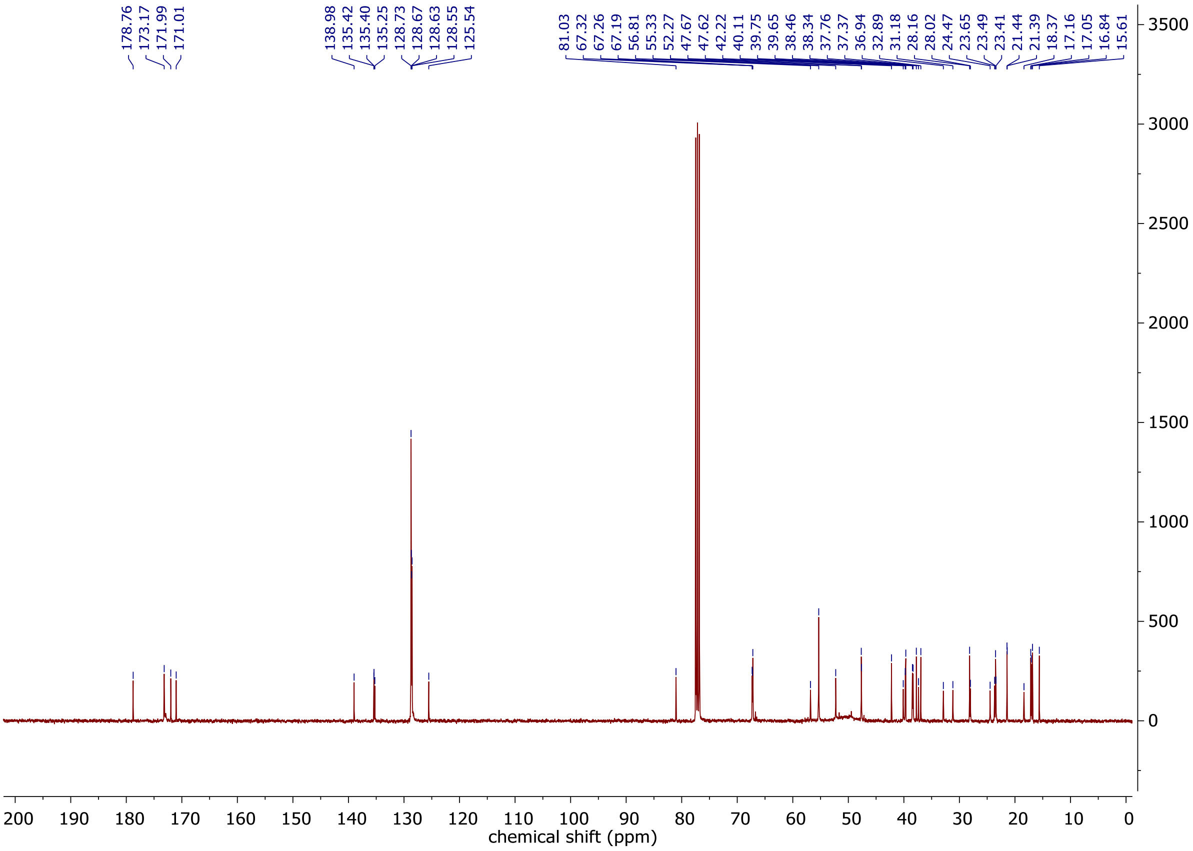

NMR spectra of 26

^{1}H NMR (400 MHz, $CDCl_3$)

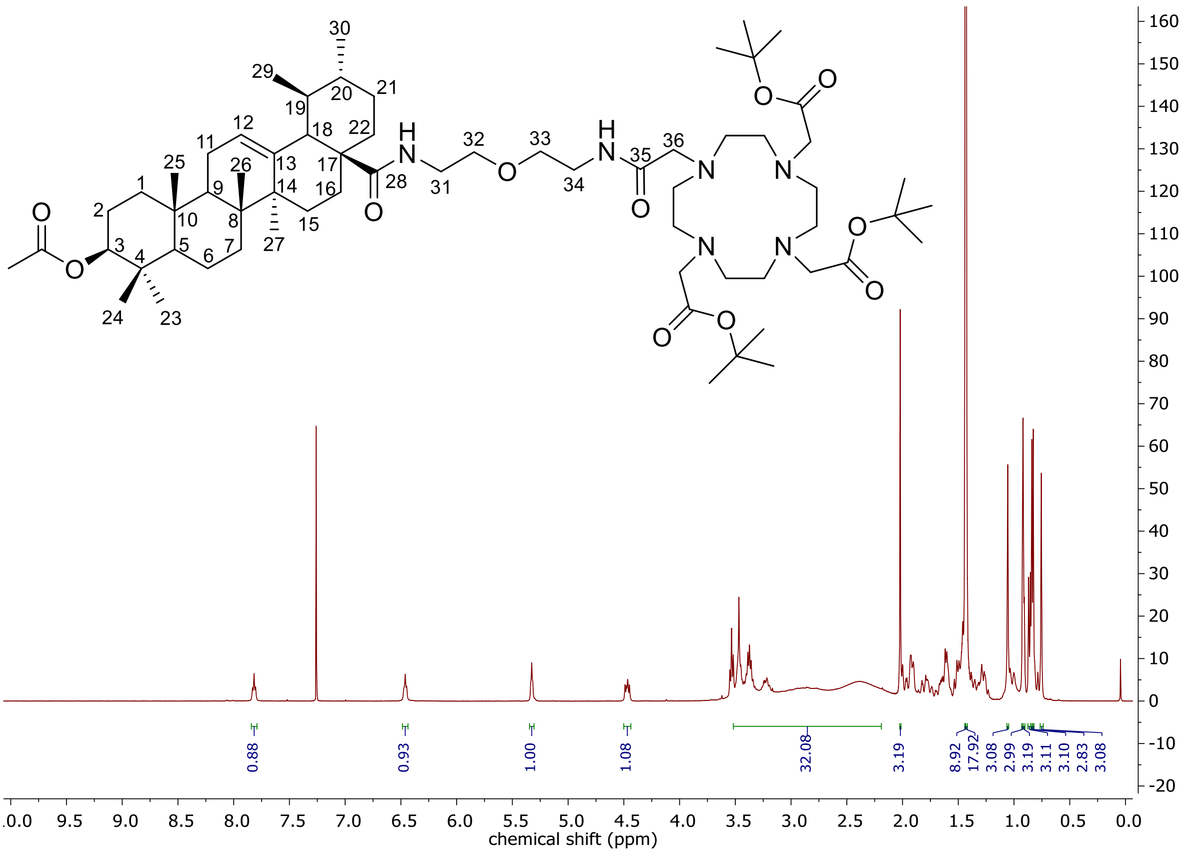

^{13}C APT NMR (101 MHz, $CDCl_3$)

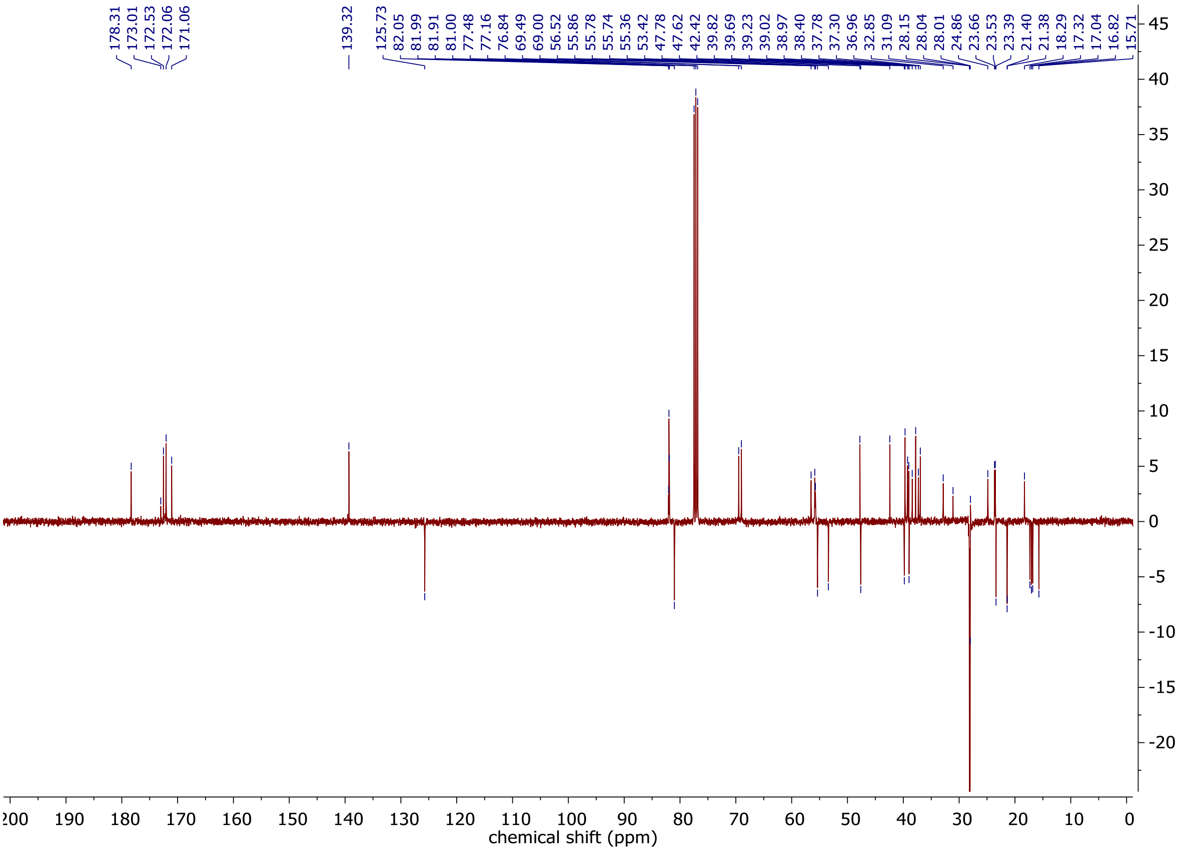

NMR spectra of 27

^{1}H NMR (400 MHz, $CDCl_3$)

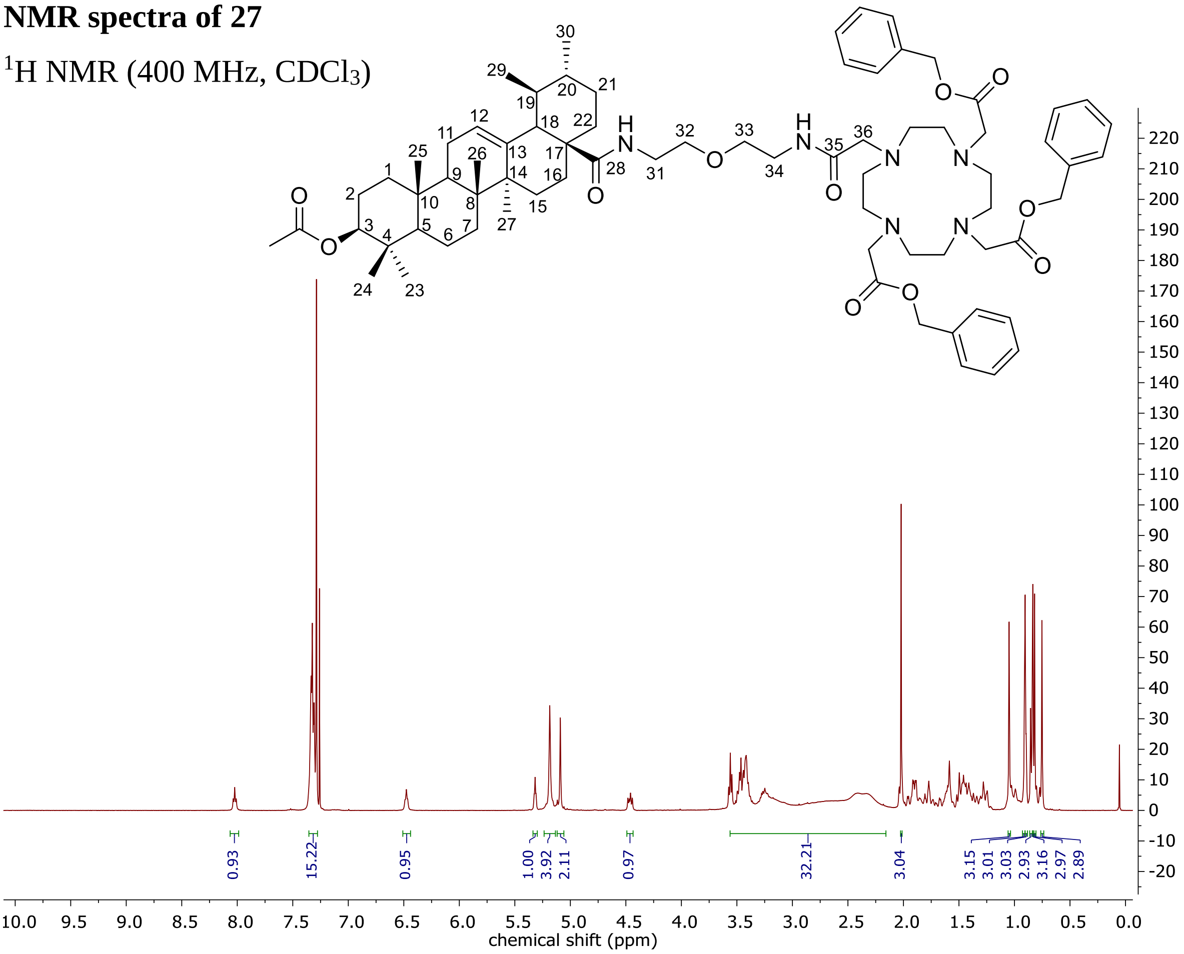

^{13}C APT NMR (101 MHz, $CDCl_3$)

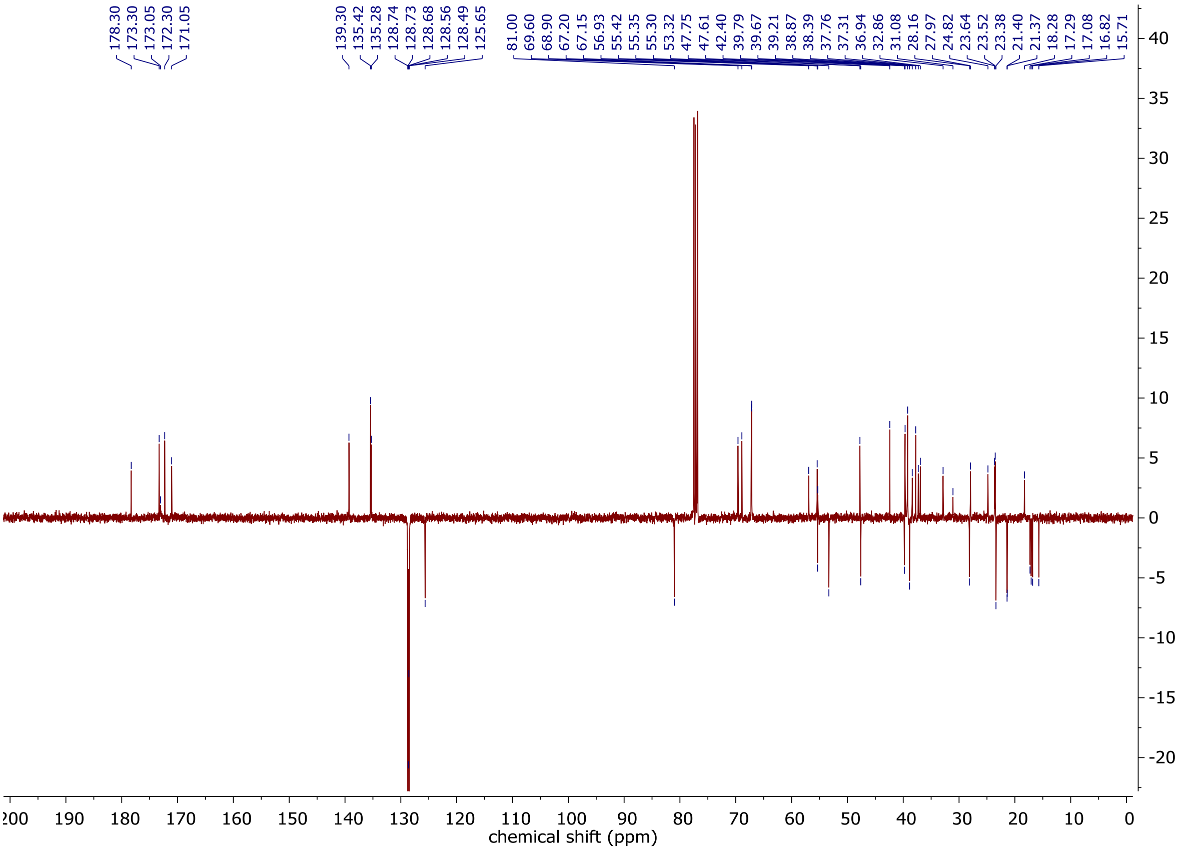

NMR spectra of 29

^{1}H NMR (400 MHz, $CDCl_3$)

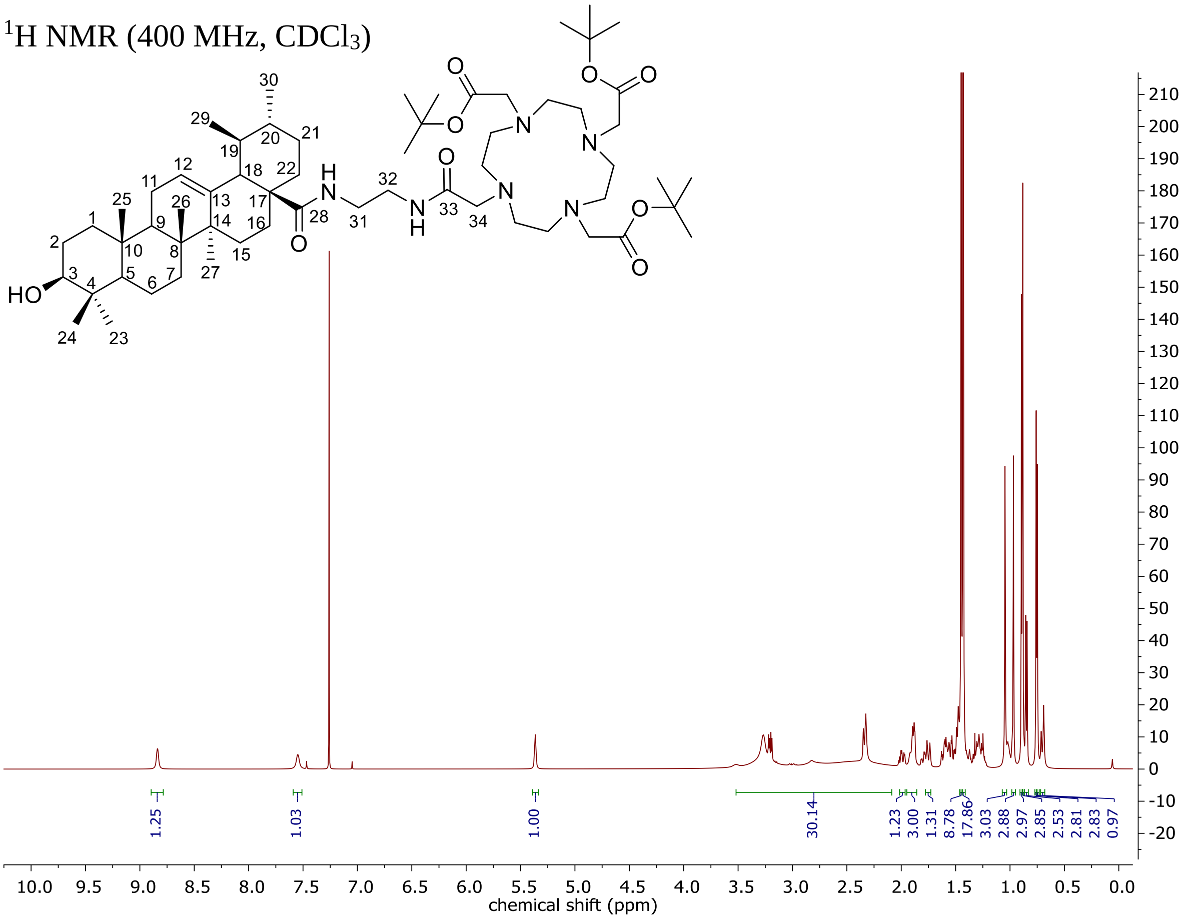

^{13}C APT NMR (101 MHz, $CDCl_3$)

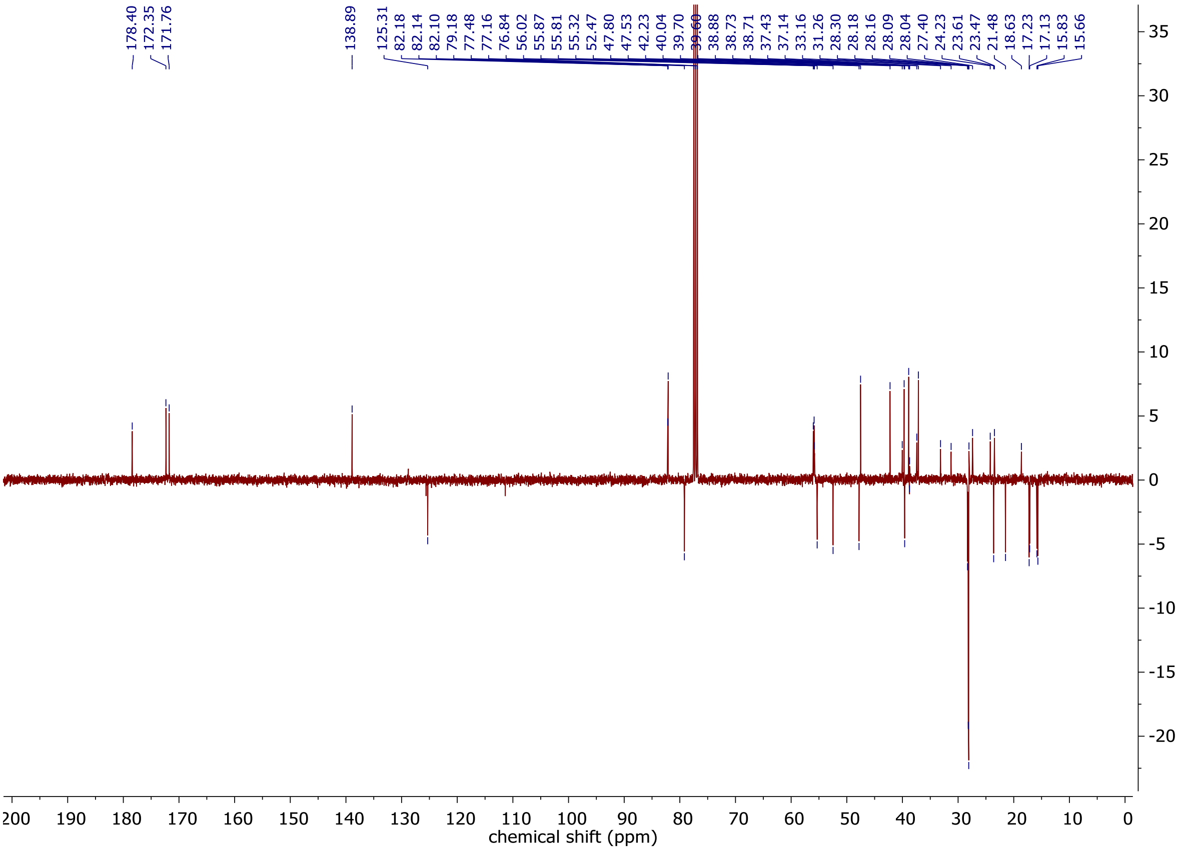

6. Calculation of ADMET parameters

(SwissADME: a free web tool to evaluate pharmacokinetics, drug-likeness and medicinal chemistry friendliness of small molecules. *Sci. Rep.* (2017) 7:42717.)

Compound 22:

Molecule 1

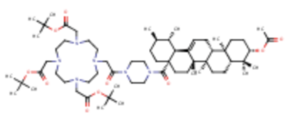

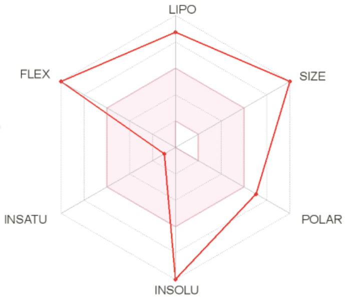

SMILES: CC(=O)O[C@H]1CC[C@]2([C@H](C1(C)C)CC[C@@]1([C@@H]2CC=C2[C@@]1(C)CC[C@@]1([C@@H]2[C@@H](C)[C@H](C)CC1)C(=O)N1CCN(CC1)C(=O)CN1CCN(CCN(CCN(CC1)CC(=O)OC(C)(C)C)CC(=O)OC(C)(C)C)CC(=O)OC(C)(C)C)C)C

Physicochemical Properties	
Formula	C64H108N6O10
Molecular weight	1121.58 g/mol
Num. heavy atoms	80
Num. arom. heavy atoms	0
Fraction Csp3	0.88
Num. rotatable bonds	19
Num. H-bond acceptors	14
Num. H-bond donors	0
Molar Refractivity	339.71
TPSA	158.78 Å²

Lipophilicity	
Log $P_{o/w}$ (iLOGP)	8.58
Log $P_{o/w}$ (XLOGP3)	9.79
Log $P_{o/w}$ (WLOGP)	6.21
Log $P_{o/w}$ (MLOGP)	4.04
Log $P_{o/w}$ (SILICOS-IT)	7.55
Consensus Log $P_{o/w}$	7.23

Water Solubility	
Log *S* (ESOL)	-11.71
Solubility	2.20e-09 mg/ml ; 1.96e-12 mol/l
Class	Insoluble
Log *S* (Ali)	-13.04
Solubility	1.01e-10 mg/ml ; 9.02e-14 mol/l
Class	Insoluble
Log *S* (SILICOS-IT)	-9.48
Solubility	3.72e-07 mg/ml ; 3.32e-10 mol/l
Class	Poorly soluble

Pharmacokinetics	
GI absorption	Low
BBB permeant	No
P-gp substrate	Yes
CYP1A2 inhibitor	No
CYP2C19 inhibitor	No
CYP2C9 inhibitor	No
CYP2D6 inhibitor	No
CYP3A4 inhibitor	Yes
Log K_p (skin permeation)	-6.19 cm/s

Druglikeness	
Lipinski	No; 2 violations: MW>500, NorO>10
Ghose	No; 4 violations: MW>480, WLOGP>5.6, MR>130, #atoms>70
Veber	No; 2 violations: Rotors>10, TPSA>140
Egan	No; 2 violations: WLOGP>5.88, TPSA>131.6
Muegge	No; 5 violations: MW>600, XLOGP3>5, TPSA>150, Rotors>15, H-acc>10
Bioavailability Score	0.17

Medicinal Chemistry	
PAINS	0 alert
Brenk	2 alerts: isolated_alkene, more_than_2_esters
Leadlikeness	No; 3 violations: MW>350, Rotors>7, XLOGP3>3.5
Synthetic accessibility	10.00

Compound 24:

Molecule 2

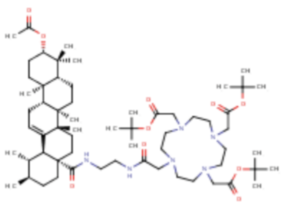

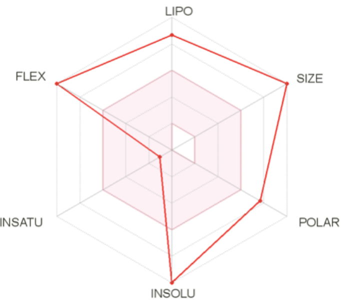

SMILES	O=C(CN1CCN(CCN(CCN(CC1)CC(=O)OC(C)(C)C)CC(=O)OC(C)(C)C)CC(=O)OC(C)(C)C)NCCNC(=O)[C@@]12CC[C@H]([C@@H]([C@H]2C2=CC[C@H]3[C@@]([C@@]2(CC1)C)(C)CC[C@@H]1[C@]3(C)CC[C@@H](C1(C)C)OC(=O)C)C)C

Physicochemical Properties	
Formula	$C_{62}H_{106}N_6O_{10}$
Molecular weight	1095.54 g/mol
Num. heavy atoms	78
Num. arom. heavy atoms	0
Fraction Csp3	0.87
Num. rotatable bonds	22
Num. H-bond acceptors	14
Num. H-bond donors	2
Molar Refractivity	324.19
TPSA	176.36 Å^2

Lipophilicity	
Log $P_{o/w}$ (iLOGP)	8.23
Log $P_{o/w}$ (XLOGP3)	9.65
Log $P_{o/w}$ (WLOGP)	6.53
Log $P_{o/w}$ (MLOGP)	3.75
Log $P_{o/w}$ (SILICOS-IT)	7.90
Consensus Log $P_{o/w}$	7.21

Water Solubility	
Log *S* (ESOL)	-11.26
Solubility	6.02e-09 mg/ml ; 5.50e-12 mol/l
Class	Insoluble
Log *S* (Ali)	-13.27
Solubility	5.90e-11 mg/ml ; 5.39e-14 mol/l
Class	Insoluble
Log *S* (SILICOS-IT)	-10.66
Solubility	2.40e-08 mg/ml ; 2.19e-11 mol/l
Class	Insoluble

Pharmacokinetics	
GI absorption	Low
BBB permeant	No
P-gp substrate	Yes
CYP1A2 inhibitor	No
CYP2C19 inhibitor	No
CYP2C9 inhibitor	No
CYP2D6 inhibitor	No
CYP3A4 inhibitor	Yes
Log K_p (skin permeation)	-6.13 cm/s

Druglikeness	
Lipinski	No; 2 violations: MW>500, NorO>10
Ghose	No; 4 violations: MW>480, WLOGP>5.6, MR>130, #atoms>70
Veber	No; 2 violations: Rotors>10, TPSA>140
Egan	No; 2 violations: WLOGP>5.88, TPSA>131.6
Muegge	No; 5 violations: MW>600, XLOGP3>5, TPSA>150, Rotors>15, H-acc>10
Bioavailability Score	0.17

Medicinal Chemistry	
PAINS	0 alert
Brenk	2 alerts: isolated_alkene, more_than_2_esters
Leadlikeness	No; 3 violations: MW>350, Rotors>7, XLOGP3>3.5
Synthetic accessibility	10.00
